# Supplementary material for: Protocol for a pragmatic cluster randomised controlled trial assessing the clinical effectiveness and cost-effectiveness of Electronic RIsk-assessment for CAncer for patients in general practice (ERICA)
Source: BMJ Open. 2023 Mar 20;13(3):e065232. doi: 10.1136/bmjopen-2022-065232 (PMC10030284; doi:10.1136/bmjopen-2022-065232)
Supplement: Supplementary data [file bmjopen-2022-065232supp001.pdf]

A pragmatic cluster randomised controlled trial assessing the clinical effectiveness and cost-effectiveness of electronic risk-assessment for cancer for patients in general practice (ERICA): Appendices

Appendix A. A simplified schema illustrating the pathways to a cancer diagnosis in the UK. The size of the arrow reflects the approximate proportion of cancers taking each route. The yellow central arrow represents where eRATs are expected to have an effect.

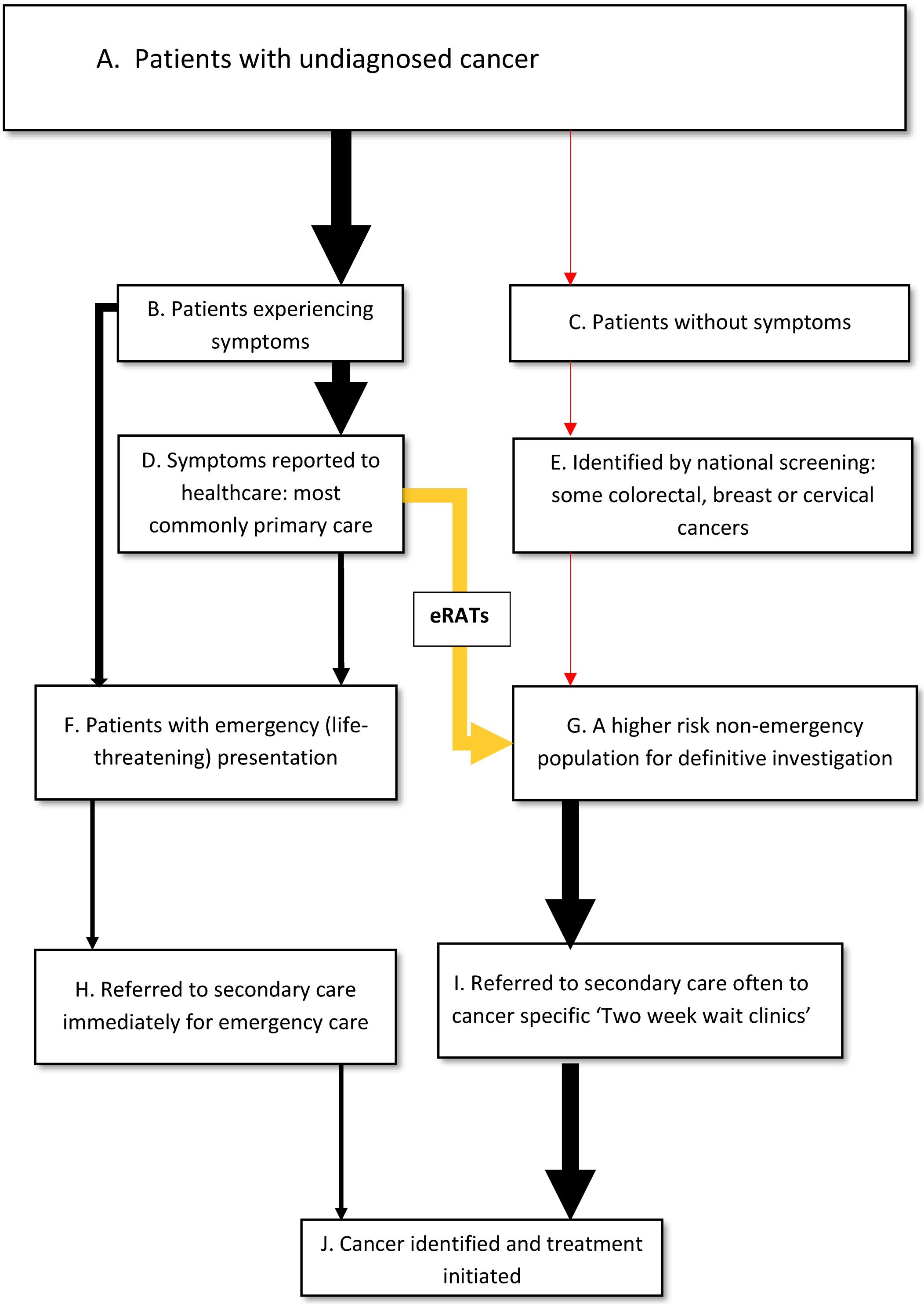

Appendix B. Managing practice splits and mergers in analyses

Although we will exclude practices that report imminent restructuring during recruitment, there may be unforeseen mergers or splits of practices. Where mergers and splits are concerned, this could mean, for example, that some of our practices who were in the control arm may merge with an intervention practice. Similarly, a non-trial practice may become part of a trial practice (intervention or control). Changes in practice size have implications on the denominator – the number of patients that each practice is likely to be contributing to our sample – and is a particular issue for three of our secondary outcome measures based on rates (cancer diagnosis rate, two-week wait referral rate, and adverse event rate). Importantly, however, this issue is not a problem for our primary outcome of staging.

We define a split and mergers as follows: Split – Where a population of patients registered to a single practice with a single practice code become registered with two or more individual practices with different practice codes. The practice codes of the new practices may be new codes (i.e. did not exist prior to the split) or one may inherit the original practice code (although this is not a requirement). The change in registration of patients must occur to a substantial number of patients and not at their request. Merger – Where a population of patients registered to one or more practices with different practice codes become registered at a single individual practice with a single practice code. The practice code of the new practice may be a new code (i.e., did not exist prior to the split) or it may inherit one of the original practice codes. A federation is not a "merger" in these terms.

Excluding practices who restructure during the trial may unnecessarily reduce our power. Therefore, we will try and accommodate changes in status. The Table outlines our approach. The assumption is that the change takes place at time T. Any practice which splits goes from X to Y and Z, and mergers are Z plus Y becoming X. Intervention practices are I, and comparison practices C.

Practice size fluctuations will be monitored in real time. Practice size data are freely and publicly available from NHS Digital and are updated monthly. Each month during the data collection, the trial statistician will download the practice size data and inspect size for all the practices in the trial (the statistician will remain blinded to outcome allocation). If the practice size differs by more than 10% the statistician will alert the trial manager, who will contact the research champion in the relevant practice to explore the reasons for this practice size change. Reasons (e.g., mergers, splits) will be recorded.

Table: managing changes in practice size – mergers and splits

<table>
<tr><td>Split or merger</td><td>X pre change</td><td>Y pre change</td><td>Z pre change</td><td>X post change</td><td>Y post</td><td>Z post</td></tr>
<tr><td rowspan="2">Split</td><td>I</td><td></td><td></td><td></td><td>I</td><td>I</td></tr>
<tr><td>C</td><td></td><td></td><td></td><td>C</td><td>C</td></tr>
<tr><td colspan="7">We will allow the daughter practices to withdraw from the trial if they desire, which would mean we lose Y or Z (or both). If daughter practices decide to withdraw, we will include data up to time T plus 2 months to allow for average diagnostic time to cancer.</td></tr>
<tr><td rowspan="5">Merger</td><td></td><td>I</td><td>I</td><td>I</td><td></td><td></td></tr>
<tr><td></td><td>I</td><td>C</td><td>I</td><td colspan="2" rowspan="2">There is likely to be wash over under these conditions, so the merged practice will be considered as I</td></tr>
<tr><td></td><td>I</td><td>Non-trial</td><td>I</td></tr>
<tr><td></td><td>C</td><td>Non-trial</td><td>C</td><td></td><td></td></tr>
<tr><td></td><td>C</td><td>C</td><td>C</td><td colspan="2"></td></tr>
</table>

We will manage changes in practice size at the data analysis stage of the trial. Where changes in list size of more than 10% within a month are seen, data for that practice will not be included in the analysis of rate outcomes from one month prior to the change. There are two exceptions to this; 1) splits where all the daughter practices remain in the trial and we continue to treat them as a single practice for rate analyses, 2) mergers where merged practices are in the same arm of the trial, and we will analyse them as a single practice from the start for rate analyses.

Appendix C

Health Economics

Intervention costings. The resources used in developing the training materials and videos (preparation and IT support) will be collected from the trial manager; nationally applicable unit costs will be applied. Estimates of the extent to which these videos are watched by practice staff will be based on information available from the website platform hosting the videos. Information on the resources use to install the eRATs onto the EMIS and SystmOne practice IT systems will be estimated in consultation with practice champions. These estimates will additionally aim to estimate: 1) the cost of installation in the trial and 2) the anticipated cost of future installation should eRATs be implemented nationally.

Health related quality of life and resource use. The Health Economics analysis will draw on the estimated number of imaging investigations (colonoscopies, sigmoidoscopies, upper gastro-intestinal endoscopies, chest x-rays, abdominal ultrasounds, and abdominal CT scans) using data from the Diagnostic Imaging Dataset available in the main trial as well as estimates of GP workload from the process evaluation. Practices will be offered remuneration of nearly £200 for the additional work.

To investigate whether the eRATs intervention was associated with a change in health-related quality of life using the EQ5D-5L and to provide more detailed information on primary care services and tests used, we will sample patients in the intervention arm who had a consultation where an eRAT alert occurred, and patients in the control arm who had a consultation where an eRAT alert would have occurred. We will strategically target practices in both trial arms who have either high, medium, or low two-week wait referral rates, matching the minimisation criteria in the main trial. It is anticipated that 15-20 patients per practice over a 2-week period will have a consultation with an eRAT alert. All patients who have an eRAT alert will be invited to complete a baseline questionnaire and a 3 month follow-up Health Economics questionnaire, as will equivalent patients in the control arm. We anticipate that 40% of patients will accept, and of these there will be 20% who do not respond. With a conservative estimate of a cluster size of five patients responding to the questionnaire, plususing an minimum clinically important difference of 0.1 for the EQ5D-5L (2) and a standard deviation of 0.23(3), with an inter-cluster correlation coefficient of 0.03 (4), and an estimated coefficient of variation of cluster size of 0.7, the sample size required to detect a between group difference with 90% power and alpha of 0.05 was 28 clusters (140 participants) per arm. Participants who agree to take part will receive the questionnaire as a hard copy, through the post, or electronically via email, depending on the participant's preference. Nationally applicable unit costs will be used for all community health and social care contacts (5) and secondary care services, tests and investigations will be costed using the National Schedule of Reference Costs 2016-2017. (6)

Decision Analytic Model

The modelling aims to predict the expected impact of a change in stage of diagnosis, and any resulting change in the distribution of cancer stage at diagnosis (intervention vs. control) over time, building on the published literature in this area.(7-10) The decision analytic models will not need to separately model the diagnostic phase, and we will take the trial's primary outcomes, stage at diagnosis (Stage 1-4 separately and not collated into Stage 1-2 and Stage 3-4), to model the longer term effects on survival, QALYs and secondary care costs.

Scenario analysis will be used to examine the impact on the results of multiple parameters changing simultaneously (based on *a priori* judgement about the combination of parameters to include).(11) Probabilistic sensitivity analysis will be used to explore the proportion of results that are considered cost-effective in relation to a given cost-effectiveness threshold and these results will be illustrated graphically using a cost-effectiveness acceptability curve.(12)

The study will follow the CHEERs guidelines for reporting cost-effectiveness studies and models,(13) and will discount both costs and outcomes at 3.5% as recommended by the National Institute of Health and Care Excellence.(14) Sensitivity analyses will examine alternative assumptions about the missing data mechanisms.(15)

Service Evaluation

We will draw upon published systematic reviews of Quality of Life measures, that are based on public preferences and measured in patients (as required by NICE guidelines (16) and that have been used for economic evaluation modelling studies.(17)

Appendix D

Service Delivery Modelling

Background and rationale

Cancer diagnosis has become one of the principal areas of focus and concern for the NHS in England.(18) For some time, NHS performance in both early diagnosis, delays in referral, and associated survival rates has been poor relative to our national aspirations and when compared with other first world countries. This has worsened during the COVID pandemic. In this context, many of the issues of concern are centred on key aspects of service delivery. How the NHS organises its services is often pivotal in determining the cost, feasibility, and effectiveness. For instance, factors such as workforce availability, prioritisation, service location, scale, and resources are fundamental to the performance of the NHS in delivering effective cancer services.

This component of the ERICA programme will investigate the key factors central to the organisation of NHS diagnostic services for cancer referrals. We will use a range of methods, both quantitative and qualitative, to analyse service delivery alternatives. Specifically, we will aim to build an economic model to assess the likely implications of different scenarios. Implementation of the eRAT diagnostic tool at primary care level is likely to impact directly on the follow-on pathway for cancer diagnosis (for example in terms of the volume and case mix of referred patients for diagnosis). Our model will

therefore provide an assessment of the likely effect of this impact in terms of costs and performance, and highlight any changes in organisation that might be implied by the introduction of the eRAT tool.

This research will run in parallel with the substantive work conducted for the controlled trial of eRAT implementation within ERICA. It will also liaise closely with the detailed and standard analysis of cost-effectiveness for disease progression (which is inherently abstracted from the service delivery aspects of care) in order to provide an added dimension to the cost-effectiveness outputs from the ERICA study as a whole.

Objectives

To build and populate a model of the cancer diagnostic pathway for England, in order to provide an assessment of the costs and effectiveness of different scenarios for service delivery. In particular, we will investigate the potential aspects relative to implementation of eRATs based on the study data collected from the ERICA trial. In addition, qualitative research with NHS staff in secondary care will be used to assess key areas central to successful implementation and sustainability.

Methods

A wide range of methods will be essential to fulfil the objectives of the work outlined here. Early work will include a literature search and survey of current systems for diagnostics in cancer. We will therefore conduct a systematic review of the related literature in the field and carry out a survey of current service delivery organisation across a range of settings. This work will aim to identify the key factors bearing on the organisation of services such a regional variation, metropolitan versus rural context, and population case mix differences.

Phase two work will aim to build a model in order to capture the key elements of service delivery for diagnostic services for cancer. This will explore a range of modelling approaches and test which is most suited to specific needs. For example, discrete event simulation, Systems Dynamics, geographic analysis, and Markov modelling will all be tested in terms of their relevance and appropriateness to specific requirements. In this context it is highly likely that different modelling tools will be relevant to the diverse needs of the study, so no single approach will be dominant.

Phase three will focus on the service delivery implications for the introduction of the eRAT diagnostic tool in primary care looking particularly at the potential knock-on effects in other areas of service.

In addition to our modelling work, we will use qualitative methods, such as problem structuring methods, soft systems mapping, to provide an assessment of some key elements of implementation.

Data

A wide range of data will be used to complete this component of the work. We will aim to integrate sources from across routinely collected datasets such as those listed below to construct our models: NHS activity data, Waiting time data, Reference cost data, Diagnostic Imaging Data (DIDs), Hospital Episode Statistics (HES), Workforce reference data, GP and hospital referral data, QOF data, Population data (e.g. ONS). In addition, we will aim to incorporate the primary data derived from the main ERICA study in order to model and assess the pathway impact from the use of eRATs. We will also use the outputs from the standard economic analysis as an input for the cost effectiveness of the service delivery modelling. Output from the qualitative research will also provide important data for informing the outputs of this work, for example in feeding into the recommendations and conclusions of the study.

Appendix E

Process Evaluation

Scope of process evaluation

The process evaluation work aims to identify and investigate the contextual factors that impact upon the effectiveness of the eRATs with a particular focus on intervention fidelity and GP engagement. The impact of the eRATs on the patients' experience of their GP consultation and their experiences of subsequent care will also be explored. It is underpinned by the COM-B framework for understanding behaviour change (19). This framework will outline the interactive nature of how the GP's capability (IT skill for using the eRATs), opportunity (eRAT prompts), and motivation (to do the training and use all the eRAT features) might influence their behaviour – i.e. ongoing use of the eRATs, symptom checker, coding of symptoms and changes to referral letters. We will use a mixed-methods approach to explore how the intervention was delivered (including fidelity and dose - if the eRATs were being used as intended and their degree of use across intervention practices and over time) and GP engagement with and acceptability of using the eRATs (GP's experiences of the eRATs).(20) For delivery, we will be particularly interested in fidelity of function. (21) GPs will be given clear training videos on how to use the eRATs and we will explore the extent to which GPs engaged with training as well as how they subsequently engaged with the software, and the GP's experiences of how it impacted on the GP-patient relationship in order to evaluate how they responded to the intervention.

Methods

Intervention fidelity and GP engagement (intervention arm only): Prior to the start of the intervention GPs require a minimum level of training in how to use the eRATs. Although the software is designed to be intuitive, a clinical system specific walkthrough for the two main functions of the eRATs (prompt and symptom checker) and FAQs will be available via separate videos. The research champion will be

given access to the videos and can disseminate the video content to all GPs in the practice (by showing the videos during a practice meeting, providing a demonstration themselves, or passing on the weblink). Once practices have started the data collection phase, we will invite up to 10 research champions to interview to discuss in depth their experiences of the set-up and training procedures and to explore whether their GPs have the capability, opportunity and motivation to use the eRATs. We will purposively sample research champions based on whether they are from a practice with a high, middle or low two-week wait referral rate, which software system their practice uses, their gender, and their level of experience in practice (10+ years vs. less than 10 years in practice).

Detailed eRAT usage can be captured for all IT systems. Usage will be captured in two ways – i) via a central log and ii) via local ‘at practice’ reports. For i), usage logs will be routinely and automatically sent from the practice to the Informatica ‘digital warehouse’ and will contain anonymised, practice-level data for each eRAT including reports of: how many times the prompt was shown, how many times the symptom checker was used, the number of times the symptoms were changed during use of the symptom checker, the length of time the symptom checker was open for, and whether clinical guidance was accessed from the eRAT. These centrally reported logs will be available on a monthly basis throughout the course of the trial and will be securely sent from Informatica to the research team who will add the data to the trial database.

For ii), usage will be examined via reports run locally at each practice. These reports include individual patient level data outlining which eRAT was triggered, the patient’s risk score on the eRAT, when the symptom checker was opened and closed, patient’s age and sex, and a list of possible eRAT symptoms and whether they were changed. These reports contain depersonalised (pseudo-anonymised) data. As it is possible to potentially identify the patient via the practice ID number we will ask practices to make a copy of the report, add in a new patient study ID variable (e.g., p1, p2, p3, etc) and save it to the practice computer. We will then ask them to send a copy to the trial tram with the original practice ID number removed. They will also send the file with a predetermined practice ID number. These measures should ensure the data is anonymised. The local at practice reports will be securely and electronically transferred to a secure Exeter CTU computer.

Intervention fidelity (Intervention and control). We will ask all research champions in the intervention practice to complete a short questionnaire (online via a secure, University approved provider) detailing their experience of installing software, using the eRATs, and whether alternative risk tools have also been used. We will ask research champions at control practices their experiences of being in the trial and whether they have started using any cancer risk tools. The questionnaires will be

completed at two time points – i) within 12 months of the start of the intervention; ii) at the end of the data collection period.

For identifying GPs to interview, we will use maximum variation purposive sampling (sampling on practice two-week wait referral rate (high vs. medium vs. low); software system used, gender, length of time in practice (10+years vs. < 10 years), and working status (part time vs full time)) and expect to interview up to 18 GPs from intervention practices to ask them about their experience of the eRATs including the training provided, any impacts on the consultation and their clinical decision making, as well as any changes in symptom coding behaviour. We will invite GPs to interview after the intervention has been running for at least 3 months. Written information will be provided about the interview study and written consent will be taken prior to the interview and will be verbally confirmed before the interview commences. Interviews will be audio-recorded and carried out by telephone, face-to-face (only if it is safe to do so), or over the internet (e.g., Zoom or MS Teams) depending on the GP's preference, by members of the research team using a pre-defined topic guide that focuses on their training and capability to use eRATs, their opportunity to use the eRATS over the study period and their motivation to continue using the system. If a face-to-face interview is chosen (and safe to perform), interviews will take place in a private room at the practice. The researcher will comply with the lone worker policy, ensuring that have a 'buddy' within the research team monitoring their activities and whereabouts. The interviews may raise sensitive issues such as workload and GP overburden or burnout: the interview study information sheet will provide appropriate sources for accessing confidential support. GPs will be reminded that they have the right to not answer any question, stop the interview or withdraw from the interview study; if there is insufficient time to fully discuss issues GPs will be offered a follow-up time to complete the interview.

GP coding behaviour: It is possible that the eRATs will impact GP coding behaviour - GP coding behaviour for cancer specific symptoms may increase; this would cause a minor increase in triggering of eRATs. We will explore the impact of eRATs on coding behaviour in the interviews (see above) and, resources permitting, will also examine the impact on coding rates using the following approach. We will purposively sample 12 intervention practices and 12 control practices in the South/South West of England based on two-week wait referral rate (i.e., 4 low, 4 moderate, 4 high referring practices) and which software system is being used. In the first instance we will invite practices who are participating in the nested study to support this work. If insufficient numbers agree, we will approach other practices who are not participating in the nested studies. We will explore the rate of coding of the most frequent symptom for each eRAT cancer in the study that underpins that particular cancer (e.g. cough, abdominal pain, haematuria)(22-25) for a month in the first three months of entry into ERICA, and for the same calendar month a year and two years later (as some symptoms have seasonal

variation). This will be performed retrospectively, by the search code being given to the research champion, who will arrange for the search to be conducted in the practice. The results of the search will be emailed to the research team.

Patient experience of care: We will adopt a phased, targeted recruitment strategy with an aim to purposively sample up to (based on two-week rate referral rate (low vs. medium vs. high); gender, age (40-60 vs. 60+)) 32 patients from the intervention arm. We will approach five practices at a time (and expect to recruit around 20 practices to reach the target number of participants), to ensure that we can interview participants in a timely manner.

The in-practice eRAT reports are the mechanism by which we will be able to identify individuals to invite to participate in the activities associated with the process evaluation. The local (at practice) reporting mechanism will allow the research team to identify individuals for whom the eRATs were used and thus who are potentially eligible to participate in a semi-structured interview. Purposive sampling will take place – practices will hold the master eRAT report containing both the patients practice ID number and the new patient study ID. The research team will let the practice know the patient study IDs for those whom an invitation letter will be sent. Practices will be offered remuneration of nearly £200 for the additional work.

Via the GP practice, the research team will send out a letter and information booklet to the identified patients to invite participation in an interview to discuss their experience of care. We will adopt a longitudinal case study design (26) – patients' care pathways will differ, some will receive referrals into secondary care for investigations and tests, while some will be on a 'watch and wait' plan, revisiting their GP at an agreed interval. Some patients will have tests for cancer and the test will indicate that there is no cancer (false positives) whereas some patients will be diagnosed with cancer. So that we can fully capture all patient groups at different stages of their care, individuals will be invited for repeat interviews at regular intervals (i.e., at least one month apart and no more than 3 interviews within 12 months).

We aim to perform the first interview within one month of the consultation in which an eRAT was triggered. Written information about the interview study will have been provided and written informed consent will be taken prior to all interviews, and will be verbally confirmed before the interview commences. Interviews will be audio-recorded and carried out by members of the research team using pre-defined topic guides. The initial interview will be conducted face-to-face at the participant's home or via video conferencing software such as MS Teams at a time convenient for the participant, with any subsequent interview conducted either face-to-face, over the phone, or via video

conference software, depending upon the participant's preference. We will monitor the progression of the Covid-19 pandemic and fully adhere to government advice around social distancing and travel. We will not put the research team or participants at risk and will primarily conduct interviews online. If it is safe to conduct face-to-face interviews, the researcher will comply with the lone worker policy, ensuring that have a 'buddy' within the research team monitoring their activities, whereabouts and expected completion time. The interviews may raise anxiety or concerns related to uncertainty about diagnosis during the referral and investigation period or the watch and wait period; or psychological distress associated with a cancer diagnosis or a false-positive result. The interview study information sheet will provide appropriate sources for accessing confidential support and patients will be reminded that they have the right to not answer any question, stop the interview or withdraw from the interview study.

Management of adverse consequences

As a result of being referred for tests or investigations there is a risk of an adverse incident. If referral rates do increase as a result of access to eRATs, there is an increased risk of an adverse event (AE) to patients of practices allocated to the intervention. We are not routinely tracking individuals throughout the trial and there is no mechanism for monitoring any AEs as a result of referral. However, psychological distress may be a consequence of referral. Individuals for whom cancer is diagnosed at an early stage may be relieved by the diagnosis and see the psychological distress as justifiable. Individuals for whom a referral does not lead to a diagnosis of cancer (false positives) may have undergone unnecessary psychological distress. Our process evaluation work will help us to understand the extent of this and its potential impact on the individuals' life.

During interviews, patients may report being distressed – either as a result of research activity or as a result of their health, and events in their private lives. Should such a situation arise, the researchers will implement the trial risk protocol and manage the participant in accordance with this policy. Participants will be reminded that they have the right to not answer any question, stop the interview or withdraw from the interview study. Under high-risk situations (e.g. where there is perceived immediate risk to a participant's health), the study team may be required to break confidentiality, to inform appropriate authorities who will need to provide essential care services. We will also signpost participants to sources of support. This information will be outlined in the Participant Information Sheet. Participants will be informed of possible benefits and known risks of participation in the interviews by means of a Patient Information Sheet and through discussion with the research team. Written consent will be obtained immediately prior to the interview study.

There are minimal risks to researchers as most interviews will take place in the GP practices or by telephone/online; however, if a home visit is undertaken to interview patient participants the researcher will follow the lone worker policy: researchers will make sure that their whereabouts, contact telephone number and estimated time of return are known to their colleagues and/or manager. Researchers will also have the opportunity to debrief with a senior colleague on the research team should they need any support after conducting an interview; this debrief may be in person or by telephone.

Analysis

For the quantitative results the individual data sources will be summarised descriptively, including a summary of data completeness. For the qualitative data we will adopt a framework approach (27) which allows the inclusion of key concepts and ideas identified from the literature, alongside themes emerging from the data. The framework approach produces a structured output matrix, with cells of data organised by practice and by code (a descriptive label applied to a section of transcript).

At least two researchers will work on the analysis. Interviews will be audio recorded, transcribed and anonymised. Data familiarisation will be achieved through the listening to and reading of interview recordings and transcripts. Transcripts will be imported into the qualitative data analysis software package NVivo 11 (QSR International) to facilitate data management, sharing and development of a coding framework. A proportion of the interview transcripts will be coded by each researcher. The 'constant comparative method' (28) will be utilised: each incident in the data will be compared with other incidents for similarities and differences and any 'negative cases', where a case does not fit the pattern or cannot be explained by the emerging analysis, will be explored and recorded. Following this initial coding, a PPIE meeting (one for the GP interviews and one for the patient interviews) will be held to discuss the emerging themes from the interviews, and to gain alternative perspectives from the PPIE group on those themes. Following the PPIE meeting, the analytical framework will be developed, incorporating researcher and PPIE perspectives on the results, with a final set of themes and codes being agreed upon.

The analytical framework will be applied to all interview transcripts; one researcher will index all transcripts, with a second researcher indexing a proportion, to check the reliability of the indexing and to ensure that the themes of the framework are being interpreted consistently. Any differences in interpretation will be discussed between the two researchers. Following the indexing process, data will be charted into the structured output matrix, which will summarise the data on each theme from all transcripts. A subsequent meeting of the PPIE group will be held once all of the results from the process evaluation have been gathered to gain a users' perspective of the global findings.

The final step in the process evaluation analysis will be to integrate results from the various mixed method data sources using a triangulation protocol(29) to give a more complete picture once individual data sources have been individually analysed. We plan to create a summary matrix, known as a convergence coding matrix, which summarises the findings from each data source after assessing whether the findings are in agreement, partial agreement or no agreement, or whether the data source is silent for the finding under consideration i.e. when a theme or finding arises from one data set but not another.

Reporting

The process evaluation results will be briefly summarised for inclusion in the main trial report and publication, separate dissemination (reports, presentations and publications) will provide further details of the process evaluation findings.

Appendix F

GP Workload

Background and rationale

GPs manage a high and rising workload of increasingly complex patient care with many competing demands to attend to within ten-minute consultations. (30) This, combined with ongoing recruitment and retention challenges, has contributed to a GP workforce 'crisis'. (31-36) The workload implications for GPs of using electronic tools such as eRATs during consultations is unclear.(37) ERICA provides an opportunity to examine whether the use of eRATs by GPs, and the possible subsequent discussion of cancer risk with patients, may impact consultation length and patient 'flow' through consulting sessions. This nested study aims to explore, in terms of consultation time, the impact of GPs using eRATs on GP workload and patient 'flow' through consulting sessions. It will also explore workload in the week following the index consultation in which an eRAT was activated, when relevant letters may be generated, referrals made, investigations followed through, or clinical discussions engaged in.

Objectives

The specific objectives in respect of consultations and sessions are:

(i) to measure and compare the duration of consultations and sessions in which an eRAT has been activated with consultations where eRATs have not been activated;

(ii) to measure and compare the duration of subsequent consultations in the same session after an eRAT has been activated with consultations in sessions where eRATs have not been activated;

(iii) to explore the frequency of interactions with patients' medical records by a GP in the week following a consultation during which an eRAT was activated.

Methods

An observational quantitative study will be conducted in a sub-sample of ERICA intervention practices to examine the durations of consultations and consulting sessions in which eRATs are activated.

Sample size

The basis for the sub-study sample size calculation is on the number of consultations likely to occur over a two-week period within ERICA practices, in which an eRAT will be 'activated' (i.e. an eRAT prompt is shown and/or clinician uses an eRAT symptom checker). A number of assumptions are of note:

The first assumption is that a half-day GP consulting session, typically lasting four hours and comprised of ten-minute consultations, would be associated with a total of 24 consultations. Second, practices have an average headcount of seven GPs (informed by GP workforce data from NHS Digital). (38) Third, a GP is assumed to work an average of 6.7 half-day consulting sessions per week. (39) An average practice would therefore provide a total of 1,126 GP consultations per week.

Accurate estimations of how often an eRAT will be activated, are not yet established in previous research on usage of cancer decision tools in UK general practice. (40,41) Two clinical members of the research team have estimated that an eRAT may be expected to be activated once per GP, per week. This estimate would suggest that approximately 15% of consulting sessions will involve a consultation where the eRAT tool was activated.

The standard deviation for both the length of a consultation and of a whole consulting session from previous literature was four minutes and 20 minutes respectively. (42-44) Project team discussion concluded that a minimally important difference in time for an individual consultation would be between two and five minutes; for a consulting session this minimally important difference would be approximately 10 minutes.

Statistical power to detect a time difference of between two and five minutes in eRAT consultations versus non-eRAT consultations is also in excess of >80%, even if eRATs are observed to have been activated in just 1:40 consulting (2.5% of sessions), the basis of the most conservative estimate. The

power to detect a difference of 10 minutes in sessions where eRATs have been activated compared with sessions where eRATs have not been activated is >80%, even if eRATs affect only 2.5% of sessions. A two-week observation period would provide sufficient data and power to detect differences in the length of consultations and sessions where an eRAT is activated and those where an eRAT is not activated.

Outcome measures

Primary outcome

The primary outcome is the length of time (in minutes) of consultations. These will be consultations during which an eRAT is activated and also those during which an eRAT is not activated. For the purposes of this sub-study, a consultation is defined as starting when the patient's electronic medical record is opened by a GP, for the purpose of conducting either a face-to-face or telephone/video interaction with the patient, and ending when the record is closed. Home visits will be excluded due to difficulty in accessing accurate time information. Consultations with health professionals who would not make referral decisions (e.g. practice nurses, physiotherapists, pharmacists, healthcare assistants) will also be excluded.

Secondary outcomes

In addition to our primary outcome, we propose to examine the following secondary outcomes:

- The length of time (in minutes) of consulting sessions. For the purposes of this study, a session is defined as a half-day period comprised of individual patients' pre-booked or same-day consultations. The half-day periods are typically 'morning' or 'afternoon', although some practices offer early morning and evening sessions as well. (45)
- The number of instances of opening a patient's electronic medical record in the week following an eRAT being activated.

Practice recruitment

An initial pilot in up to three ERICA intervention practices will be undertaken and plans for data collection methods revisited at that point. Practices will be approached by an invitation email and provided with an information sheet detailing the nature of the study and providing contact details of the researcher. No individual patients will be recruited.

A note on practice recruitment to the nested studies: We expect to recruit up to 91 practices across the nested studies (56 in the health economics nested study, up to 20 in the process evaluation and up to 15 in the sub-study on GP workload) practices. Practices will only be asked to help with one of the health economic nested study, the process evaluation nested study, or the GP workload sub-study.

Data collection

Identifying consultations where an eRAT is activated

The Process Evaluation describes earlier how a local 'at practice' report will be run for practices in order to collect patient-level data on eRATs usage. This report will be run for practices recruited to this nested study, covering a two-week period.

Measuring durations of consultations and sessions

The eRATs usage report will provide the start and end time of the tool usage, but not the duration of a consultation. A further search function (developed within SystmOne for this nested study) will provide data on the timings of all consultations occurring between two dates (referred to as the 'appointments report'). The consultations identified in the eRAT usage report will be cross-referenced with the consultations in the appointments report. A variable will be added to denote which consultations involved an eRAT being activated and which did not.

Measuring workload in the week following an eRAT being activated

The eRATs usage report will identify the relevant patient records for which an audit will be run in SystmOne. The audit will provide data on instances of the records being opened and closed by practice staff during the week following the index activation of an eRAT.

Data analysis

Data will be analysed in Stata. Descriptive statistics summarising participating practices and GPs will be presented. Although practice level data will be presented, it will be anonymised (e.g. practice A, B, etc) to protect the identities of individual practitioners or practices.

The primary analysis of the durations of consultations in which an eRAT is activated, will take the form of a mixed-effects linear regression with random intercepts to account for clustering within GPs and for GPs clustering within practices. This regression will adjust for consulting GP, time of day, day of week, and consultation mode (face-to-face, telephone, video). Residuals will be checked for normality. As duration data are typically not normally distributed, the data will be transformed if needed, using log transformation. Bootstrapping of the data will also be undertaken if needed. Similar mixed-effects linear regression models with random intercepts will also be performed for secondary outcomes; the duration of consulting sessions, and the number of instances of opening a patient's electronic medical record in the week following an eRAT being activated. For all models where duration is the outcome linear models will be used, but for the count of opening medical records Poisson models will be used.

Governance and ethical considerations

Consent

Individual patient consent is not sought within ERICA for the running of the eRAT usage report. The reports in SystmOne, described for this nested study, will not contain identifiable patient data nor clinically sensitive information and so patient consent for these reports will also not be sought.

Data protection/management and confidentiality

The eRAT usage report and the SystmOne reports will contain pseudo-anonymised data: a patient identifier. However, the reports will contain variables denoting date, time and consulting GP, which will allow cross-referencing, so practices will be asked to delete the patient identifier before sending the report securely and electronically to a secure Exeter CTU computer using a predetermined practice ID number. These measures will ensure the data are anonymised. In the event that the researcher visits the practice to run the SystmOne reports, the files will be anonymised in the same way before the researcher leaves. Practices will keep the original ‘master’ report files containing the patient’s practice computer ID.

Finance

The additional work for the nested study, outside of ERICA costs, is for practices to run the reports in SystmOne and send the report files securely to the researcher. Alternatively, the researcher will visit the practice to run the reports, which may require time of a practice administrator or manager for logging in to the clinical system and orientation. In both scenarios, this time would be covered by nested study research costs at a rate of £50 per hour, and each practice will be offered reimbursement for up to 2 hours. Travel costs for the researcher to visit practices where needed are estimated at £0.45 per mile for a 75 mile round-trip per practice (South West).

References

1. Neal RD, Din NU, Hamilton W, Ukoumunne OC, Carter B, Stapley S, et al. Comparison of cancer diagnostic intervals before and after implementation of NICE guidelines: analysis of data from the UK General Practice Research Database. Br J Cancer. 2014;110(3):584-92.
2. Pickard AS, Neary MP, Cella D. Estimation of minimally important differences in EQ-5D utility and VAS scores in cancer. Health and quality of life outcomes. 2007;5:70-.
3. Kind P, Hardman G, Macran S. UK population norms for EQ-5D. Centre for Health Economics, University of York; 1999.
4. Man M-S, Chaplin K, Mann C, Bower P, Brookes S, Fitzpatrick B, et al. Improving the management of multimorbidity in general practice: protocol of a cluster randomised controlled trial (The 3D Study). BMJ Open. 2016;6(4):e011261.
5. Curtis L. Unit Costs of Health and Social Care 2020 [Available from: https://www.pssru.ac.uk/project-pages/unit-costs/unit-costs-2020/]. 2020.
6. Department of Health and Social Care. NHS reference costs 2019 to 2020 [Available from: https://improvement.nhs.uk/resources/reference-costs/]. 2020.
7. Caro JJ, Briggs AH, Siebert U, Kuntz KM. Modeling good research practices--overview: a report of the ISPOR-SMDM Modeling Good Research Practices Task Force-1. Medical decision making : an international journal of the Society for Medical Decision Making. 2012;32(5):667-77.
8. Siebert U, Alagoz O, Bayoumi AM, Jahn B, Owens DK, Cohen DJ, et al. State-Transition Modeling:A Report of the ISPOR-SMDM Modeling Good Research Practices Task Force–3. Medical Decision Making. 2012;32(5):690-700.
9. Schwander B, Hiligsmann M, Nuijten M, Evers S. Systematic review and overview of health economic evaluation models in obesity prevention and therapy. Expert review of pharmacoeconomics & outcomes research. 2016;16(5):561-70.
10. Wang BC, Furnback W. Modelling the long-term outcomes of bariatric surgery: A review of cost-effectiveness studies. Best practice & research Clinical gastroenterology. 2013;27(6):987-95.
11. Andronis L, Barton P, Bryan S. Sensitivity analysis in economic evaluation: an audit of NICE current practice and a review of its use and value in decision-making. Health Technol Assess. 2009;13(29):iii, ix-xi, 1-61.
12. Fenwick E, O'Brien BJ, Briggs A. Cost-effectiveness acceptability curves--facts, fallacies and frequently asked questions. Health economics. 2004;13(5):405-15.
13. Husereau D, Drummond M, Petrou S, Carswell C, Moher D, Greenberg D, et al. Consolidated Health Economic Evaluation Reporting Standards (CHEERS) statement. Value Health. 2013;16(2):e1-5.
14. NICE. Guide to the Methods of Technology Appraisals, Section 5.6 Discounting [Available from: https://www.nice.org.uk/process/pmg9/chapter/the-reference-case#discounting]. 2013.
15. Faria R, Gomes M, Epstein D, White IR. A guide to handling missing data in cost-effectiveness analysis conducted within randomised controlled trials. PharmacoEconomics. 2014;32(12):1157-70.
16. Brazier J, Longworth L. NICE DSU Technical Support Document 8: An introduction to the measurement and valuation of health for NICE submissions. In: Health, editor. 2011.
17. Tengs TO, Wallace A. One thousand health-related quality-of-life estimates. Medical care. 2000;38(6):583-637.
18. NHS England. (2019) The NHS Long Term Plan [Available from: https://www.longtermplan.nhs.uk/.
19. Michie S, van Stralen MM, West R. The behaviour change wheel: a new method for characterising and designing behaviour change interventions. Implementation science : IS. 2011;6:42-.
20. Grant A, Treweek S, Dreischulte T, Foy R, Guthrie B. Process evaluations for cluster-randomised trials of complex interventions: a proposed framework for design and reporting. Trials. 2013;14:15.
21. Hawe P, Shiell A, Riley T. Complex interventions: how "out of control" can a randomised controlled trial be? BMJ (Clinical research ed). 2004;328(7455):1561-3.
22. Shephard EA, Stapley S, Neal RD, Rose P, Walter FM, Hamilton WT. Clinical features of bladder cancer in primary care. The British journal of general practice : the journal of the Royal College of General Practitioners. 2012;62(602):e598-604.
23. Shephard E, Neal R, Rose P, Walter F, Hamilton WT. Clinical features of kidney cancer in primary care: a case-control study using primary care records. The British journal of general practice : the journal of the Royal College of General Practitioners. 2013;63(609):e250-5.

24. Hamilton W. The CAPER studies: five case-control studies aimed at identifying and quantifying the risk of cancer in symptomatic primary care patients. Br J Cancer. 2009;101 Suppl 2:S80-6.
25. Stapley S, Peters TJ, Neal RD, Rose PW, Walter FM, Hamilton W. The risk of oesophago-gastric cancer in symptomatic patients in primary care: a large case-control study using electronic records. Br J Cancer. 2013;108(1):25-31.
26. Yin R. Case Study Research: Design and Methods. London: Sage Publications 2003.
27. Ritchie J, Spencer L, O'Connor W. Carrying out Qualitative Analysis. In: Ritchie J, Lewis J, editors. Qualitative Research Practice, A guide for Social Science Students and Researchers London: Sage; 2003.
28. Corbin J, Strauss A. Basics of qualitative research: Techniques and procedures for developing grounded theory. London: Sage publications; 2014.
29. O'Cathain A, Murphy E, Nicholl J. Three techniques for integrating data in mixed methods studies. BMJ. 2010;341:c4587.
30. Hobbs FDR, Bankhead C, Mukhtar T, Stevens S, Perera-Salazar R, Holt T, et al. Clinical workload in UK primary care: a retrospective analysis of 100 million consultations in England, 2007-14. Lancet 2016;387:2323-30. http://dx.doi.org/10.1016/S0140-6736(16)00620-6
31. Baird B, Charles A, Honeyman M, Maguire D, Das P. Understanding pressures in general practice: The Kings Fund; 2016.
32. Campbell J CR, Sansom A. Retaining the experienced GP workforce in direct patient care (ReGROUP): final report. Exeter: University of Exeter Medical School; 2015.
33. Sansom A, Terry R, Fletcher E, Salisbury C, Long L, Richards SH, et al. Why do GPs leave direct patient care and what might help to retain them? A qualitative study of GPs in South West England. BMJ Open 2018;8:e019849. http://dx.doi.org/10.1136/bmjopen-2017-019849
34. Roland M, Everington S. Tackling the crisis in general practice. BMJ 2016;352:i942. http://dx.doi.org/10.1136/bmj.i942
35. Fletcher E, Abel GA, Anderson R, Richards SH, Salisbury C, Dean SG, et al. Quitting patient care and career break intentions among general practitioners in South West England: findings of a census survey of general practitioners. BMJ Open 2017;7:e015853. http://dx.doi.org/10.1136/bmjopen-2017-015853
36. Walker B, Moss, C., Gibson, J., Sutton, M., Spooner, S., Checkland, K. Tenth National GP Worklife Survey. Manchester: Policy Research Unit in Commissioning and the Healthcare System Manchester Centre for Health Economics; 2019.
37. Fletcher E, Burns, A., Wiering, B., Lavi, D., Shephard, E., Hamilton, W., Campbell, J. L., Abel, G. Workload and workflow implications associated with the use of electronic risk assessment tools used by health professionals in general practice: a scoping review. In: University of Exeter Medical School; 2022.
38. NHS Digital. General Practice Workforce 30 September 2019. NHS Digital; 2019. URL: https://digital.nhs.uk/data-and-information/publications/statistical/general-and-personal-medical-services/final-30-september-2019#resources (Accessed 11/02/20).
39. Gibson J, Sutton M, Spooner S, Checkland K. Ninth National GP Worklife Survey 2017. In; 2017.
40. Moffat J, Ironmonger L. Clinical Decision Support Tool for Cancer (CDS) Project evaluation report to the Department of Health; 2014.
41. Robinson R, Poirier V, Watson S. Using Cancer Decision Support Tools to support the early diagnosis of cancer; 2017.
42. Salisbury C, Procter S, Stewart K, Bowen L, Purdy S, Ridd M, et al. The content of general practice consultations: cross-sectional study based on video recordings. British Journal of General Practice 2013;63:e751. http://dx.doi.org/10.3399/bjgp13X674431
43. Elmore N, Burt J, Abel G, Maratos FA, Montague J, Campbell J, et al. Investigating the relationship between consultation length and patient experience: a cross-sectional study in primary care. Br J Gen Pract 2016;66:e896-e903. http://dx.doi.org/10.3399/bjgp16X687733
44. Orton PK, Pereira Gray D. Factors influencing consultation length in general/family practice. Fam Pract 2016;33:529-34. http://dx.doi.org/10.1093/fampra/cmw056
45. NHS. Working life (GP). NHS; 2022. URL: https://www.healthcareers.nhs.uk/explore-roles/doctors/roles-doctors/general-practice-gp/working-life (Accessed 2022).
