# Supplementary material for: Protocol for a pragmatic cluster randomised controlled trial assessing the clinical effectiveness and cost-effectiveness of Electronic RIsk-assessment for CAncer for patients in general practice (ERICA)
Source: BMJ Open. 2023 Mar 20;13(3):e065232. doi: 10.1136/bmjopen-2022-065232 (PMC10030284; doi:10.1136/bmjopen-2022-065232)
Supplement: Author's manuscript [file bmjopen-2022-065232.draft_revisions.pdf]

BMJ Open is committed to open peer review. As part of this commitment we make the peer review history of every article we publish publicly available.

When an article is published we post the peer reviewers' comments and the authors' responses online. We also post the versions of the paper that were used during peer review. These are the versions that the peer review comments apply to.

The versions of the paper that follow are the versions that were submitted during the peer review process. They are not the versions of record or the final published versions. They should not be cited or distributed as the published version of this manuscript.

BMJ Open is an open access journal and the full, final, typeset and author-corrected version of record of the manuscript is available on our site with no access controls, subscription charges or pay-per-view fees (http://bmjopen.bmj.com).

If you have any questions on BMJ Open's open peer review process please email
info.bmjopen@bmj.com

BMJ Open

Protocol for a pragmatic cluster randomised controlled trial assessing the clinical effectiveness and cost-effectiveness of electronic risk-assessment for cancer for patients in general practice (ERICA)

Journal:	*BMJ Open*
Manuscript ID	bmjopen-2022-065232
Article Type:	Protocol
Date Submitted by the Author:	06-Jun-2022

Complete List of Authors:	Hamilton, Willie; University of Exeter, Primary Care Diagnostics<br>Mounce, Luke; University of Exeter, Institute of Health Research<br>Abel, Gary; University of Exeter, University of Exeter Medical School (Primary Care)<br>Dean, Sarah; PenCLAHRC University of Exeter Medical School,<br>Campbell, John; University of Exeter, Primary Care<br>Warren, Fiona; University of Exeter Medical School, Institute of Health Research<br>Spencer, Anne; University of Exeter Medical School, Health Economics<br>Medina-Lara, Antonieta; University of Exeter Medical School, Health Economics Group<br>Pitt, Martin; University of Exeter, University of Exeter: Medical School<br>Shephard, Elizabeth; University of Exeter,<br>Shakespeare, Marijke; University of Exeter Medical School, Primary Care<br>Fletcher, Emily; University of Exeter Medical School, Primary Care Research Group<br>Mercer, Adrian; University of Exeter Medical School, Primary Care<br>Calitri, Raff; University of Exeter Medical School, Primary Care

Keywords:	HEALTH SERVICES ADMINISTRATION & MANAGEMENT, Risk management < HEALTH SERVICES ADMINISTRATION & MANAGEMENT, PRIMARY CARE, Clinical trials < THERAPEUTICS

SCHOLARONE™
Manuscripts

Protocol for a pragmatic cluster randomised controlled trial assessing the clinical effectiveness and cost-effectiveness of electronic risk-assessment for cancer for patients in general practice (ERICA)

Willie Hamilton,[1] Luke Mounce, [1] Gary Abel, [1] Sarah Dean,[1] John Campbell, [1] Fiona Warren,[1] Anne Spencer, [1] Antonieta Medina-Lara, Martin Pitt, [1] Elizabeth Shephard,[1] Marijke Shakespeare, [1] Emily Fletcher, [1] Adrian Mercer,[1] Raff Calitri.[1]

[1]University of Exeter, St Luke's campus, Exeter, EX1 2LU

Willie Hamilton, Professor of Primary Care Diagnostics: w.hamilton@exeter.ac.uk, ORCID ID 0000-0003-1611-1373 (Corresponding author)

Luke Mounce, Research Fellow: l.t.a.mounce@exeter.ac.uk, ORCID ID 0000-0002-6089-0661

Gary Abel, Associate Professor: g.a.abel@exeter.ac.uk, ORCID ID 0000-0003-2231-5161

Sarah G Dean, Professor in Psychology Applied to Rehabilitation and Health: s.dean@exeter.ac.uk, ORCID ID 0000-0002-3682-5149

John Campbell, Professor of General Practice and Primary Care: john.compbell@exeter.ac.uk, ORCID ID 0000-0002-6752-3493

Fiona Warren, Senior Lecturer in Medical Statistics: f.c.warren@exeter.ac.uk, ORCID ID 0000-0002-3833-0182

Anne Spencer, Associate Professor: a.e.spencer@exeter..ac.uk ORCID ID 0000-0002-8163-3103

Antonieta Medina-Lara, Associate Professor in Health Economics: a.medina-lara@exeter.ac.uk, ORCID ID 0000-0001-7325-8246

Martin Pitt, Professor of Applied Healthcare Modelling and Data Science: m.pitt@exeter.ac.uk

Elizabeth Shephard, Research Fellow: e.a.shephard@exeter.ac.uk, ORCID ID 0000-0002-3610-3680

Marijke Shakespeare, Trial coordinator: m.shakespeare@exeter.ac.uk

Emily Fletcher, Research Fellow: e.fletcher@exeter.ac.uk, ORCID ID 0000-0003-1319-3051

Adrian Mercer, PPIE Representative, zante256@gmail.com

Raff Calitri, Research Fellow and Trial Manager, r.calitri@exeter.ac.uk, ORCID ID 0000-0003-0889-4670

For peer review only

Abstract

Introduction. The UK has worse cancer outcomes than most comparable countries, with a large contribution attributed to diagnostic delay. Electronic risk assessment tools (eRATs) have been developed to identify primary care patients with a ≥2% risk of cancer using features recorded in the electronic record.

Methods and analysis. This is a pragmatic cluster randomised controlled trial in English primary care. Individual general practices will be randomised in a 1:1 ratio to intervention (provision of eRATs for six common cancer sites) or to usual care. The primary outcome is cancer stage at diagnosis, dichotomised to stage 1 or 2 (early) or stage 3 or 4 (advanced) for these six cancers, assessed from national cancer registry data. Secondary outcomes include stage at diagnosis for a further six cancers without eRATs, use of urgent referral cancer pathways, total practice cancer diagnoses, routes to cancer diagnosis, and 30-day and 1-year cancer survival. Economic and process evaluations will be performed along with service delivery modelling. The primary analysis explores the proportion of cancer patients with early stage at diagnosis. The sample size calculation used an odds ratio of 0.8 for a cancer being diagnosed at advanced stage in the intervention arm compared with the control arm, equating to an absolute reduction of 4.8% as an incidence-weighted figure across the six cancers. This requires 530 practices overall, with the intervention active from April 2022 for 2 years.

Ethics and dissemination. The trial has approval from London City & East Research Ethics committee, reference number 19/LO/0615; protocol version 5.0, 9th May 2022. It is sponsored by the University of Exeter. Dissemination will be by journal publication, conferences, use of appropriate social media and direct sharing with cancer policymakers.

Registration. The trial is registered with ISRCTN: (trial no: ISRCTN22560297).

Word Count: 5665

Key words: Early cancer diagnosis, randomised controlled trial, clinical risk-assessment tools, General Practice

For peer review only - http://bmjopen.bmj.com/site/about/guidelines.xhtml

Article summary

Strengths and limitations of this study

- Improvements in primary care are seen as a key for improving early cancer diagnosis in the UK, and this trial is targeting that part of the diagnostic pathway.
- This is a large, definitive trial, powered to identify a clinically important difference in cancer stage at diagnosis.
- The trial is designed to minimise impact on participating practices with outcome data being obtained from routinely collected National Health Service data.
- One limitation is that the UK's national imperative to improve cancer diagnosis after the COVID pandemic may mean use of other interventions (or eRATs themselves) are encouraged by policymakers, reducing the validity and reliability of the trial.

For peer review only

Introduction

An estimated 10,000 UK cancer deaths each year would not occur if the UK matched the outcomes of other European countries.(1) Much of the difference is attributed to diagnostic delay.(2) The NHS Long Term plan, published in January 2019, specifically targets an increase in the percentage of cancer patients whose cancer is stage 1 or 2 (thus potentially curable) at diagnosis to rise from the current 54% to 75% by 2028.(3) Diagnosis of cancer may occur by several routes, but the main ones are population screening, and diagnosis after symptoms have occurred. Although screening for cancer is effective for colorectal, breast, lung and cervical cancers,(4-6) less than 10% of the total new UK cancers are identified by this route. Most of the remainder are diagnosed after presenting with symptoms, usually to primary care. Of patients with cancer, just under 20% present with an emergency complication of their cancer; however, many of these patients have previously reported symptoms attributable to their cancer to primary care, but this presentation did not lead to a diagnosis of cancer.(7) Within general practice, many studies have aimed at identifying the symptoms of possible cancer and quantifying their predictive value.(8) One main output has been Risk Assessment Tools (generally abbreviated to RATs); these give precise estimates of the chance of an underlying cancer as a percentage figure. RATs provide precise estimates for single symptoms (e.g. the risk of cancer of the lung for a person aged 40 years or more with haemoptysis is 2.4%), as pairs of symptoms (haemoptysis accompanied by loss of weight is 9.2%) or as repeated symptoms (a re-attendance with haemoptysis is 17%).(9) RATs are published for the 18 most common adult cancers, accounting for nearly 90% of the total cancer burden. These publications have been highly influential: in particular, they strongly contributed to the National Institute of Healthcare Excellence (NICE) guideline, Suspected cancer: recognition and referral [NG12], which guides symptomatic diagnosis of cancer in the UK.(10)

For peer review only - http://bmjopen.bmj.com/site/about/guidelines.xhtml

The initial RATs, of paper, mouse mat, calendar, or web-based forms, increased cancer diagnostic activity,(11) though impacts on hard outcomes such as stage at diagnosis or cancer survival were unknown. Electronic RATs (eRATs) for seven major cancers (lung, colorectal, pancreas, oesophago-gastric, bladder, kidney and ovary) have been developed for the two largest UK primary care electronic healthcare record systems, SystmOne and EMIS, used in around 80% of English practices. The software performs daily calculations of individual cancer risk in patients aged 40 and over, using coded symptoms and laboratory results in the patient's record over the past year, and prompts the general practitioner (GP) when the risk of one or more of these cancers is equal to or above 2%. Some form of electronic clinical decision support for cancer diagnosis has been downloaded by practices and used by at least one practice member in approximately 12% of English practices.(12). Two systematic reviews recently concluded that more research evidence was needed for impact on time to diagnosis and treatment, stage at diagnosis, and health outcomes, as well as research to understand how tools are used in GP consultations.(13) A feasibility trial of the oesophago-gastric eRAT published after these systematic reviews reported installation and regulatory problems that severely restricted usage,(14) and a vignette study of the colorectal RAT suggested it changed the GP's inclination to refer in 26% of usages.(15)

One crucial aspect of eRAT research relates to cost-effectiveness: annual NHS spending on cancer diagnosis is approximately £1bn.(16) Observational data showed increased use of the urgent cancer referral system to improve survival,(17) but there is insufficient data to inform a cost-effectiveness analysis of the subject.(13)

Objectives

The overarching aim of the trial is to assess the clinical and cost-effectiveness of using eRATs for six cancer sites – colorectal, lung, bladder, kidney, oesophago-gastric and ovarian cancers - compared with usual care for patients in general practice.

The primary objective is to compare the effects of using eRATs (vs usual care) on the percentage of patients with a newly diagnosed cancer at one of the six sites whose cancer is staged as being stage 1 or 2 (versus stage 3 or 4).

A secondary objective is to investigate differences in the stage at diagnosis of a further six cancers without eRATs (combined): breast, melanoma, prostate, Non-Hodgkin lymphoma, larynx and uterus. This is to investigate the possibility of an effect whereby eRATs are associated with increased diagnostic activity beyond the eRAT cancers. We will also investigate differences in: the number of

For peer review only - http://bmjopen.bmj.com/site/about/guidelines.xhtml

patients diagnosed with the six eRAT cancers combined, and the total number of cancers (excluding non-melanoma skin cancer) diagnosed, use of the 2-week wait referral system (the main pathway for urgent investigation of possible cancer in England) or equivalent for the six eRAT cancers combined, and across all cancers; the routes to diagnosis for each of the six eRAT cancers,(18) and for the six comparator non-eRAT cancers; the proportion of patients on a 2-week wait pathway receiving a diagnosis of cancer; whether a patient on a 2-week wait pathway has a diagnosis of cancer established (or refuted) within 28 days; 30-day and 1-year survival for those with cancer; the rate of cancer investigations, namely colonoscopies, sigmoidoscopies, upper gastro-intestinal endoscopies, chest x-rays, abdominal ultrasounds, and abdominal CT scans. We will also conduct parallel cost-effectiveness analyses, service delivery modelling and a process evaluation.

Methods and analysis

Design and setting

The study is a pragmatic cluster randomised-controlled trial in England, in primary care medical practices using one of the two (SystmOne or EMIS) electronic record keeping systems. The clusters are practices, a term which includes single practices, and small groups of practices agglomerated administratively to single entities. These will be randomised 1:1 to receive either the intervention (access to the suite of eRATs) or usual care. It is unrealistic to offer eRATs to individual GPs, as there would be considerable contamination within any practice. Nevertheless, for a practice to be eligible to take part, we ask at least 50% of GPs in that practice to agree to use the eRATs. Although the intervention is at the practice level, some process and resource use measures and all main trial primary and secondary outcomes relate to individual patients.

Intervention

The eRATs

The eRATs have been developed by a specialist IT team, Informatica systems Ltd, in partnership with the cancer charity, Macmillan. The risk estimates in the eRATs are from the original research papers for each cancer site. (9, 19-24) Practices will access the software via a new cloud-based system called Skyline, specifically designed to facilitate efficient integration into GP clinical systems. CA marking of the Skyline version of eRATs was obtained in September 2021.

The eRATs have multiple functions. The first is the *'prompt'*. This collates relevant coded symptoms and blood tests in the patient's medical record from the previous 12 months, which are then assessed for the possibility of cancer, generating a risk score equivalent to the positive predictive value of the cancer features for each cancer. A prompt (pop-up), displaying the risk score(s), appears on screen when a registered user opens a patient's medical records and indicates that patient has a risk of 2%

For peer review only - http://bmjopen.bmj.com/site/about/guidelines.xhtml

For peer review only

or higher for at least one of the studied cancers. A second function is the *'symptom checker'*, allowing the clinician to add the patient's symptoms to the eRAT checklist, which automatically recalculates the risk of any of the six cancers. On reviewing the risk score from the prompt and/or symptom checker, the clinician then decides the best course of management, which may be: (i) clinical review in primary care; (ii) ordering of test/investigations; or (iii) referral into secondary care. Embedded within all eRATS are links to authoritative guidance regarding the early diagnosis of cancer, NICE NG12,(25), Macmillan's abbreviated NICE guidance,(26) and Cancer Research UK guidance. (27) These sources of information are added to assist management of the patient, but the decision whether – or not – to investigate is for the clinician and patient. Some EMIS practices also have access to the QCancer risk tool, (28) albeit embedded in a dormant state within the practice IT and record system, and requiring manual activation prior to operation. All practices will be asked not to use it during the trial.

Justification of cancer sites

RATs are available for 18 adult cancers, each varying in their incidence, ease of diagnosis, amenability to treatment and proportion presenting as an emergency. We elected to study cancer sites a) which were in the top 15 cancers by incidence; b) for which curative treatment is reasonably possible in symptomatic patients;(29) and c) with a significant percentage of patients presenting as an emergency.(30). Using these criteria, six cancer sites were selected, amounting to approximately half of all incident cancers. The remaining nine cancers were considered as comparators to examine for any practice level effect of increased cancer diagnostic activity. Three cancers, brain, pancreas and leukaemia, were removed for clinical and practical reasons: no eRAT is available for brain or leukaemia; in both brain and pancreas, symptomatic diagnosis is considered to have a very small likelihood of improving survival,(29) and in leukaemia, a full blood count (easily available in primary care) will usually establish the diagnosis, making an eRAT unlikely to expedite the diagnosis.(31)

Training practices in using eRATs

Training in the use of the eRATs uses short, pre-recorded videos available online co-ordinated by a practice 'research champion'. These show GPs how to use the prompt and symptom checker functions.

Duration of intervention

Practice recruitment started in August 2019 and is expected to finish at the end of March 2022, including the installation of the eRATs software. The trial was paused for 6 months in March 2020 due

For peer review only - http://bmjopen.bmj.com/site/about/guidelines.xhtml

to Covid-19. The formal start of the intervention window will be 01/04/2022 (although some practices may have delayed installation) and will close for all intervention practices on 31/3/2024.

Usual care

Patients presenting to the control practices will experience the GP's usual diagnostic approach. GPs in control practices will have no specific on-screen prompt, though they may have access to hard-copy (e.g. paper or mouse mat) versions of the RATs, or to other cancer tools such as those supporting structured follow-up of symptomatic patients not selected for initial investigation. For EMIS practices with QCancer dormant in the system, control practices are expected to leave it dormant. We will document control practice use of RATs, other decision support tools, and access to and use of eRATs via interim and exit questionnaires completed within the first 12 months of a practice commencing the intervention and at the end of the trial. In line with intervention practices, trial time will formally begin for control practices on 01/04/2022 and end on 31/03/2024.

Data collection window

Outcome data for all practices will be obtained for the 2-year period from 01/06/2022 to 29/05/2024. This data collection window is lagged behind the trial time window (01/04/2022 to 31/03/2024) in order to: a) provide some time for practices to become accustomed to how the intervention functions prior to data collection, and b) to have a 2-month window following the end of the intervention window in order to allow cancers to be diagnosed in patients seen towards the end of that window.

Sample size

There are around 130,000 new diagnoses of the six included cancers in the UK annually.(32) As each of our six cancer sites has different proportions diagnosed at an early stage, the sample size calculation is based on a relative improvement in staging, using an odds ratio of 0.8 for a cancer being diagnosed at Stage 3/4 in the intervention arm compared with the control arm. This difference is quite large and equates to an absolute reduction of 4.8% in the intervention arm as an incidence-weighted figure across the six cancers. A much smaller improvement would still be clinically valuable but would necessitate an impossibly large trial.

For the inflation factor we have used an intra-cluster correlation coefficient based on our previous work, of 0.05.(33) An average cluster size of 23 patients with a diagnosed cancer with recorded stage during 2-year follow-up is expected, with a coefficient of variation for cluster size of 0.7, giving a design effect of 2.66. For an individually randomised trial with 90% power and an alpha threshold of 0.05, the sample size would be 2,049 patients per arm. Adding in the design effect, this becomes 5,497 patients, requiring 239 practices per arm, and 478 practices in total. Due to changes in practice structure (such

For peer review only - http://bmjopen.bmj.com/site/about/guidelines.xhtml

as practice mergers, closures or divisions), we anticipate the loss of up to 10% of recruited practices over the course of the trial; to account for this we will recruit a target of 530 practices overall, expecting 12,190 patients to be diagnosed with cancer in total.

Practice recruitment

A total of 530 primary care practices across England will be recruited, supported by the NIHR Clinical Research Network (CRN) and strategic media releases to raise awareness of the trial. Practices that are proposing a split or a merger are not eligible for the trial, as the practices before or after the change may have been allocated to different arms in the trial. A method for identifying and managing unanticipated splits or mergers during the active phase of the trial is shown in Appendix A.

Patients are not being recruited into this trial - patient consent is not being sought for the use of the eRATs during the consultation. This is because ERATs are essentially an extension and enhancement of existing diagnostic tools already available to the GP to support their clinical decision making. Other randomised controlled trials of interventions in primary care have taken this approach,(34) including the feasibility trial of the oesophago-gastric eRAT.(14, 35, 36) To promote patient awareness of the practice's participation in the ERICA trial, including requesting practices to add it to their websites and any social media feed. A selection of patients will be recruited to the nested process evaluation and health economics studies (see below and Appendices B and D).

Randomisation

Practices will be randomised using a 1:1 ratio into one of two trial arms: usual diagnostic care (control) and usual diagnostic practice plus access to the suite of eRATs, as the intervention. Randomisation will be computer-generated and web-based, conducted by an independent member of staff at the Exeter Clinical Trials Unit (ExeCTU), overseen by the CTU statistician (not the trial statistician). To promote balance between the trial arms in practices' use of the 2-week wait system, and therefore propensity to refer to secondary care, we will minimise randomisation by age-sex standardised 2-week wait referral ratio (the best available proxy) in national tertiles. We will use simple randomisation to allocate the first 50 practices (~10% of the total target), and then apply minimisation by 2-week wait referral ratio tertile, taking into account the previous allocations to inform the minimisation algorithm. To promote allocation concealment, all allocations using the minimisation algorithm will retain a stochastic element.

The data analysis will be carried out by the trial statistician and health-economist, blinded to treatment allocation and all primary outcome data are objective assessments of clinical outcome. Staging (the primary outcome) will be performed by pathologists unaware of trial participation or

For peer review only - http://bmjopen.bmj.com/site/about/guidelines.xhtml

allocation. However, given the nature of the intervention, it is not possible to blind GPs or the GP practice to treatment allocation.

Outcome measures

Primary outcome

Outcome measures will be captured at patient-level, using data routinely collected by the National Cancer Registration and Analysis Service (NCRAS). The primary outcome is whether a patient is diagnosed at stage 1 or 2 (early) or stage 3 or 4 (advanced). This division of staging is commonly used and is a targeted metric in the 2019 NHS Long Term Plan - for stage 1 and 2 cancers (for all staged cancers other than non-melanoma skin cancer) at diagnosis to comprise 75% of the total by 2028. The current UK overall incidence-weighted percentage of early stage at diagnosis was 55% in 2018, though for the six eRAT cancers, it is 35%.(37)

Secondary outcomes

A range of secondary outcomes will be examined:

1. 1. The binary stage at diagnosis of a further six cancers without eRATs will be identified from NCRAS, and compared between intervention and control practices. This is to investigate the possibility of a 'spill-over' effect whereby eRATs are associated with increased diagnostic activity beyond the eRAT cancers.
2. The practice's number of patients diagnosed with the six eRAT cancers combined, and the total number of cancer cases, from NCRAS.
3. The number of patients investigated or referred under the 2-week wait system for the six eRAT cancers combined, and in total, from Cancer Waiting Times data.
4. Route to diagnosis from the Routes to Diagnosis Dataset,(18) which uses Hospital Episode Statistics data. This will be categorised into four possible routes: emergency attendance, 2-week wait referral, GP referral, and "other". We will collect this information for each of the six eRAT cancers, and for the six comparator non-eRAT cancers.
5. 2-week wait performance measures, from Cancer Waiting Times data, for the six eRAT cancers combined, and for all cancer referrals:

5.1 Whether a patient on a 2-week wait pathway received a diagnosis of cancer, expressed as– the proportion of patients who received a cancer diagnosis, also known as the conversion rate.

5.2 The duration between 2-week wait referral and diagnosis of cancer, in particular diagnosis within 28 days, the Faster Diagnosis Standard (introduced in 2022).

For peer review only - http://bmjopen.bmj.com/site/about/guidelines.xhtml

5.3 Detection rate – the proportion of a practice's cancers which are identified via the 2-week wait pathway.

6. Survival measures: 30-day; 1-year (identified from NCRAS). 5-year survival will also be reported, but the main trial will report on 30 day and 1-year, with 5-year data being a subsidiary report.

7. Adverse events (using data from the Diagnostic Imaging Dataset): these are expected to be few, and largely related to complications from hospital investigation such as colonoscopy. There is no mechanism for adverse events to be collected using routine data. We will, however, estimate any change in the expected number of adverse events from imaging investigations (colonoscopies, sigmoidoscopies, upper gastro-intestinal endoscopies, chest x-rays, abdominal ultrasounds, and abdominal CT scans) through investigating any change in the rate of these investigations in intervention practices relative to control practices (see data analysis section). Potential adverse psychological consequences of being labelled with 'possible cancer' will be further explored in the process evaluation.

Data collection

All primary and secondary outcome measures are available from NCRAS, DID and publicly available practice level data, including Cancer Waiting Times data. We will be using depersonalised (pseudo-anonymised) data. The Public Health England Office for Data Release (ODR) guidelines indicated that no legal gateway (e.g., section 251 approval) will be necessary to obtain these data.

Data analysis

All analyses will follow CONSORT guidelines for cluster-randomised and pragmatic trials. The primary analysis, exploring the proportion of cancer patients with early stage at diagnosis, will use mixed-effects logistic regression with a random intercept for practice to accommodate the hierarchical nature of the data (i.e. random allocation by practice, with participants nested within practice). This regression will include trial-arm at practice-level, and will adjust for patient-level covariates known to be associated with stage (age, sex, quintile of the income domain from the Index of Multiple Deprivation (IMD), and cancer site),(38) and the practice-level minimisation variable (national tertile of age-sex standardised two-week wait referral ratio). We will further adjust the model at the practice-level for list size, clinical IT system used, and Care Quality Commission (CQC) overall rating, should these variables be associated with stage in preliminary analyses (even if not unbalanced with respect to trial allocation). Trial arm and covariates will all be entered as fixed effects. The degree of change

For peer review only - http://bmjopen.bmj.com/site/about/guidelines.xhtml

in the percentage of patients diagnosed at a late stage in intervention practices will be investigated by exploring the marginal distributions of trial arm on the probabilities predicted by these models.

For the secondary outcome of the stage at diagnosis of six cancers without eRATs, we will repeat the above model including data on the six non-eRAT cancers as well as the six eRAT cancers. This model will use all the variables described above, plus an indicator variable for whether the cancer site has an eRAT and an interaction term between this variable and trial arm. From this model, we will obtain odds ratios (with 95% CIs) for: (i) the “spill over” effect of having the intervention on cancer sites not included in the intervention, and (ii) for the relative effect of the intervention on stage for included cancer sites compared with those not included in the intervention.

Mixed-effects logistic regression models with a random intercept for practice will also be fitted for the other secondary binary outcomes; route to diagnosis, conversion rate, and timeliness. These models will include trial arm as a practice-level effect, and will adjust at the patient-level for age, sex, and quintile of the Index of Multiple Deprivation (IMD) income domain, and at the practice-level for the minimisation variable (national tertile of age-sex standardised two-week wait referral ratio). These analyses will also adjust at the patient-level for cancer site (routes to diagnosis analyses) or for referral type (2-week wait analyses) as appropriate. The models will be further adjusted as in the main outcome variable analysis.

Time-to-event secondary outcomes (length of waiting time, survival) will be analysed using mixed-effects parametric survival models with a random intercept for practice, and all other variables added as fixed effects. These models will include trial-arm as a practice-level effect, and will adjust for the same patient-level factors as described above (waiting times adjusted for referral pathway rather than cancer site as above), and the practice-level minimisation variable (national tertile of age-sex standardised 2-week wait referral ratio). The models will also use the same adjustment as the primary outcome measure. An appropriate distribution to model the baseline hazard will be utilised, as determined by a comparison of the Akaike Information Criteria under different distributions.(39)

For rate outcomes (number of 2-week wait referrals, cancers, and imaging investigations), we will analyse the rates per 100,000 registered patients per year by age-sex strata using mixed-effects Poisson regression models including a random intercept for practice. These models will include trial-arm as a predictor and will adjust for the age and sex of the strata, and at the practice-level for the minimisation variable (2-week wait referral ratio) and deprivation (quintile of IMD overall score). The models will be further adjusted at the practice-level for list size, clinical IT system used, CQC overall rating, and for the age and sex case-mix of practices should these covariates be found to be associated

For peer review only - http://bmjopen.bmj.com/site/about/guidelines.xhtml

with the outcome (even if not unbalanced with respect to allocation). Case-mix will be incorporated by including variables for counts of practice populations in different age-sex strata (5-year age groups by sex, excluding one age group-sex stratum that can be determined once all others are known).

All the above analyses will combine data for the six eRAT cancers for each model. For outcomes related to two-week wait referrals, data will be combined for all referral pathways relevant to the six eRAT cancers. To investigate whether the eRATs produce a "spill-over" effect, whereby diagnostic activity is increased for other cancers, we will repeat all analyses using data for the six non-eRAT cancers combined for each model. Investigation of a spill-over effect for 2-week wait referral outcomes will use data for all referral pathways combined.

Additional sensitivity analyses will be conducted for the primary outcome in order to explore moderation arising from practice-level characteristics, using interaction terms. Although the trial has not been powered to detect low to moderate subgroup differences, large interaction effects that differ with respect to the direction of effect across subgroups are of interest. The potential impact of missing staging data on the primary outcome will also be explored through use of multiple imputation methods making use of auxiliary variables such as survival time, morphology and grade to improve the Missing At Random (MAR) assumption in line with previous work).(40)

Data management

Cancer registry data (NCRAS) will be managed and prepared by the registry themselves and securely, electronically transferred to the study team. There will be no patient identifiable data within these datasets. Data from NCRAS will be stored on the Secure Data Resource Hub at the University of Exeter (which meets requirements for secure storage of sensitive data) and linked to existing practice data held within ExeCTU's REDCap database. The data will be stored and retained in accordance with registry policies.

The nested studies rely on identifying patients from in-practice usage reports. These reports contain depersonalised (pseudo-anonymised) data. The practice will send a copy to the trial team with the original practice ID number removed. The local at practice reports will be securely and electronically transferred to a secure Exeter CTU computer.

In the recruitment of patients (and NHS staff) for interviews, questionnaires, or permission for access to medical notes, participant details will be passed securely between NHS services and the research team. All participants agreeing to interview, to complete a questionnaire, and/or medical notes review, and all GPs agreeing to interview will be allocated a unique study ID, and the information linking their ID to their personal details will be kept securely at the University of Exeter. All other

For peer review only - http://bmjopen.bmj.com/site/about/guidelines.xhtml

participant-related paper records will be anonymised and stored separately from the personal information. The electronic database for the trial will be stored on the secure servers of the University of Exeter with password-controlled access provided for the research team by ExeCTU. Single data entry with extensive in-built validity checks will be used to reduce the risk of transcription errors.

Audio recordings will be digitised, encrypted and stored on the University's secure server. Audio recordings will be retained until after anonymised transcripts have been finalised and analysed. At this stage they will be securely and permanently deleted. Access to personal data will be restricted to the research team. Names and participant details will not be passed to any third parties and no named individuals will be included in the outputs. All participants (patients, NHS staff) will be asked for their consent for the study team to retain interview transcripts for the purposes of future research by those involved directly in the study team or to be used for educational purposes.

Informatica Systems Ltd has developed a separate agreement ('Data processing deed') for intervention practices which will be used between the GP practices and Informatica Systems Ltd. The deed was necessary because the development of Skyline has impacted on the processing arrangements for the eRATs software that is used. The ERICA research study will still use the Organisation Information Document which outlines the research team's data processing requirements, to be signed between the practice and Sponsor.

All study data will be kept for 10 years (unless data registry policy requires otherwise) under secure conditions on University of Exeter secure servers. Data will also be subject to standard secure storage and usage policies.

Trial monitoring and management

Trial Sponsor and Funders

The University of Exeter is the trial sponsor. The trial funders are providing finance to run the trial. None of the funders or sponsor will be involved in the design or day-to-day conduct of the trial, analysis of data, or interpretation of findings.

Trial Steering Committee (with Data Monitoring Committee responsibilities)

The responsibilities of the Trial Steering Committee (TSC) will be to review the main study protocol and any amendments, monitor and supervise the trial towards its interim and overall objectives, review relevant information from other sources, and to help resolve problems brought by the Trial

For peer review only - http://bmjopen.bmj.com/site/about/guidelines.xhtml

Management group (TMG). The TSC will therefore provide overall independent supervision for ERICA on behalf of the funders and the Sponsor. Meetings will be held at regular intervals determined by need and not less than twice a year. Routine business will be conducted by telephone, videoconference, and email. The TSC will also operate as a Data Monitoring Committee with responsibility to monitor the overall conduct of the trial. There will be a time lag between practices 'entering the trial' and data availability from cancer registries. The time lag will be such that data will only be available once practices have completed data collection. Therefore, interim analyses to assess whether the trial was effective, and to support a decision whether to stop the trial early, would be unnecessary as data collection (and practice participation) would have already ceased.

Trial Management Group

A TMG has been established and includes those responsible for the day-to-day management of the trial and those supporting the delivery of the trial and associated stakeholders, including representatives of the Local Clinical Research Networks (LCRN) and Macmillan. The group will monitor all aspects of the conduct and progress of the trial, ensure that the protocol is adhered to and take appropriate action to safeguard participants and the quality of the trial itself. The group will meet regularly (monthly in the first instance, until recruitment has completed) in person and/or by phone or over the internet (via MS Teams).

Core Study Team

The core study team (Chief investigator, Trial Manager (TM)) will meet weekly during the study. Day-to-day running of the trial will be the responsibility of the TM. The TM will have access to the ExeCTU suite of standard operating procedures (SOPs) and will ensure that the trial is run in compliance with all relevant SOPs (e.g., assessment, processes and reporting, data management, study staff health and safety).

Nested Studies

Health Economics

We will estimate the cost and cost-effectiveness of the eRATs versus usual diagnostic practice using the primary perspective of the NHS and Personal Social Services (i.e. third-party payer). We will estimate the cost-effectiveness of the intervention based upon the primary outcome and secondary survival outcomes (30-day and 1-year; 5-year survival will be a subsidiary report) for the six cancer sites with eRATs and report the results using the latest guidelines.(41) For three cancer sites we will use decision analytic models to combine data from the within-trial analysis of ERICA intervention on costs and benefits, with longer estimates derived from the evidence synthesis of the costs and benefits

For peer review only - http://bmjopen.bmj.com/site/about/guidelines.xhtml

of stage of diagnosis and disease progression to estimate the cost per Quality Adjusted Life Year (QALY) over the longer term.(42) For fuller details see Appendix B.

Service Delivery Modelling

This will investigate the key factors central to the (re) organisation of NHS diagnostic services for cancer referrals. We will use a range of methods, both quantitative and qualitative, to analyse service delivery alternatives. Specifically, we will aim to use modelling approaches to explore the likely implications of different scenarios across dimensions of performance, outcomes and costs. Fuller details are in Appendix C.

Process Evaluation

The process evaluation work aims to identify and investigate the contextual factors that impact upon the effectiveness of the eRATs with a particular focus on intervention fidelity and GP engagement. The impact of the eRATs on the patients' experience of their GP consultation and their experiences of subsequent care will also be explored. Fuller details are in Appendix D.

GP Workload

This nested study aims to explore, in terms of consultation time, the impact of GPs using eRATs on GP workload and patient 'flow' through consulting sessions. It will also explore workload in the week following the index consultation in which an eRAT was activated. Fuller details are in Appendix E.

Patient and Public Involvement and Engagement

Our Patient and Public Involvement and Engagement (PPIE) group, including cancer survivors, have been consulted widely during the development of this study. The PPIE group have reviewed and commented on the protocol and supported the development of all patient-facing materials including information sheets and study lay summaries. One experienced PPIE representative sits on the TMG and another is on the TSC. A total of seven people have joined our PPIE group for this study and will contribute by reviewing study materials and documentation, commenting upon and proof reading reports and contributing to dissemination activities. This group will be supported in their work by the South West Peninsula Applied Research Collaboration (PenARC) PPIE team, for example by attending workshops on critical appraisal skills. All PPIE representatives will be recompensed for their time given to the study.

Dissemination policy

For peer review only - http://bmjopen.bmj.com/site/about/guidelines.xhtml

A trial publication policy will be developed which outlines the plan for dissemination and will be in accordance with the International Committee of Medical Journal Editors. The results of the trial will be reported first to study collaborators and to the funder. The main report will be drafted by the TMG and circulated to all collaborators and the TSC for comment.

Access to the final trial datasets will be made publicly available unless contractual agreements between data providers limit such access.

Ethical review

The trial has received favourable Ethical review from London City & East Research Ethics committee, reference number 19/LO/0615, with five amendments between then and 2022, relating to two main areas: the delays caused by the COVID-19 pandemic, with its recruitment moratorium, and an alteration in the mechanism by which the eRATs software were delivered. Current version – V 5.0, 9th May 2022.

Author contributions: The protocol was written by RC, LM, SD, GA, AS, EF, and MP under the overall editorial control of WH. All authors have contributed to revision of the protocol.

Funding statement: This research is funded by a philanthropic donation by The Dennis and Mireille Gillings Foundation, The University of Exeter, and Cancer Research UK, plus support from Macmillan in provision of staff time. The trial is registered with ISRCTN: (trial no: ISRCTN22560297) and on the CRUK trial registry (CRUK database no: 16163).

Acknowledgements: We would like to thank the NIHR Clinical Research Network for their support with recruitment, Macmillan for their contributions to the early eRAT work and ongoing support with practice recruitment and pilot testing. SD's time is partially supported by the National Institute of Health Research Applied Research Collaboration (ARC) South-West Peninsula.

Disclaimer: The views expressed are those of the authors and not necessarily those of the NHS, the NIHR or Department of Health.

Competing interests statement: WH has intellectual property rights to the original RATs, though has never sought to commercialize these. All other authors: no competing interests to declare.

For peer review only - http://bmjopen.bmj.com/site/about/guidelines.xhtml

References

1. Richards MA. The size of the prize for earlier diagnosis of cancer in England. Br J Cancer. 2009;101.
2. Richards M, Thorlby R, Fisher R, Turton C. Unfinished business: An assessment of the national approach to improving cancer services in England 1995–2015. London; 2018.
3. NHS England. (2019) The NHS Long Term Plan [Available from: https://www.longtermplan.nhs.uk/.
4. de Koning HJ, van der Aalst CM, de Jong PA, Scholten ET, Nackaerts K, Heuvelmans MA, et al. Reduced Lung-Cancer Mortality with Volume CT Screening in a Randomized Trial. New England Journal of Medicine. 2020;382(6):503-13.
5. Richards M. THE INDEPENDENT REVIEW OF ADULT SCREENING PROGRAMMES. NHS England; 2019. Contract No.: 01089.
6. Landy R, Pesola F, Castanon A, Sasieni P. Impact of cervical screening on cervical cancer mortality: estimation using stage-specific results from a nested case-control study. Br J Cancer. 2016;115(9):1140-6.
7. Abel GA, Mendonca SC, McPhail S, Zhou Y, Elliss-Brookes L, Lyratzopoulos G. Emergency diagnosis of cancer and previous general practice consultations: insights from linked patient survey data. British Journal of General Practice. 2017.
8. Hamilton W, Walter FM, Rubin G, Neal RD. Improving early diagnosis of symptomatic cancer. Nature Reviews Clinical Oncology. 2016;13:740.
9. Hamilton W, Peters TJ, Round A, Sharp D. What are the clinical features of lung cancer before the diagnosis is made? A population based case-control study. Thorax. 2005;60(12):1059-65.
10. NICE. Suspected cancer: recognition and referral [NG12] 2015 [Available from: https://www.nice.org.uk/guidance/ng12.
11. Hamilton W, Green T, Martins T, Elliott K, Rubin G, Macleod U. Evaluation of risk assessment tools for suspected cancer in general practice: a cohort study. British Journal of General Practice. 2013;63(606):e30-e6.
12. Price S, Spencer A, Medina-Lara A, Hamilton W. Availability and use of cancer decision-support tools: a cross-sectional survey of UK primary care. British Journal of General Practice. 2019;69(684):e437.
13. Medina-Lara A, Grigore B, Lewis R, Peters J, Price S, Landa P, et al. Cancer diagnostic tools to aid decision-making in primary care: mixed-methods systematic reviews and cost-effectiveness analysis. Health technology assessment (Winchester, England). 2020;24:66.
14. Rubin G, Walter FM, Emery J, Hamilton W, Hoare Z, Howse J, et al. Electronic clinical decision support tool for assessing stomach symptoms in primary care (ECASS): a feasibility study. BMJ Open. 2021;11(3):e041795.
15. Kostopoulou O, Arora K, Pálfi B. Using cancer risk algorithms to improve risk estimates and referral decisions. Communications Medicine. 2022;2(1):2.
16. Hamilton W. Diagnosing symptomatic cancer in the NHS. BMJ : British Medical Journal. 2015;351:h5311.
17. Møller H, Gildea C, Meechan D, Rubin G, Round T, Vedsted P. Use of the English urgent referral pathway for suspected cancer and mortality in patients with cancer: cohort study. BMJ. 2015;351.
18. Elliss-Brookes L, McPhail S, Ives A, Greenslade M, Shelton J, Hiom S. Routes to diagnosis for cancer - determining the patient journey using multiple routine data sets. Br J Cancer. 2012;107.
19. Hamilton W. The CAPER studies: five case-control studies aimed at identifying and quantifying the risk of cancer in symptomatic primary care patients. Br J Cancer. 2009;101 Suppl 2:S80-6.
20. Hamilton W, Peters TJ, Bankhead C, Sharp D. Risk of ovarian cancer in women with symptoms in primary care: population based case-control study. BMJ. 2009;339:b2998.

For peer review only - http://bmjopen.bmj.com/site/about/guidelines.xhtml

21. Hamilton W, Round A, Sharp D, Peters TJ. Clinical features of colorectal cancer before diagnosis: a population-based case-control study. Br J Cancer. 2005;93(4):399-405.
22. Shephard E, Neal R, Rose P, Walter F, Hamilton WT. Clinical features of kidney cancer in primary care: a case-control study using primary care records. The British journal of general practice : the journal of the Royal College of General Practitioners. 2013;63(609):e250-5.
23. Shephard EA, Stapley S, Neal RD, Rose P, Walter FM, Hamilton WT. Clinical features of bladder cancer in primary care. The British journal of general practice : the journal of the Royal College of General Practitioners. 2012;62(602):e598-604.
24. Stapley S, Peters TJ, Neal RD, Rose PW, Walter FM, Hamilton W. The risk of oesophago-gastric cancer in symptomatic patients in primary care: a large case-control study using electronic records. Br J Cancer. 2013;108(1):25-31.
25. NICE. Suspected cancer: recognition and referral [NG12]2015. Available from: http://www.nice.org.uk/guidance/NG12.
26. Macmillan. (2015) Rapid referral guidelines [available at: https://www.macmillan.org.uk/_images/rapid-referral-toolkit-desktop_tcm9-291864.pdf].
27. Cancer Research UK. (2017) Cancer Stats 2017 [Available from: http://www.cancerresearchuk.org/health-professional/cancer-statistics].
28. Collins GS, Altman DG. Identifying patients with undetected gastro-oesophageal cancer in primary care: External validation of QCancer(R) (Gastro-Oesophageal). European journal of cancer (Oxford, England : 1990). 2013;49(5):1040-8.
29. Hamilton W, Stapley S, Campbell C, Lyratzopoulos G, Rubin G, Neal R. For which cancers might patients benefit most from expedited symptomatic diagnosis? Construction of a ranking order by a modified Delphi technique. BMC Cancer. 2015;15:820.
30. McPhail S, Johnson S, Greenberg D, Peake M, Rous B. Stage at diagnosis and early mortality from cancer in England. Br J Cancer. 2015;112(s1):S108-S15.
31. Shephard EA, Neal RD, Rose PW, Walter FM, Hamilton W. Symptoms of adult chronic and acute leukaemia before diagnosis: large primary care case-control studies using electronic records. British Journal of General Practice. 2016;66(644):e182-e8.
32. UK CR. Statistics by Cancer Type 2022 [Available from: https://www.cancerresearchuk.org/health-professional/cancer-statistics/statistics-by-cancer-type.
33. Barclay ME, Lyratzopoulos G, Greenberg DC, Abel GA. Missing data and chance variation in public reporting of cancer stage at diagnosis: Cross-sectional analysis of population-based data in England. Cancer Epidemiology. 2018;52:28-42.
34. Campbell JL, Fletcher E, Britten N, Green C, Holt TA, Lattimer V, et al. Telephone triage for management of same-day consultation requests in general practice (the ESTEEM trial): a cluster-randomised controlled trial and cost-consequence analysis. The Lancet. 2014;384(9957):1859-68.
35. Downs M, Turner S, Bryans M, Wilcock J, Keady J, Levin E, et al. Effectiveness of educational interventions in improving detection and management of dementia in primary care: cluster randomised controlled study. BMJ. 2006;332(7543):692.
36. Flottorp S, Oxman AD, Håvelsrud K, Treweek S, Herrin J. Cluster randomised controlled trial of tailored interventions to improve the management of urinary tract infections in women and sore throat. Bmj. 2002;325(7360):367.
37. National Disease Registration Service:. Case-mix adjusted percentage of cancers diagnosed at stages 1 and 2 in England, by Clinical Commissioning Group2020 25 Jan 2022. Available from: https://www.gov.uk/government/statistics/case-mix-adjusted-percentage-cancers-diagnosed-at-stages-1-and-2-by-ccg-in-england/national-disease-registration-service-case-mix-adjusted-percentage-of-cancers-diagnosed-at-stages-1-and-2-in-england-by-clinical-commissioning-group.
38. Lyratzopoulos G, Abel GA, Brown CH, Rous BA, Vernon SA, Roland M, et al. Socio-demographic inequalities in stage of cancer diagnosis: evidence from patients with female breast, lung, colon, rectal, prostate, renal, bladder, melanoma, ovarian and endometrial cancer. Annals of Oncology. 2012;24(3):843-50.
39. Bozdogan H. Model selection and Akaike's Information Criterion (AIC): The general theory and its analytical extensions. Psychometrika. 1987;52(3):345-70.

For peer review only - http://bmjopen.bmj.com/site/about/guidelines.xhtml

40. Lyratzopoulos G, Abel GA, Brown CH, Rous BA, Vernon SA, Roland M, et al. Socio-demographic inequalities in stage of cancer diagnosis: evidence from patients with female breast, lung, colon, rectal, prostate, renal, bladder, melanoma, ovarian and endometrial cancer. Ann Oncol. 2013;24(3):843-50.
41. Husereau D, Drummond M, Augustovski F, de Bekker-Grob E, Briggs AH, Carswell C, et al. Consolidated Health Economic Evaluation Reporting Standards 2022 (CHEERS 2022) statement: updated reporting guidance for health economic evaluations. BMJ. 2022;376:e067975.
42. Weinstein MC, O'Brien B, Hornberger J, Jackson J, Johannesson M, McCabe C, et al. Principles of Good Practice for Decision Analytic Modeling in Health-Care Evaluation: Report of the ISPOR Task Force on Good Research Practices—Modeling Studies. Value in Health. 2003;6(1):9-17.

For peer review only

A pragmatic cluster randomised controlled trial assessing the clinical effectiveness and cost-effectiveness of electronic risk-assessment for cancer for patients in general practice (ERICA): Appendices

For peer review only

Appendix A. Managing practice splits and mergers in analyses

Although we will exclude practices that report imminent restructuring during recruitment, there may be unforeseen mergers or splits of practices. Where mergers and splits are concerned, this could mean, for example, that some of our practices who were in the control arm may merge with an intervention practice. Similarly, a non-trial practice may become part of a trial practice (intervention or control). Changes in practice size have implications on the denominator – the number of patients that each practice is likely to be contributing to our sample – and is a particular issue for three of our secondary outcome measures based on rates (cancer diagnosis rate, two-week wait referral rate, and adverse event rate). Importantly, however, this issue is not a problem for our primary outcome of staging.

We define a split and mergers as follows: Split – Where a population of patients registered to a single practice with a single practice code become registered with two or more individual practices with different practice codes. The practice codes of the new practices may be new codes (i.e. did not exist prior to the split) or one may inherit the original practice code (although this is not a requirement). The change in registration of patients must occur to a substantial number of patients and not at their request. Merger – Where a population of patients registered to one or more practices with different practice codes become registered at a single individual practice with a single practice code. The practice code of the new practice may be a new code (i.e., did not exist prior to the split) or it may inherit one of the original practice codes. A federation is not a “merger” in these terms.

Excluding who restructure during the trial may unnecessarily reduce our power. Therefore, we will try and accommodate changes in status. The Table outlines our approach. The assumption is that the change takes place at time T. Any practice which splits goes from X to Y and Z, and mergers are Z plus Y becoming X. Intervention practices are I, and comparison practices C.

Practice size fluctuations will be monitored in real time. Practice size data are freely and publicly available from NHS Digital and are updated monthly. Each month during the data collection, the trial statistician will download the practice size data and inspect size for all the practices in the trial (the statistician will remain blinded to outcome allocation). If the practice size differs by more than 10% the statistician will alert the trial manager, who will contact the research champion in the relevant practice to explore the reasons for this practice size change. Reasons (e.g., mergers, splits) will be recorded.

For peer review only

Table: managing changes in practice size – mergers and splits

Split or merger	X pre change	Y pre change	Z pre change	X post change	Y post	Z post
**Split**	I				I	I
	C				C	C
We will allow the daughter practices to withdraw from the trial if they desire, which would mean we lose Y or Z (or both). If daughter practices decide to withdraw, we will include data up to time T plus 2 months to allow for average diagnostic time to cancer.						
**Merger**		I	I	I		
		I	C	I	There is likely to be wash over under these conditions, so the merged practice will be considered as I	
		I	Non-trial	I		
		C	Non-trial	C		
		C	C	C		

We will manage changes in practice size at the data analysis stage of the trial. Where changes in list size of more than 10% within a month are seen, data for that practice will not be included in the analysis of rate outcomes from one month prior to the drop. There are two exceptions to this; 1) splits where all the daughter practices remain in the trial and we continue to treat them as a single practice for rate analyses, 2) mergers where merged practices are in the same arm of the trial, and we will analyse them as a single practice from the start for rate analyses.

Appendix B

Health Economics

Intervention costings. The resources used in developing the training materials and videos (preparation and IT support) will be collected from the trial manager; nationally applicable unit costs will be applied. Estimates of the extent to which these videos are watched by practice staff will be based on information available from the website platform hosting the videos. Information on the resources use to install the eRATs onto the EMIS and SystmOne practice IT systems will be estimated in consultation with practice champions. These estimates will additionally aim to estimate: 1) the cost

For peer review only

of installation in the trial and 2) the anticipated cost of future installation should eRATS be implemented nationally.

Health related quality of life and resource use. The Health Economics analysis will draw on the estimated number of imaging investigations (colonoscopies, sigmoidoscopies, upper gastro-intestinal endoscopies, chest x-rays, abdominal ultrasounds, and abdominal CT scans) using data from the Diagnostic Imaging Dataset available in the main trial as well as estimates of GP workload from the process evaluation.

To investigate whether the eRATs intervention was associated with a change in health-related quality of life using the EQ5D-5L and to provide more detailed information on primary care services and tests used, we will sample patients in the intervention arm who had a consultation where an eRAT alert occurred, and patients in the control arm who had a consultation where an eRAT alert would have occurred. We will strategically target practices in both trial arms who have either high, medium, or low two-week wait referral rates, matching the minimisation criteria in the main trial. It is anticipated that 15-20 patients per practice over a 2-week period will have a consultation with an eRAT alert. All patients who have an eRAT alert will be invited to complete a baseline questionnaire and a 3 month follow-up Health Economics questionnaire, as will equivalent patients in the control arm. We anticipate that 40% of patients will accept, and of these there will be 20% who do not respond. With a conservative estimate of a cluster size of five patients responding to the questionnaire. Using an minimum clinically important difference of 0.1 for the EQ5D-5L (2) and a standard deviation of 0.23(3), with an inter-cluster correlation coefficient of 0.03 (4), and an estimated coefficient of variation of cluster size of 0.7, the sample size required to detect a between group difference with 90% power and alpha of 0.05 was 28 clusters (140 participants) per arm. Participants who agree to take part will receive the questionnaire as a hard copy, through the post, or electronically via email, depending on the participant's preference. Nationally applicable unit costs will be used for all community health and social care contacts (5) and secondary care services, tests and investigations will be costed using the National Schedule of Reference Costs 2016-2017 (6).

Decision Analytic Model

The modelling aims to predict the expected impact of a change in stage of diagnosis, and any resulting change in the distribution of cancer stage at diagnosis (intervention vs. control) over time, building on the published literature in this area.(9, 10) The decision analytic models will not need to separately model the diagnostic phase, and we will take the trial's primary outcomes, stage at diagnosis (Stage

For peer review only

1-4 separately and not collated into Stage 1-2 and Stage 3-4), to model the longer term effects on survival, QALYs and secondary care costs.

Scenario analysis will be used to examine the impact on the results of multiple parameters changing simultaneously (based on *a priori* judgement about the combination of parameters to include).(11) Probabilistic sensitivity analysis will be used to explore the proportion of results that are considered cost-effective in relation to a given cost-effectiveness threshold and these results will be illustrated graphically using a cost-effectiveness acceptability curve.(12)

The study will follow the CHEERs guidelines for reporting cost-effectiveness studies and models,(13) and will discount both costs and outcomes at 3.5% as recommended by the National Institute of Health and Care Excellence.(14) Sensitivity analyses will examine alternative assumptions about the missing data mechanisms.(15)

Service Evaluation

We will draw upon published systematic reviews of Quality of Life measures, that are based on public preferences and measured in patients (as required by NICE guidelines (16) and that have been used for economic evaluation modelling studies.(17)

Appendix C

Service Delivery Modelling

Background and rationale

Cancer diagnosis has become one of the principal areas of focus and concern for the NHS in England.(18) For some time, NHS performance in both early diagnosis, delays in referral, and associated survival rates has been poor relative to our national aspirations and when compared with other first world countries. This has worsened during the COVID pandemic. In this context, many of the issues of concern are centred on key aspects of service delivery. How the NHS organises its services is often pivotal in determining the cost, feasibility, and effectiveness. For instance, factors such as workforce availability, prioritisation, service location, scale, and resources are fundamental to the performance of the NHS in delivering effective cancer services.

This component of the ERICA programme will investigate the key factors central to the organisation of NHS diagnostic services for cancer referrals. We will use a range of methods, both quantitative and qualitative, to analyse service delivery alternatives. Specifically, we will aim to build an economic model to assess the likely implications of different scenarios. Implementation of the eRAT diagnostic

For peer review only

tool at primary care level is likely to impact directly on the follow-on pathway for cancer diagnosis (for example in terms of the volume and case mix of referred patients for diagnosis). Our model will therefore provide an assessment of the likely effect of this impact in terms of costs and performance, and highlight any changes in organisation that might be implied by the introduction of the eRAT tool.

This research will run in parallel with the substantive work conducted for the controlled trial of eRAT implementation within ERICA. It will also liaise closely with the detailed and standard analysis of cost-effectiveness for disease progression (which is inherently abstracted from the service delivery aspects of care) in order to provide an added dimension to the cost-effectiveness outputs from the ERICA study as a whole.

Objectives

To build and populate a model of the cancer diagnostic pathway for England, in order to provide an assessment of the costs and effectiveness of different scenarios for service delivery. In particular, we will investigate the potential aspects relative to implementation of eRATs based on the study data collected from the ERICA trial. In addition, qualitative research with NHS staff in secondary care will be used to assess key areas central to successful implementation and sustainability.

Methods

A wide range of methods will be essential to fulfil the objectives of the work outlined here. Early work will include a literature search and survey of current systems for diagnostics in cancer. We will therefore conduct a systematic review of the related literature in the field and carry out a survey of current service delivery organisation across a range of settings. This work will aim to identify the key factors bearing on the organisation of services such a regional variation, metropolitan versus rural context, and population case mix differences.

Phase two work will aim to build a model in order to capture the key elements of service delivery for diagnostic services for cancer. This will explore a range of modelling approaches and test which is most suited to specific needs. For example, discrete event simulation, Systems Dynamics, geographic analysis, and Markov modelling will all be tested in terms of their relevance and appropriateness to specific requirements. In this context it is highly likely that different modelling tools will be relevant to the diverse needs of the study, so no single approach will be dominant.

Phase three will focus on the service delivery implications for the introduction of the eRAT diagnostic tool in primary care looking particularly at the potential knock-on effects in other areas of service.

For peer review only

In addition to our modelling work, we will use qualitative methods, such as problem structuring methods, soft systems mapping, to provide an assessment of some key elements of implementation.

Data

A wide range of data will be used to complete this component of the work. We will aim to integrate sources from across routinely collected datasets such as those listed below to construct our models: NHS activity data, Waiting time data, Reference cost data, Diagnostic Imaging Data (DIDs), Hospital Episode Statistics (HES), Workforce reference data, GP and hospital referral data, QOF data, Population data (e.g. ONS). In addition, we will aim to incorporate the primary data derived from the main ERICA study in order to model and assess the pathway impact from the use of eRATs. We will also use the outputs from the standard economic analysis as an input for the cost effectiveness of the service delivery modelling. Output from the qualitative research will also provide important data for informing the outputs of this work, for example in feeding into the recommendations and conclusions of the study.

Appendix D

Process Evaluation

Scope of process evaluation

The process evaluation work aims to identify and investigate the contextual factors that impact upon the effectiveness of the eRATs with a particular focus on intervention fidelity and GP engagement. The impact of the eRATs on the patients' experience of their GP consultation and their experiences of subsequent care will also be explored. It is underpinned by the COM-B framework for understanding behaviour change (19). This framework will outline the interactive nature of how the GP's capability (IT skill for using the eRATs), opportunity (eRAT prompts), and motivation (to do the training and use all the eRAT features) might influence their behaviour – i.e., ongoing use of the eRATs, symptom checker, coding of symptoms and changes to referral letters). We will use a mixed-methods approach to explore how the intervention was delivered (including fidelity and dose - if the eRATs were being used as intended and their degree of use across intervention practices and over time) and GP engagement with and acceptability of using the eRATs (GP's experiences of the eRATs).(20) For delivery, we will be particularly interested in fidelity of function. (21) GPs will be given clear training videos on how to use the eRATs and we will explore the extent to which GPs engaged with training as well as how they subsequently engaged with the software, and the GP's experiences of how it impacted on the GP-patient relationship in order to evaluate how they responded to the intervention.

For peer review only

Methods

Intervention fidelity and GP engagement (intervention arm only): Prior to the start of the intervention GPs require a minimum level of training in how to use the eRATs. Although the software is designed to be intuitive, a clinical system specific walkthrough for the two main functions of the eRATs (prompt and symptom checker) and FAQs will be available via separate videos. The research champion will be given access to the videos and can disseminate the video content to all GPs in the practice (by showing the videos during a practice meeting, providing a demonstration themselves, or passing on the weblink). Once practices have started the data collection phase, we will invite up to 10 research champions to interview to discuss in depth their experiences of the set-up and training procedures and to explore whether their GPs have the capability, opportunity and motivation to use the eRATs. We will purposively sample research champions based on whether they are from a practice with a high, middle or low two-week wait referral rate, which software system their practice uses, their gender, and their level of experience in practice (10+ years vs. less than 10 years in practice).

Detailed eRAT usage can be captured for all IT systems. Usage will be captured in two ways – i) via a central log and ii) via local 'at practice' reports. For i), usage logs will be routinely and automatically sent from the practice to the Informatica 'digital warehouse' and will contain anonymised, practice-level data for each eRAT including reports of: how many times the prompt was shown, how many times the symptom checker was used, the number of times the symptoms were changed during use of the symptom checker, the length of time the symptom checker was open for, and whether clinical guidance was accessed from the eRAT. These centrally reported logs will be available on a monthly basis throughout the course of the trial and will be securely sent from Informatica to the research team who will add the data to the trial database.

For ii), usage will be examined via reports run locally at each practice. These reports include individual patient level data outlining which eRAT was triggered, the patient's risk score on the eRAT, when the symptom checker was opened and closed, patient's age and sex, and a list of possible eRAT symptoms and whether they were changed. These reports contain depersonalised (pseudo-anonymised) data. As it is possible to potentially identify the patient via the practice ID number we will ask practices to make a copy of the report, add in a new patient study ID variable (e.g., p1, p2, p3, etc) and save it to the practice computer. We will then ask them to send a copy to the trial tram with the original practice ID number removed. They will also send the file with a predetermined practice ID number. These measures should ensure the data is anonymised. The local at practice reports will be securely and electronically transferred to a secure Exeter CTU computer.

For peer review only

Intervention fidelity (Intervention and control). We will ask all research champions in the intervention practice to complete a short questionnaire (online via a secure, University approved provider) detailing their experience of installing software, using the eRATs, and whether alternative risk tools have also been used. We will ask research champions at control practices their experiences of being in the trial and whether they have started using any cancer risk tools. The questionnaires will be completed at two time points – i) within 12 months of the start of the intervention; ii) at the end of the data collection period.

For identifying GPs to interview, we will use maximum variation purposive sampling (sampling on practice two-week wait referral rate (high vs. medium vs. low); software system used, gender, length of time in practice (10+years vs. < 10 years), and working status (part time vs full time)) and expect to interview up to 18 GPs from intervention practices to ask them about their experience of the eRATs including the training provided, any impacts on the consultation and their clinical decision making, as well as any changes in symptom coding behaviour. We will invite GPs to interview after the intervention has been running for at least 3 months. Written information will be provided about the interview study and written consent will be taken prior to the interview and will be verbally confirmed before the interview commences. Interviews will be audio-recorded and carried out by telephone, face-to-face (only if it is safe to do so), or over the internet (e.g., Zoom or MS Teams) depending on the GPs preference, by members of the research team using a pre-defined topic guide that focuses on their training and capability to use eRATs, their opportunity to use the eRATS over the study period and their motivation to continue using the system. If a face-to-face interview is chosen (and safe to perform), interviews will take place in a private room at the practice. The researcher will comply with the lone worker policy, ensuring that have a ‘buddy’ within the research team monitoring their activities and whereabouts. The interviews may raise sensitive issues such as workload and GP overburden or burnout: the interview study information sheet will provide appropriate sources for accessing confidential support. GPs will be reminded that they have the right to not answer any question, stop the interview or withdraw from the interview study; if there is insufficient time to fully discuss issues GP’s will be offered a follow-up time to complete the interview.

GP coding behaviour: It is possible that the eRATs will impact GP coding behaviour - GP coding behaviour for cancer specific symptoms may increase; this would cause a minor increase in triggering of eRATs. We will explore the impact of eRATs on coding behaviour in the interviews (see above) and, resources permitting, will also examine the impact on coding rates using the following approach. We will purposively sample 12 intervention practices and 12 control practices in the South/South West of England based on two-week wait referral rate (i.e., 4 low, 4 moderate, 4 high referring practices) and which software system is being used. In the first instance we will invite practices who are participating

in the nested study to support this work. If insufficient numbers agree, we will approach other practices who are not participating in the nested studies. We will explore the rate of coding of the most frequent symptom for each eRAT cancer in the study that underpins that particular cancer (e.g. cough, abdominal pain, haematuria)(22-25) for a month in the first three months of entry into ERICA, and for the same calendar month a year and two years later (as some symptoms have seasonal variation). This will be performed retrospectively, by the search code being given to the research champion, who will arrange for the search to be conducted in the practice. The results of the search will be emailed to the research team.

Patient experience of care: We will adopt a phased, targeted recruitment strategy with an aim to purposively sample up to (based on two-week rate referral rate (low vs. medium vs. high); gender, age (40-60 vs. 60+)) 32 patients from the intervention arm. We will approach five practices at a time (and expect to recruit around 20 practices to reach the target number of participants), to ensure that we can interview participants in a timely manner.

The in-practice eRAT reports are the mechanism by which we will be able to identify individuals to invite to participate in the activities associated with the process evaluation. The local (at practice) reporting mechanism will allow the research team to identify individuals for whom the eRATs were used and thus who are potentially eligible to participate in a semi-structured interview. Purposive sampling will take place – practices will hold the master eRAT report containing both the patients practice ID number and the new patient study ID. The research team will let the practice know the patient study IDs for those whom an invitation letter will be sent.

Via the GP practice, the research team will send out a letter and information booklet to the identified patients to invite participation in an interview to discuss their experience of care. We will adopt a longitudinal case study design (26) – patients' care pathways will differ, some will receive referrals into secondary care for investigations and tests, while some will be on a 'watch and wait' plan, revisiting their GP at an agreed interval. Some patients will have tests for cancer and the test will indicate that there is no cancer (false positives) whereas some patients will be diagnosed with cancer. So that we can fully capture all patient groups at different stages of their care, individuals will be invited for repeat interviews at regular intervals (i.e., at least one month apart and no more than 3 interviews within 12 months).

We aim to perform the first interview within one month of the consultation in which an eRAT was triggered. Written information about the interview study will have been provided and written informed consent will be taken prior to all interviews, and will be verbally confirmed before the

For peer review only

interview commences. Interviews will be audio-recorded and carried out by members of the research team using pre-defined topic guides. The initial interview will be conducted face-to-face at the participant's home or via video conferencing software such as MS Teams at a time convenient for the participant, with any subsequent interview conducted either face-to-face, over the phone, or via video conference software, depending upon the participant's preference. We will monitor the progression of the Covid-19 pandemic and fully adhere to government advice around social distancing and travel. We will not put the research team or participants at risk and will primarily conduct interviews online. If it is safe to conduct face-to-face interviews, the researcher will comply with the lone worker policy, ensuring that have a 'buddy' within the research team monitoring their activities, whereabouts and expected completion time. The interviews may raise anxiety or concerns related to uncertainty about diagnosis during the referral and investigation period or the watch and wait period; or psychological distress associated with a cancer diagnosis or a false-positive result. The interview study information sheet will provide appropriate sources for accessing confidential support and patients will be reminded that they have the right to not answer any question, stop the interview or withdraw from the interview study.

Management of adverse consequences

As a result of being referred for tests or investigations there is a risk of an adverse incident. If referral rates do increase as a result of access to eRATs, there is an increased risk of an adverse event (AE) to patients of practices allocated to the intervention. We are not routinely tracking individuals throughout the trial and there is no mechanism for monitoring any AEs as a result of referral. However, psychological distress may be a consequence of referral. Individuals for whom cancer is diagnosed at an early stage may be relieved by the diagnosis and see the psychological distress as justifiable. Individuals for whom a referral does not lead to a diagnosis of cancer (false positives) may have undergone unnecessary psychological distress. Our process evaluation work will help us to understand the extent of this and its potential impact on the individuals' life.

During interviews, patients may report being distressed – either as a result of research activity or as a result of their health, and events in their private lives. Should such a situation arise, the researchers will implement the trial risk protocol and manage the participant in accordance with this policy. Participants will be reminded that they have the right to not answer any question, stop the interview or withdraw from the interview study. Under high-risk situations (e.g., where there is perceived immediate risk to a participant's health), the study team may be required to break confidentiality, to inform appropriate authorities who will need to provide essential care services. We will also signpost participants to sources of support. This information will be outlined in the Participant Information Sheet. Participants will be informed of possible benefits and known risks of participation in the

For peer review only

interviews by means of a Patient Information Sheet and through discussion with the research team. Written consent will be obtained immediately prior to the interview study.

There are minimal risks to researchers as most interviews will take place in the GP practices or by telephone/online; however, if a home visit is undertaken to interview patient participants the researcher will follow the lone worker policy: researchers will make sure that their whereabouts, contact telephone number and estimated time of return are known to their colleagues and/or manager. Researchers will also have the opportunity to debrief with a senior colleague on the research team should they need any support after conducting an interview; this debrief may be in person or by telephone.

Analysis

For the quantitative results the individual data sources will be summarised descriptively, including a summary of data completeness. For the qualitative data we will adopt a framework approach (27) which allows the inclusion of key concepts and ideas identified from the literature, alongside themes emerging from the data. The framework approach produces a structured output matrix, with cells of data organised by practice and by code (a descriptive label applied to a section of transcript).

At least two researchers will work on the analysis. Interviews will be audio recorded, transcribed and anonymised. Data familiarisation will be achieved through the listening to and reading of interview recordings and transcripts. Transcripts will be imported into the qualitative data analysis software package NVivo 11 (QSR International) to facilitate data management, sharing and development of a coding framework. A proportion of the interview transcripts will be coded by each researcher. The 'constant comparative method' (28) will be utilised: each incident in the data will be compared with other incidents for similarities and differences and any 'negative cases', where a case does not fit the pattern or cannot be explained by the emerging analysis, will be explored and recorded. Following this initial coding, a PPIE meeting (one for the GP interviews and one for the patient interviews) will be held to discuss the emerging themes from the interviews, and to gain alternative perspectives from the PPIE group on those themes. Following the PPIE meeting, the analytical framework will be developed, incorporating researcher and PPIE perspectives on the results, with a final set of themes and codes being agreed upon.

The analytical framework will be applied to all interview transcripts; one researcher will index all transcripts, with a second researcher indexing a proportion, to check the reliability of the indexing and to ensure that the themes of the framework are being interpreted consistently. Any differences in interpretation will be discussed between the two researchers. Following the indexing process, data

For peer review only

will be charted into the structured output matrix, which will summarise the data on each theme from all transcripts. A subsequent meeting of the PPIE group will be held once all of the results from the process evaluation have been gathered to gain a users' perspective of the global findings.

The final step in the process evaluation analysis will be to integrate results from the various mixed method data sources using a triangulation protocol(29) to give a more complete picture once individual data sources have been individually analysed. We plan to create a summary matrix, known as a convergence coding matrix, which summarises the findings from each data source after assessing whether the findings are in agreement, partial agreement or no agreement, or whether the data source is silent for the finding under consideration i.e. when a theme or finding arises from one data set but not another.

Reporting

The process evaluation results will be briefly summarised for inclusion in the main trial report and publication, separate dissemination (reports, presentations and publications) will provide further details of the process evaluation findings.

Appendix E

GP Workload

Background and rationale

GPs manage a high and rising workload of increasingly complex patient care with many competing demands to attend to within ten-minute consultations. (30) This, combined with ongoing recruitment and retention challenges, has contributed to a GP workforce 'crisis'. (31-36) The workload implications for GPs of using electronic tools such as eRATs during consultations is unclear.(37) ERICA provides an opportunity to examine whether the use of eRATs by GPs, and the possible subsequent discussion of cancer risk with patients, may impact consultation length and patient 'flow' through consulting sessions. This nested study aims to explore, in terms of consultation time, the impact of GPs using eRATs on GP workload and patient 'flow' through consulting sessions. It will also explore workload in the week following the index consultation in which an eRAT was activated, when relevant letters may be generated, referrals made, investigations followed through, or clinical discussions engaged in.

Objectives

The specific objectives in respect of consultations and sessions are:

For peer review only

(i) to measure and compare the duration of consultations and sessions in which an eRAT has been activated with consultations where eRATs have not been activated;

(ii) to measure and compare the duration of subsequent consultations in the same session after an eRAT has been activated with consultations in sessions where eRATs have not been activated;

(iii) to explore the frequency of interactions with patients' medical records by a GP in the week following a consultation during which an eRAT was activated.

Methods

An observational quantitative study will be conducted in a sub-sample of ERICA intervention practices to examine the durations of consultations and consulting sessions in which eRATs are activated.

Sample size

The basis for the sub-study sample size calculation is on the number of consultations likely to occur over a two-week period within ERICA practices, in which an eRAT will be 'activated' (i.e. an eRAT prompt is shown and/or clinician uses an eRAT symptom checker). A number of assumptions are of note:

The first assumption is that a half-day GP consulting session, typically lasting four hours and comprised of ten-minute consultations, would be associated with a total of 24 consultations. Second, practices have an average headcount of seven GPs (informed by GP workforce data from NHS Digital). (38) Third, a GP is assumed to work an average of 6.7 half-day consulting sessions per week. (39) An average practice would therefore provide a total of 1,126 GP consultations per week.

Accurate estimations of how often an eRAT will be activated, are not yet established in previous research on usage of cancer decision tools in UK general practice. (40,41) Two clinical members of the research team have estimated that an eRAT may be expected to be activated once per GP, per week. This estimate would suggest that approximately 15% of consulting sessions will involve a consultation where the eRAT tool was activated.

The standard deviation for both the length of a consultation and of a whole consulting session from previous literature was four minutes and 20 minutes respectively. (42-44) Project team discussion concluded that a minimally important difference in time for an individual consultation would be between two and five minutes; for a consulting session this minimally important difference would be approximately 10 minutes.

For peer review only

Statistical power to detect a time difference of between two and five minutes in eRAT consultations versus non-eRAT consultations is also in excess of >80%, even if eRATs are observed to have been activated in just 1:40 consulting (2.5% of sessions), the basis of the most conservative estimate. The power to detect a difference of 10 minutes in sessions where eRATs have been activated compared with sessions where eRATs have not been activated is >80%, even if eRATs affect only 2.5% of sessions. A two-week observation period would provide sufficient data and power to detect differences in the length of consultations and sessions where an eRAT is activated and those where an eRAT is not activated.

Outcome measures

Primary outcome

The primary outcome is the length of time (in minutes) of consultations. These will be consultations during which an eRAT is activated and also those during which an eRAT is not activated. For the purposes of this sub-study, a consultation is defined as starting when the patient's electronic medical record is opened by a GP, for the purpose of conducting either a face-to-face or telephone/video interaction with the patient, and ending when the record is closed. Home visits will be excluded due to difficulty in accessing accurate time information. Consultations with health professionals who would not make referral decisions (e.g. practice nurses, physiotherapists, pharmacists, healthcare assistants) will also be excluded.

Secondary outcomes

In addition to our primary outcome, we propose to examine the following secondary outcomes:

- The length of time (in minutes) of consulting sessions. For the purposes of this study, a session is defined as a half-day period comprised of individual patients' pre-booked or same-day consultations. The half-day periods are typically 'morning' or 'afternoon', although some practices offer early morning and evening sessions as well. (45)
- The number of instances of opening a patient's electronic medical record in the week following an eRAT being activated.

Practice recruitment

An initial pilot in up to three ERICA intervention practices will be undertaken and plans for data collection methods revisited at that point. Practices will be approached by an invitation email and provided with an information sheet detailing the nature of the study and providing contact details of the researcher. No individual patients will be recruited.

A note on practice recruitment to the nested studies: We expect to recruit up to 91 practices across the nested studies (56 in the health economics nested study, up to 20 in the process evaluation and up to 15 in the sub-study on GP workload) practices across the nested studies. Practices will only be

For peer review only - http://bmjopen.bmj.com/site/about/guidelines.xhtml

asked to help with only one of the health economic nested study, the process evaluation nested study, or the GP workload sub-study – not all three.

Data collection

Identifying consultations where an eRAT is activated

The Process Evaluation describes earlier how a local 'at practice' report will be run for practices in order to collect patient-level data on eRATs usage. This report will be run for practices recruited to this nested study, covering a two-week period.

Measuring durations of consultations and sessions

The eRATs usage report will provide the start and end time of the tool usage, but not the duration of a consultation. A further search function (developed within SystmOne for this nested study) will provide data on the timings of all consultations occurring between two dates (referred to as the 'appointments report'). The consultations identified in the eRAT usage report will be cross-referenced with the consultations in the appointments report. A variable will be added to denote which consultations involved an eRAT being activated and which did not.

Measuring workload in the week following an eRAT being activated

The eRATs usage report will identify the relevant patient records for which an audit will be run in SystmOne. The audit will provide data on instances of the records being opened and closed by practice staff during the week following the index activation of an eRAT.

Data analysis

Data will be analysed in Stata. Descriptive statistics summarising participating practices and GPs will be presented. Although practice level data will be presented, it will be anonymised (e.g. practice A, B, etc) to protect the identities of individual practitioners or practices.

The primary analysis of the durations of consultations in which an eRAT is activated, will take the form of a mixed-effects linear regression with random intercepts to account for clustering within GPs and for GPs clustering within practices. This regression will adjust for consulting GP, time of day, day of week, and consultation mode (face-to-face, telephone, video). Residuals will be checked for normality. As duration data are typically not normally distributed, the data will be transformed if needed, using log transformation. Bootstrapping of the data will also be undertaken if needed. Similar mixed-effects linear regression models with random intercepts will also be performed for secondary outcomes; the duration of consulting sessions, and the number of instances of opening a patient's electronic medical record in the week following an eRAT being activated. For all models where duration is the outcome linear models will be used, but for the count of opening medical records Poisson models will be used.

For peer review only

Governance and ethical considerations

Consent

Individual patient consent is not sought within ERICA for the running of the eRAT usage report. The reports in SystmOne, described for this nested study, will not contain identifiable patient data nor clinically sensitive information and so patient consent for these reports will also not be sought.

Data protection/management and confidentiality

The eRAT usage report and the SystmOne reports will contain pseudo-anonymised data: a patient identifier. However, the reports will contain variables denoting date, time and consulting GP, which will allow cross-referencing, so practices will be asked to delete the patient identifier before sending the report securely and electronically to a secure Exeter CTU computer using a predetermined practice ID number. These measures will ensure the data are anonymised. In the event that the researcher visits the practice to run the SystmOne reports, the files will be anonymised in the same way before the researcher leaves. Practices will keep the original 'master' report files containing the patient's practice computer ID.

Finance

The additional work for the nested study, outside of ERICA costs, is for practices to run the reports in SystmOne and send the report files securely to the researcher. Alternatively, the researcher will visit the practice to run the reports, which may require time of a practice administrator or manager for logging in to the clinical system and orientation. In both scenarios, this time would be covered by nested study research costs at a rate of £50 per hour, and each practice will be offered reimbursement for up to 2 hours. Travel costs for the researcher to visit practices where needed are estimated at £0.45 per mile for a 75 mile round-trip per practice (South West).

References

1. Neal RD, Din NU, Hamilton W, Ukoumunne OC, Carter B, Stapley S, et al. Comparison of cancer diagnostic intervals before and after implementation of NICE guidelines: analysis of data from the UK General Practice Research Database. Br J Cancer. 2014;110(3):584-92.
2. Pickard AS, Neary MP, Cella D. Estimation of minimally important differences in EQ-5D utility and VAS scores in cancer. Health and quality of life outcomes. 2007;5:70-.
3. Kind P, Hardman G, Macran S. UK population norms for EQ-5D. Centre for Health Economics, University of York; 1999.
4. Man M-S, Chaplin K, Mann C, Bower P, Brookes S, Fitzpatrick B, et al. Improving the management of multimorbidity in general practice: protocol of a cluster randomised controlled trial (The 3D Study). BMJ Open. 2016;6(4):e011261.
5. Curtis L. Unit Costs of Health and Social Care 2020 [Available from: https://www.pssru.ac.uk/project-pages/unit-costs/unit-costs-2020/]. 2020.
6. Department of Health and Social Care. NHS reference costs 2019 to 2020 [Available from: https://improvement.nhs.uk/resources/reference-costs/]. 2020.

7. Caro JJ, Briggs AH, Siebert U, Kuntz KM. Modeling good research practices--overview: a report of the ISPOR-SMDM Modeling Good Research Practices Task Force-1. Medical decision making : an international journal of the Society for Medical Decision Making. 2012;32(5):667-77.
8. Siebert U, Alagoz O, Bayoumi AM, Jahn B, Owens DK, Cohen DJ, et al. State-Transition Modeling:A Report of the ISPOR-SMDM Modeling Good Research Practices Task Force–3. Medical Decision Making. 2012;32(5):690-700.
9. Schwander B, Hiligsmann M, Nuijten M, Evers S. Systematic review and overview of health economic evaluation models in obesity prevention and therapy. Expert review of pharmacoeconomics & outcomes research. 2016;16(5):561-70.
10. Wang BC, Furnback W. Modelling the long-term outcomes of bariatric surgery: A review of cost-effectiveness studies. Best practice & research Clinical gastroenterology. 2013;27(6):987-95.
11. Andronis L, Barton P, Bryan S. Sensitivity analysis in economic evaluation: an audit of NICE current practice and a review of its use and value in decision-making. Health Technol Assess. 2009;13(29):iii, ix-xi, 1-61.
12. Fenwick E, O'Brien BJ, Briggs A. Cost-effectiveness acceptability curves--facts, fallacies and frequently asked questions. Health economics. 2004;13(5):405-15.
13. Husereau D, Drummond M, Petrou S, Carswell C, Moher D, Greenberg D, et al. Consolidated Health Economic Evaluation Reporting Standards (CHEERS) statement. Value Health. 2013;16(2):e1-5.
14. NICE. Guide to the Methods of Technology Appraisals, Section 5.6 Discounting [Available from: https://www.nice.org.uk/process/pmg9/chapter/the-reference-case#discounting]. 2013.
15. Faria R, Gomes M, Epstein D, White IR. A guide to handling missing data in cost-effectiveness analysis conducted within randomised controlled trials. PharmacoEconomics. 2014;32(12):1157-70.
16. Brazier J, Longworth L. NICE DSU Technical Support Document 8: An introduction to the measurement and valuation of health for NICE submissions. In: Health, editor. 2011.
17. Tengs TO, Wallace A. One thousand health-related quality-of-life estimates. Medical care. 2000;38(6):583-637.
18. NHS England. (2019) The NHS Long Term Plan [Available from: https://www.longtermplan.nhs.uk/.
19. Michie S, van Stralen MM, West R. The behaviour change wheel: a new method for characterising and designing behaviour change interventions. Implementation science : IS. 2011;6:42-.
20. Grant A, Treweek S, Dreischulte T, Foy R, Guthrie B. Process evaluations for cluster-randomised trials of complex interventions: a proposed framework for design and reporting. Trials. 2013;14:15.
21. Hawe P, Shiell A, Riley T. Complex interventions: how "out of control" can a randomised controlled trial be? BMJ (Clinical research ed). 2004;328(7455):1561-3.
22. Shephard EA, Stapley S, Neal RD, Rose P, Walter FM, Hamilton WT. Clinical features of bladder cancer in primary care. The British journal of general practice : the journal of the Royal College of General Practitioners. 2012;62(602):e598-604.
23. Shephard E, Neal R, Rose P, Walter F, Hamilton WT. Clinical features of kidney cancer in primary care: a case-control study using primary care records. The British journal of general practice : the journal of the Royal College of General Practitioners. 2013;63(609):e250-5.
24. Hamilton W. The CAPER studies: five case-control studies aimed at identifying and quantifying the risk of cancer in symptomatic primary care patients. Br J Cancer. 2009;101 Suppl 2:S80-6.
25. Stapley S, Peters TJ, Neal RD, Rose PW, Walter FM, Hamilton W. The risk of oesophago-gastric cancer in symptomatic patients in primary care: a large case-control study using electronic records. Br J Cancer. 2013;108(1):25-31.
26. Yin R. Case Study Research: Design and Methods. London: Sage Publications 2003.
27. Ritchie J, Spencer L, O'Connor W. Carrying out Qualitative Analysis. In: Ritchie J, Lewis J, editors. Qualitative Research Practice, A guide for Social Science Students and Researchers London: Sage; 2003.
28. Corbin J, Strauss A. Basics of qualitative research: Techniques and procedures for developing grounded theory. London: Sage publications; 2014.
29. O'Cathain A, Murphy E, Nicholl J. Three techniques for integrating data in mixed methods studies. BMJ. 2010;341:c4587.
30. Hobbs FDR, Bankhead C, Mukhtar T, Stevens S, Perera-Salazar R, Holt T, et al. Clinical workload in UK primary care: a retrospective analysis of 100 million consultations in England, 2007-14. Lancet 2016;387:2323-30. http://dx.doi.org/10.1016/S0140-6736(16)00620-6
31. Baird B, Charles A, Honeyman M, Maguire D, Das P. Understanding pressures in general practice: The Kings Fund; 2016.
32. Campbell J CR, Sansom A. Retaining the experienced GP workforce in direct patient care (ReGROUP): final report. Exeter: University of Exeter Medical School; 2015.

33. Sansom A, Terry R, Fletcher E, Salisbury C, Long L, Richards SH, et al. Why do GPs leave direct patient care and what might help to retain them? A qualitative study of GPs in South West England. BMJ Open 2018;8:e019849. http://dx.doi.org/10.1136/bmjopen-2017-019849
34. Roland M, Everington S. Tackling the crisis in general practice. BMJ 2016;352:i942. http://dx.doi.org/10.1136/bmj.i942
35. Fletcher E, Abel GA, Anderson R, Richards SH, Salisbury C, Dean SG, et al. Quitting patient care and career break intentions among general practitioners in South West England: findings of a census survey of general practitioners. BMJ Open 2017;7:e015853. http://dx.doi.org/10.1136/bmjopen-2017-015853
36. Walker B, Moss, C., Gibson, J., Sutton, M., Spooner, S., Checkland, K. Tenth National GP Worklife Survey. Manchester: Policy Research Unit in Commissioning and the Healthcare System Manchester Centre for Health Economics; 2019.
37. Fletcher E, Burns, A., Wiering, B., Lavi, D., Shephard, E., Hamilton, W., Campbell, J. L., Abel, G. Workload and workflow implications associated with the use of electronic risk assessment tools used by health professionals in general practice: a scoping review. In: University of Exeter Medical School; 2022.
38. NHS Digital. General Practice Workforce 30 September 2019. NHS Digital; 2019. URL: https://digital.nhs.uk/data-and-information/publications/statistical/general-and-personal-medical-services/final-30-september-2019#resources (Accessed 11/02/20).
39. Gibson J, Sutton M, Spooner S, Checkland K. Ninth National GP Worklife Survey 2017. In; 2017.
40. Moffat J, Ironmonger L. Clinical Decision Support Tool for Cancer (CDS) Project evaluation report to the Department of Health; 2014.
41. Robinson R, Poirier V, Watson S. Using Cancer Decision Support Tools to support the early diagnosis of cancer; 2017.
42. Salisbury C, Procter S, Stewart K, Bowen L, Purdy S, Ridd M, et al. The content of general practice consultations: cross-sectional study based on video recordings. British Journal of General Practice 2013;63:e751. http://dx.doi.org/10.3399/bjgp13X674431
43. Elmore N, Burt J, Abel G, Maratos FA, Montague J, Campbell J, et al. Investigating the relationship between consultation length and patient experience: a cross-sectional study in primary care. Br J Gen Pract 2016;66:e896-e903. http://dx.doi.org/10.3399/bjgp16X687733
44. Orton PK, Pereira Gray D. Factors influencing consultation length in general/family practice. Fam Pract 2016;33:529-34. http://dx.doi.org/10.1093/fampra/cmw056
45. NHS. Working life (GP). NHS; 2022. URL: https://www.healthcareers.nhs.uk/explore-roles/doctors/roles-doctors/general-practice-gp/working-life (Accessed 2022).

For peer review only

STANDARD PROTOCOL ITEMS: RECOMMENDATIONS FOR INTERVENTIONAL TRIALS

SPIRIT 2013 Checklist: Recommended items to address in a clinical trial protocol and related documents*

Section/item	Item No	Description	Addressed on page number
**Administrative information**			
Title	1	Descriptive title identifying the study design, population, interventions, and, if applicable, trial acronym	Title page
Trial registration	2a	Trial identifier and registry name. If not yet registered, name of intended registry	1 & 15
	2b	All items from the World Health Organization Trial Registration Data Set	n/a
Protocol version	3	Date and version identifier	1 & 15
Funding	4	Sources and types of financial, material, and other support	15
Roles and responsibilities	5a	Names, affiliations, and roles of protocol contributors	15
	5b	Name and contact information for the trial sponsor	1
	5c	Role of study sponsor and funders, if any, in study design; collection, management, analysis, and interpretation of data; writing of the report; and the decision to submit the report for publication, including whether they will have ultimate authority over any of these activities	12
	5d	Composition, roles, and responsibilities of the coordinating centre, steering committee, endpoint adjudication committee, data management team, and other individuals or groups overseeing the trial, if applicable (see Item 21a for data monitoring committee)	12-13

For peer review only

Introduction

Background and rationale	6a	Description of research question and justification for undertaking the trial, including summary of relevant studies (published and unpublished) examining benefits and harms for each intervention	2-3
	6b	Explanation for choice of comparators	5-7
Objectives	7	Specific objectives or hypotheses	3-4
Trial design	8	Description of trial design including type of trial (eg, parallel group, crossover, factorial, single group), allocation ratio, and framework (eg, superiority, equivalence, noninferiority, exploratory)	4

Methods: Participants, interventions, and outcomes

Study setting	9	Description of study settings (eg, community clinic, academic hospital) and list of countries where data will be collected. Reference to where list of study sites can be obtained	4
Eligibility criteria	10	Inclusion and exclusion criteria for participants. If applicable, eligibility criteria for study centres and individuals who will perform the interventions (eg, surgeons, psychotherapists)	7
Interventions	11a	Interventions for each group with sufficient detail to allow replication, including how and when they will be administered	4-5
	11b	Criteria for discontinuing or modifying allocated interventions for a given trial participant (eg, drug dose change in response to harms, participant request, or improving/worsening disease)	n/a
	11c	Strategies to improve adherence to intervention protocols, and any procedures for monitoring adherence (eg, drug tablet return, laboratory tests)	14 and Appx D
	11d	Relevant concomitant care and interventions that are permitted or prohibited during the trial	4-7
Outcomes	12	Primary, secondary, and other outcomes, including the specific measurement variable (eg, systolic blood pressure), analysis metric (eg, change from baseline, final value, time to event), method of aggregation (eg, median, proportion), and time point for each outcome. Explanation of the clinical relevance of chosen efficacy and harm outcomes is strongly recommended	8-9
Participant timeline	13	Time schedule of enrolment, interventions (including any run-ins and washouts), assessments, and visits for participants. A schematic diagram is highly recommended (see Figure)	6

For peer review only - http://bmjopen.bmj.com/site/about/guidelines.xhtml

Sample size	14	Estimated number of participants needed to achieve study objectives and how it was determined, including clinical and statistical assumptions supporting any sample size calculations	6-7
Recruitment	15	Strategies for achieving adequate participant enrolment to reach target sample size	7

Methods: Assignment of interventions (for controlled trials)

Allocation:

Sequence generation	16a	Method of generating the allocation sequence (eg, computer-generated random numbers), and list of any factors for stratification. To reduce predictability of a random sequence, details of any planned restriction (eg, blocking) should be provided in a separate document that is unavailable to those who enrol participants or assign interventions	7
Allocation concealment mechanism	16b	Mechanism of implementing the allocation sequence (eg, central telephone; sequentially numbered, opaque, sealed envelopes), describing any steps to conceal the sequence until interventions are assigned	7
Implementation	16c	Who will generate the allocation sequence, who will enrol participants, and who will assign participants to interventions	7
Blinding (masking)	17a	Who will be blinded after assignment to interventions (eg, trial participants, care providers, outcome assessors, data analysts), and how	7-8
	17b	If blinded, circumstances under which unblinding is permissible, and procedure for revealing a participant's allocated intervention during the trial	n/a

Methods: Data collection, management, and analysis

Data collection methods	18a	Plans for assessment and collection of outcome, baseline, and other trial data, including any related processes to promote data quality (eg, duplicate measurements, training of assessors) and a description of study instruments (eg, questionnaires, laboratory tests) along with their reliability and validity, if known. Reference to where data collection forms can be found, if not in the protocol	9-12
	18b	Plans to promote participant retention and complete follow-up, including list of any outcome data to be collected for participants who discontinue or deviate from intervention protocols	11-12

For peer review only - http://bmjopen.bmj.com/site/about/guidelines.xhtml

Data management	19	Plans for data entry, coding, security, and storage, including any related processes to promote data quality (eg, double data entry; range checks for data values). Reference to where details of data management procedures can be found, if not in the protocol	11-12
Statistical methods	20a	Statistical methods for analysing primary and secondary outcomes. Reference to where other details of the statistical analysis plan can be found, if not in the protocol	9-11
	20b	Methods for any additional analyses (eg, subgroup and adjusted analyses)	11
	20c	Definition of analysis population relating to protocol non-adherence (eg, as randomised analysis), and any statistical methods to handle missing data (eg, multiple imputation)	11
**Methods: Monitoring**			
Data monitoring	21a	Composition of data monitoring committee (DMC); summary of its role and reporting structure; statement of whether it is independent from the sponsor and competing interests; and reference to where further details about its charter can be found, if not in the protocol. Alternatively, an explanation of why a DMC is not needed	12-13
	21b	Description of any interim analyses and stopping guidelines, including who will have access to these interim results and make the final decision to terminate the trial	n/a
Harms	22	Plans for collecting, assessing, reporting, and managing solicited and spontaneously reported adverse events and other unintended effects of trial interventions or trial conduct	9, Appendix D
Auditing	23	Frequency and procedures for auditing trial conduct, if any, and whether the process will be independent from investigators and the sponsor	n/a
**Ethics and dissemination**			
Research ethics approval	24	Plans for seeking research ethics committee/institutional review board (REC/IRB) approval	15
Protocol amendments	25	Plans for communicating important protocol modifications (eg, changes to eligibility criteria, outcomes, analyses) to relevant parties (eg, investigators, REC/IRBs, trial participants, trial registries, journals, regulators)	12-13

For peer review only - http://bmjopen.bmj.com/site/about/guidelines.xhtml

Consent or assent	26a	Who will obtain informed consent or assent from potential trial participants or authorised surrogates, and how (see Item 32)	11 & Appendices B & D (only relevant for nested studies)
	26b	Additional consent provisions for collection and use of participant data and biological specimens in ancillary studies, if applicable	11 & Appendices B & D (only relevant for nested studies)
Confidentiality	27	How personal information about potential and enrolled participants will be collected, shared, and maintained in order to protect confidentiality before, during, and after the trial	11-12
Declaration of interests	28	Financial and other competing interests for principal investigators for the overall trial and each study site	15
Access to data	29	Statement of who will have access to the final trial dataset, and disclosure of contractual agreements that limit such access for investigators	14
Ancillary and post-trial care	30	Provisions, if any, for ancillary and post-trial care, and for compensation to those who suffer harm from trial participation	n/a
Dissemination policy	31a	Plans for investigators and sponsor to communicate trial results to participants, healthcare professionals, the public, and other relevant groups (eg, via publication, reporting in results databases, or other data sharing arrangements), including any publication restrictions	14
	31b	Authorship eligibility guidelines and any intended use of professional writers	14
	31c	Plans, if any, for granting public access to the full protocol, participant-level dataset, and statistical code	14
**Appendices**			
Informed consent materials	32	Model consent form and other related documentation given to participants and authorised surrogates	Not applicable for main trial. Multiple documents for each nested study, available from authors upon request

For peer review only - http://bmjopen.bmj.com/site/about/guidelines.xhtml

Biological specimens	33	Plans for collection, laboratory evaluation, and storage of biological specimens for genetic or molecular analysis in the current trial and for future use in ancillary studies, if applicable	n/a

*It is strongly recommended that this checklist be read in conjunction with the SPIRIT 2013 Explanation & Elaboration for important clarification on the items. Amendments to the protocol should be tracked and dated. The SPIRIT checklist is copyrighted by the SPIRIT Group under the Creative Commons "Attribution-NonCommercial-NoDerivs 3.0 Unported" license.

For peer review only

For peer review only - http://bmjopen.bmj.com/site/about/guidelines.xhtml

BMJ Open

Protocol for a pragmatic cluster randomised controlled trial assessing the clinical effectiveness and cost-effectiveness of electronic risk-assessment for cancer for patients in general practice (ERICA)

Journal:	*BMJ Open*
Manuscript ID	bmjopen-2022-065232.R1
Article Type:	Protocol
Date Submitted by the Author:	20-Jan-2023
Complete List of Authors:	Hamilton, Willie; University of Exeter, Primary Care Diagnostics<br>Mounce, Luke; University of Exeter, Institute of Health Research<br>Abel, Gary; University of Exeter, University of Exeter Medical School (Primary Care)<br>Dean, Sarah; PenCLAHRC University of Exeter Medical School,<br>Campbell, John; University of Exeter, Primary Care<br>Warren, Fiona; University of Exeter Medical School, Institute of Health Research<br>Spencer, Anne; University of Exeter Medical School, Health Economics<br>Medina-Lara, Antonieta; University of Exeter Medical School, Health Economics Group<br>Pitt, Martin; University of Exeter, University of Exeter: Medical School<br>Shephard, Elizabeth; University of Exeter,<br>Shakespeare, Marijke; University of Exeter Medical School, Primary Care<br>Fletcher, Emily; University of Exeter Medical School, Primary Care Research Group<br>Mercer, Adrian; University of Exeter Medical School, Primary Care<br>Calitri, Raff; University of Exeter Medical School, Primary Care
**Primary Subject Heading**:	General practice / Family practice
Secondary Subject Heading:	Health services research
Keywords:	HEALTH SERVICES ADMINISTRATION & MANAGEMENT, Risk management < HEALTH SERVICES ADMINISTRATION & MANAGEMENT, PRIMARY CARE, Clinical trials < THERAPEUTICS

SCHOLARONE™
Manuscripts

Protocol for a pragmatic cluster randomised controlled trial assessing the clinical effectiveness and cost-effectiveness of electronic risk-assessment for cancer for patients in general practice (ERICA)

Willie Hamilton,[1] Luke Mounce, [1] Gary Abel, [1] Sarah Dean,[1] John Campbell, [1] Fiona Warren,[1] Anne Spencer, [1] Antonieta Medina-Lara, Martin Pitt, [1] Elizabeth Shephard,[1] Marijke Shakespeare, [1] Emily Fletcher, [1] Adrian Mercer,[1] Raff Calitri.[1]

[1]University of Exeter, St Luke's campus, Exeter, EX1 2LU

Willie Hamilton, Professor of Primary Care Diagnostics: w.hamilton@exeter.ac.uk, ORCID ID 0000-0003-1611-1373 (Corresponding author)

Luke Mounce, Research Fellow: l.t.a.mounce@exeter.ac.uk, ORCID ID 0000-0002-6089-0661

Gary Abel, Associate Professor: g.a.abel@exeter.ac.uk, ORCID ID 0000-0003-2231-5161

Sarah G Dean, Professor in Psychology Applied to Rehabilitation and Health: s.dean@exeter.ac.uk, ORCID ID 0000-0002-3682-5149

John Campbell, Professor of General Practice and Primary Care: john.compbell@exeter.ac.uk, ORCID ID 0000-0002-6752-3493

Fiona Warren, Senior Lecturer in Medical Statistics: f.c.warren@exeter.ac.uk, ORCID ID 0000-0002-3833-0182

Anne Spencer, Associate Professor: a.e.spencer@exeter..ac.uk ORCID ID 0000-0002-8163-3103

Antonieta Medina-Lara, Associate Professor in Health Economics: a.medina-lara@exeter.ac.uk, ORCID ID 0000-0001-7325-8246

Martin Pitt, Professor of Applied Healthcare Modelling and Data Science: m.pitt@exeter.ac.uk

Elizabeth Shephard, Research Fellow: e.a.shephard@exeter.ac.uk, ORCID ID 0000-0002-3610-3680

Marijke Shakespeare, Trial coordinator: m.shakespeare@exeter.ac.uk

Emily Fletcher, Research Fellow: e.fletcher@exeter.ac.uk, ORCID ID 0000-0003-1319-3051

Adrian Mercer, PPIE Representative, zante256@gmail.com

Raff Calitri, Research Fellow and Trial Manager, r.calitri@exeter.ac.uk, ORCID ID 0000-0003-0889-4670

For peer review only

Abstract

Introduction. The UK has worse cancer outcomes than most comparable countries, with a large contribution attributed to diagnostic delay. Electronic risk assessment tools (eRATs) have been developed to identify primary care patients with a ≥2% risk of cancer using features recorded in the electronic record.

Methods and analysis. This is a pragmatic cluster randomised controlled trial in English primary care. Individual general practices will be randomised in a 1:1 ratio to intervention (provision of eRATs for six common cancer sites) or to usual care. The primary outcome is cancer stage at diagnosis, dichotomised to stage 1 or 2 (early) or stage 3 or 4 (advanced) for these six cancers, assessed from national cancer registry data. Secondary outcomes include stage at diagnosis for a further six cancers without eRATs, use of urgent referral cancer pathways, total practice cancer diagnoses, routes to cancer diagnosis, and 30-day and 1-year cancer survival. Economic and process evaluations will be performed along with service delivery modelling. The primary analysis explores the proportion of cancer patients with early stage at diagnosis. The sample size calculation used an odds ratio of 0.8 for a cancer being diagnosed at advanced stage in the intervention arm compared with the control arm, equating to an absolute reduction of 4.8% as an incidence-weighted figure across the six cancers. This requires 530 practices overall, with the intervention active from April 2022 for 2 years.

Ethics and dissemination. The trial has approval from London City & East Research Ethics committee, reference number 19/LO/0615; protocol version 5.0, 9th May 2022. It is sponsored by the University of Exeter. Dissemination will be by journal publication, conferences, use of appropriate social media and direct sharing with cancer policymakers.

Registration. The trial is registered with ISRCTN: (trial no: ISRCTN22560297).

Word Count: 5665

Key words: Early cancer diagnosis, randomised controlled trial, clinical risk-assessment tools, General Practice

For peer review only - http://bmjopen.bmj.com/site/about/guidelines.xhtml

Article summary

Strengths and limitations of this study

- Improvements in primary care are seen as a key for improving early cancer diagnosis in the UK, and this trial is targeting that part of the diagnostic pathway.
- This is a large, definitive trial, powered to identify a clinically important difference in cancer stage at diagnosis.
- The trial is designed to minimise impact on participating practices with outcome data being obtained from routinely collected National Health Service data.
- One limitation is that the UK's national imperative to improve cancer diagnosis after the COVID pandemic may mean use of other interventions (or eRATs themselves) are encouraged by policymakers, reducing the validity and reliability of the trial.

For peer review only

Introduction

An estimated 10,000 UK cancer deaths each year would not occur if the UK matched the outcomes of other European countries.(1) Much of the difference is attributed to diagnostic delay.(2) The NHS Long Term plan, published in January 2019, specifically targets an increase in the percentage of cancer patients whose cancer is stage 1 or 2 (thus potentially curable) at diagnosis to rise from the current 54% to 75% by 2028.(3) Diagnosis of cancer may occur by several routes, but the main ones are population screening, and diagnosis after symptoms have occurred. Although screening for cancer is effective for colorectal, breast, lung and cervical cancers,(4-6) less than 10% of the total new UK cancers are identified by this route. Most of the remainder are diagnosed after presenting with symptoms, usually to primary care. Of patients with cancer, just under 20% present with an emergency complication of their cancer; however, many of these patients have previously reported symptoms attributable to their cancer to primary care, but this presentation did not lead to a diagnosis of cancer.(7) Within general practice, many studies have aimed at identifying the symptoms of possible cancer and quantifying their predictive value.(8) One main output has been Risk Assessment Tools (generally abbreviated to RATs); these give precise estimates of the chance of an underlying cancer as a percentage figure. RATs provide precise estimates for single symptoms (e.g. the risk of cancer of the lung for a person aged 40 years or more with haemoptysis is 2.4%), as pairs of symptoms (haemoptysis accompanied by loss of weight is 9.2%) or as repeated symptoms (a re-attendance with haemoptysis is 17%).(9) RATs are published for the 18 most common adult cancers, accounting for nearly 90% of the total cancer burden. These publications have been highly influential: in particular, they strongly contributed to the National Institute of Healthcare Excellence (NICE) guideline, Suspected cancer: recognition and referral [NG12], which guides symptomatic diagnosis of cancer in the UK.(10)

For peer review only - http://bmjopen.bmj.com/site/about/guidelines.xhtml

The initial RATs, of paper, mouse mat, calendar, or web-based forms, increased cancer diagnostic activity,(11) though impacts on hard outcomes such as stage at diagnosis or cancer survival were unknown. Electronic RATs (eRATs) for seven major cancers (lung, colorectal, pancreas, oesophago-gastric, bladder, kidney and ovary) have been developed for the two largest UK primary care electronic healthcare record systems, SystmOne and EMIS, used in around 80% of English practices. The software performs daily calculations of individual cancer risk in patients aged 40 and over, using coded symptoms and laboratory results in the patient's record over the past year, and prompts the general practitioner (GP) when the risk of one or more of these cancers is equal to or above 2%. Some form of electronic clinical decision support for cancer diagnosis has been downloaded by practices and used by at least one practice member in approximately 12% of English practices.(12). Two systematic reviews recently concluded that more research evidence was needed for impact on time to diagnosis and treatment, stage at diagnosis, and health outcomes, as well as research to understand how tools are used in GP consultations.(13) A feasibility trial of the oesophago-gastric eRAT published after these systematic reviews reported installation and regulatory problems that severely restricted usage,(14) and a vignette study of the colorectal RAT suggested it changed the GP's inclination to refer in 26% of usages.(15)

One crucial aspect of eRAT research relates to cost-effectiveness: annual NHS spending on cancer diagnosis is approximately £1bn.(16) Observational data showed increased use of the urgent cancer referral system to improve survival,(17) but there is insufficient data to inform a cost-effectiveness analysis of the subject.(13)

Objectives

The overarching aim of the trial is to assess the clinical and cost-effectiveness of using eRATs for six cancer sites – colorectal, lung, bladder, kidney, oesophago-gastric and ovarian cancers - compared with usual care for patients in general practice. Our hypothesis is that provision of eRATs will expedite the diagnosis of symptomatic cancer resulting in better cancer outcomes.

The primary objective is to compare the effects of using eRATs (vs usual care) on the percentage of patients with a newly diagnosed cancer at one of the six sites whose cancer is staged as being stage 1 or 2 (versus stage 3 or 4).

A secondary objective is to investigate differences in the stage at diagnosis of a further six cancers without eRATs (combined): breast, melanoma, prostate, Non-Hodgkin lymphoma, larynx and uterus. This is to investigate the possibility of an effect whereby eRATs are associated with increased

For peer review only - http://bmjopen.bmj.com/site/about/guidelines.xhtml

diagnostic activity beyond the eRAT cancers. We will also investigate differences in: the number of patients diagnosed with the six eRAT cancers combined, and the total number of cancers (excluding non-melanoma skin cancer) diagnosed, use of the 2-week wait referral system (the main pathway for urgent investigation of possible cancer in England) or equivalent for the six eRAT cancers combined, and across all cancers; the routes to diagnosis for each of the six eRAT cancers,(18) and for the six comparator non-eRAT cancers; the proportion of patients on a 2-week wait pathway receiving a diagnosis of cancer; whether a patient on a 2-week wait pathway has a diagnosis of cancer established (or refuted) within 28 days; 30-day and 1-year survival for those with cancer; the rate of cancer investigations, namely colonoscopies, sigmoidoscopies, upper gastro-intestinal endoscopies, chest x-rays, abdominal ultrasounds, and abdominal CT scans. We will also conduct parallel cost-effectiveness analyses, service delivery modelling and a process evaluation.

Methods and analysis

Design and setting

The study is a pragmatic cluster randomised-controlled trial in England, in primary care medical practices using one of the two (SystmOne or EMIS) electronic record keeping systems. The clusters are practices, a term which includes single practices, and small groups of practices agglomerated administratively to single entities. These will be randomised 1:1 to receive either the intervention (access to the suite of eRATs) or usual care. It is unrealistic to offer eRATs to individual GPs, as there would be considerable contamination within any practice. Nevertheless, for a practice to be eligible to take part, we ask at least 50% of GPs in that practice to agree to use the eRATs. Although the intervention is at the practice level, some process and resource use measures and all main trial primary and secondary outcomes relate to individual patients.

Intervention

The eRATs

The eRATs have been developed by a specialist IT team, Informatica systems Ltd, in partnership with the cancer charity, Macmillan. The risk estimates in the eRATs are from the original research papers for each cancer site. (9, 19-24) Practices will access the software via a new cloud-based system called Skyline, specifically designed to facilitate efficient integration into GP clinical systems. CA marking of the Skyline version of eRATs was obtained in September 2021.

The eRATs have multiple functions. The first is the *'prompt'*. This collates relevant coded symptoms and blood tests in the patient's medical record from the previous 12 months, which are then assessed for the possibility of cancer, generating a risk score equivalent to the positive predictive value of the cancer features for each cancer. A prompt (pop-up), displaying the risk score(s), appears on screen

For peer review only - http://bmjopen.bmj.com/site/about/guidelines.xhtml

when a registered user opens a patient's medical records and indicates that patient has a risk of 2% or higher for at least one of the studied cancers. A second function is the *'symptom checker'*, allowing the clinician to add additional patient's symptoms to the eRAT checklist on screen; this process automatically recalculates the risk of any of the six cancers. On reviewing the risk score from the prompt and/or symptom checker, the clinician then decides the best course of management, which may be: (i) clinical review in primary care; (ii) ordering of test/investigations; or (iii) referral into secondary care. Embedded within all eRATS are links to authoritative guidance regarding the early diagnosis of cancer, NICE NG12,(25), Macmillan's abbreviated NICE guidance,(26) and Cancer Research UK guidance. (27) These sources of information are added to assist management of the patient, but the decision whether – or not – to investigate is for the clinician and patient. Some EMIS practices also have access to the QCancer risk tool, (28) albeit embedded in a dormant state within the practice IT and record system, and requiring manual activation prior to operation. All practices will be asked not to use it during the trial.

Justification of cancer sites

RATs are available for 18 adult cancers, each varying in their incidence, ease of diagnosis, amenability to treatment and proportion presenting as an emergency.

We elected to study cancer sites a) which were in the top 15 cancers by incidence; b) for which curative treatment is reasonably possible in symptomatic patients;(29) and c) with a significant percentage of patients presenting as an emergency.(30). Using these criteria, six cancer sites were selected, amounting to approximately half of all incident cancers. The selected six were: lung, colorectal, oesophago-gastric, ovary, kidney and bladder. The remaining nine cancers were considered as comparators to examine for any practice level effect of increased cancer diagnostic activity. Three of these nine cancers, brain, pancreas and leukaemia, were removed for clinical and practical reasons: no eRAT is available for brain or leukaemia; in both brain and pancreas, symptomatic diagnosis is considered to have a very small likelihood of improving survival,(29) and in leukaemia, a full blood count (easily available in primary care) will usually establish the diagnosis, making an eRAT unlikely to expedite the diagnosis.(31)

Training practices in using eRATs

Training in the use of the eRATs uses short, pre-recorded videos available online co-ordinated by a practice 'research champion'. These show GPs how to use the prompt and symptom checker functions.

For peer review only - http://bmjopen.bmj.com/site/about/guidelines.xhtml

Duration of intervention

Practice recruitment started in August 2019 and is expected to finish at the end of March 2022, including the installation of the eRATs software. The trial was paused for 6 months in March 2020 due to Covid-19. The formal start of the intervention window will be 01/04/2022 (although some practices may have delayed installation) and will close for all intervention practices on 31/3/2024.

Usual care

Patients presenting to the control practices will experience the GP's usual diagnostic approach. GPs in control practices will have no specific on-screen prompt, though they may have access to hard-copy (e.g. paper or mouse mat) versions of the RATs, or to other cancer tools such as those supporting structured follow-up of symptomatic patients not selected for initial investigation. For EMIS practices with QCancer dormant in the system, control practices are expected to leave it dormant. We will document control practice use of RATs, other decision support tools, and access to and use of eRATs via interim and exit questionnaires completed within the first 12 months of a practice commencing the intervention and at the end of the trial. In line with intervention practices, trial time will formally begin for control practices on 01/04/2022 and end on 31/03/2024.

Data collection window

Outcome data for all practices will be obtained for the 2-year period from 01/06/2022 to 29/05/2024. This data collection window is lagged behind the trial time window (01/04/2022 to 31/03/2024) in order to: a) provide some time for practices to become accustomed to how the intervention functions prior to data collection, and b) to have a 2-month window following the end of the intervention window in order to allow cancers to be diagnosed in patients seen towards the end of that window.

Sample size

There are around 130,000 new diagnoses of the six included cancers in the UK annually.(32) As each of our six cancer sites has different proportions diagnosed at an early stage, the sample size calculation is based on a relative improvement in staging, using an odds ratio of 0.8 for a cancer being diagnosed at Stage 3/4 in the intervention arm compared with the control arm. This difference is quite large and equates to an absolute reduction of 4.8% in the intervention arm as an incidence-weighted figure across the six cancers. A much smaller improvement would still be clinically valuable but would necessitate an impossibly large trial.

For the inflation factor we have used an intra-cluster correlation coefficient based on our previous work, of 0.05.(33) An average cluster size of 23 patients with a diagnosed cancer with recorded stage during 2-year follow-up is expected, with a coefficient of variation for cluster size of 0.7, giving a design

For peer review only - http://bmjopen.bmj.com/site/about/guidelines.xhtml

For peer review only

effect of 2.66. For an individually randomised trial with 90% power and an alpha threshold of 0.05, the sample size would be 2,049 patients per arm. Adding in the design effect, this becomes 5,497 patients, requiring 239 practices per arm, and 478 practices in total. Due to changes in practice structure (such as practice mergers, closures or divisions), we anticipate the loss of up to 10% of recruited practices over the course of the trial; to account for this we will recruit a target of 530 practices overall, expecting 12,190 patients to be diagnosed with cancer in total.

Practice recruitment

A total of 530 primary care practices across England will be recruited, supported by the NIHR Clinical Research Network (CRN) and strategic media releases to raise awareness of the trial. Practices that are proposing a split or a merger are not eligible for the trial, as the practices before or after the change may have been allocated to different arms in the trial. A method for identifying and managing unanticipated splits or mergers during the active phase of the trial is shown in Appendix A.

Patients are not being recruited into this trial - patient consent is not being sought for the use of the eRATs during the consultation. This is because ERATs are essentially an extension and enhancement of existing diagnostic tools already available to the GP to support their clinical decision making. Other randomised controlled trials of interventions in primary care have taken this approach,(34) including the feasibility trial of the oesophago-gastric eRAT.(14, 35, 36) To promote patient awareness of the practice's participation in the ERICA trial, including requesting practices to add it to their websites and any social media feed. A selection of patients will be recruited to the nested process evaluation and health economics studies (see below and Appendices B and D).

Randomisation

Practices will be randomised using a 1:1 ratio into one of two trial arms: usual diagnostic care (control) and usual diagnostic practice plus access to the suite of eRATs, as the intervention. Randomisation will be computer-generated and web-based, conducted by an independent member of staff at the Exeter Clinical Trials Unit (ExeCTU), overseen by the CTU statistician (not the trial statistician). To promote balance between the trial arms in practices' use of the 2-week wait system, and therefore propensity to refer to secondary care, we will minimise randomisation by age-sex standardised 2-week wait referral ratio (the best available proxy) in national tertiles. We will use simple randomisation to allocate the first 50 practices (~10% of the total target), and then apply minimisation by 2-week wait referral ratio tertile, taking into account the previous allocations to inform the minimisation algorithm. All allocations using the minimisation algorithm will retain a stochastic element, aimed at promoting allocation concealment.

For peer review only - http://bmjopen.bmj.com/site/about/guidelines.xhtml

The data analysis will be carried out by the trial statistician and health-economist, blinded to treatment allocation and all primary outcome data are objective assessments of clinical outcome. Staging (the primary outcome) will be performed by pathologists unaware of trial participation or allocation. However, given the nature of the intervention, it is not possible to blind GPs or the GP practice to treatment allocation.

Outcome measures

Primary outcome

Outcome measures will be captured at patient-level, using data routinely collected by the National Cancer Registration and Analysis Service (NCRAS). The primary outcome is whether a patient is diagnosed at stage 1 or 2 (early) or stage 3 or 4 (advanced). This division of staging is commonly used and is a targeted metric in the 2019 NHS Long Term Plan - for stage 1 and 2 cancers (for all staged cancers other than non-melanoma skin cancer) at diagnosis to comprise 75% of the total by 2028. The current UK overall incidence-weighted percentage of early stage at diagnosis was 55% in 2018, though for the six eRAT cancers, it is 35%.(37)

For peer review only

Secondary outcomes

A range of secondary outcomes will be examined:

1. The binary stage at diagnosis of a further six cancers without eRATs will be identified from NCRAS, and compared between intervention and control practices. This is to investigate the possibility of a 'spill-over' effect whereby eRATs are associated with increased diagnostic activity beyond the eRAT cancers.
2. The practice's number of patients diagnosed with the six eRAT cancers combined, and the total number of cancer cases, from NCRAS.
3. The number of patients investigated or referred under the 2-week wait system for the six eRAT cancers combined, and in total, from Cancer Waiting Times data.
4. Route to diagnosis from the Routes to Diagnosis Dataset,(18) which uses Hospital Episode Statistics data. This will be categorised into four possible routes: emergency attendance, 2-week wait referral, GP referral, and "other". We will collect this information for each of the six eRAT cancers, and for the six comparator non-eRAT cancers.
5. 2-week wait performance measures, from Cancer Waiting Times data, for the six eRAT cancers combined, and for all cancer referrals:

For peer review only - http://bmjopen.bmj.com/site/about/guidelines.xhtml

5.1 Whether a patient on a 2-week wait pathway received a diagnosis of cancer. When aggregated, for example at the practice-level, and expressed as the proportion of patients who received a cancer diagnosis, this is known as the conversion rate.

5.2 The duration between 2-week wait referral and diagnosis of cancer in days

5.3Whether patients referred on a 2-week wait referral and who received a cancer diagnosis were diagnosed within 28 days, the Faster Diagnosis Standard (introduced in 2022).

5.4 Detection rate – the proportion of a practice's cancers which are identified via the 2-week wait pathway.

6. Survival measures (from date of diagnosis): 30-day; 1-year (identified from NCRAS). 5-year survival will also be reported, but the main trial will report on 30 day and 1-year, with 5-year data being a subsidiary report. These outcomes will use all-cause mortality data from the Office for National Statistics.

7. Adverse events (using data from the Diagnostic Imaging Dataset): these are expected to be few, and largely related to complications from hospital investigation such as colonoscopy. There is no mechanism for adverse events to be collected using routine data. We will, however, estimate any change in the expected number of adverse events from imaging investigations (colonoscopies, sigmoidoscopies, upper gastro-intestinal endoscopies, chest x-rays, abdominal ultrasounds, and abdominal CT scans) through investigating any change in the rate of these investigations in intervention practices relative to control practices (see data analysis section). Potential adverse psychological consequences of being labelled with 'possible cancer' will be further explored in the process evaluation.

Data collection

All primary and secondary outcome measures are available from NCRAS, DID and publicly available practice level data, including Cancer Waiting Times data. We will be using depersonalised (pseudo-anonymised) data. The Public Health England Office for Data Release (ODR) guidelines indicated that no legal gateway (e.g., section 251 approval) will be necessary to obtain these data.

Data analysis

All analyses will follow CONSORT guidelines for cluster-randomised and pragmatic trials. The primary analysis, exploring the proportion of cancer patients with early stage at diagnosis, will use mixed-

For peer review only - http://bmjopen.bmj.com/site/about/guidelines.xhtml

effects logistic regression with a random intercept for practice to accommodate the hierarchical nature of the data (i.e. random allocation by practice, with participants nested within practice). This regression will include trial-arm at practice-level, and will adjust for patient-level covariates known to be associated with stage (age, sex, quintile of the income domain from the Index of Multiple Deprivation (IMD), and cancer site),(38) and the practice-level minimisation variable (national tertile of age-sex standardised two-week wait referral ratio). We will further adjust the model at the practice-level for list size, clinical IT system used, and Care Quality Commission (CQC) overall rating, should these variables be associated with stage in preliminary analyses (even if not unbalanced with respect to trial allocation). Trial arm and covariates will all be entered as fixed effects. The degree of change in the percentage of patients diagnosed at a late stage in intervention practices will be investigated by exploring the marginal distributions of trial arm on the probabilities predicted by these models.

For the secondary outcome of the stage at diagnosis of six cancers without eRATs, we will repeat the above model including data on the six non-eRAT cancers as well as the six eRAT cancers. This model will use all the variables described above, plus an indicator variable for whether the cancer site has an eRAT and an interaction term between this variable and trial arm. From this model, we will obtain odds ratios (with 95% CIs) for: (i) the "spill over" effect of having the intervention on cancer sites not included in the intervention, and (ii) for the relative effect of the intervention on stage for included cancer sites compared with those not included in the intervention.

Mixed-effects logistic regression models with a random intercept for practice will also be fitted for the other secondary binary outcomes; route to diagnosis, conversion rate, and timeliness. These models will include trial arm as a practice-level effect, and will adjust at the patient-level for age, sex, and quintile of the Index of Multiple Deprivation (IMD) income domain, and at the practice-level for the minimisation variable (national tertile of age-sex standardised two-week wait referral ratio). These analyses will also adjust at the patient-level for cancer site (routes to diagnosis analyses) or for referral type (2-week wait analyses) as appropriate. The models will be further adjusted as in the main outcome variable analysis.

Time-to-event secondary outcomes (length of waiting time, survival) will be analysed using mixed-effects parametric survival models with a random intercept for practice, and all other variables added as fixed effects. These models will include trial-arm as a practice-level effect, and will adjust for the same patient-level factors as described above (waiting times adjusted for referral pathway rather than cancer site as above), and the practice-level minimisation variable (national tertile of age-sex standardised 2-week wait referral ratio). The models will also use the same adjustment as the primary

For peer review only - http://bmjopen.bmj.com/site/about/guidelines.xhtml

outcome measure. An appropriate distribution to model the baseline hazard will be utilised, as determined by a comparison of the Akaike Information Criteria under different distributions.(39)

For rate outcomes (number of 2-week wait referrals, cancers, and imaging investigations), we will analyse the rates per 100,000 registered patients per year by age-sex strata using mixed-effects Poisson regression models including a random intercept for practice. These models will include trial-arm as a predictor and will adjust for the age and sex of the strata, and at the practice-level for the minimisation variable (2-week wait referral ratio) and deprivation (quintile of IMD overall score). The models will be further adjusted at the practice-level for list size, clinical IT system used, CQC overall rating, and for the age and sex case-mix of practices should these covariates be found to be associated with the outcome (even if not unbalanced with respect to allocation). Case-mix will be incorporated by including variables for counts of practice populations in different age-sex strata (5-year age groups by sex, excluding one age group-sex stratum that can be determined once all others are known).

All the above analyses will combine data for the six eRAT cancers for each model. For outcomes related to two-week wait referrals, data will be combined for all referral pathways relevant to the six eRAT cancers. To investigate whether the eRATs produce a "spill-over" effect, whereby diagnostic activity is increased for other cancers, we will repeat all analyses using data for the six non-eRAT cancers combined for each model. Investigation of a spill-over effect for 2-week wait referral outcomes will use data for all referral pathways combined.

Additional sensitivity analyses will be conducted for the primary outcome in order to explore moderation arising from practice-level characteristics, using interaction terms. Although the trial has not been powered to detect low to moderate subgroup differences, large interaction effects that differ with respect to the direction of effect across subgroups are of interest. The potential impact of missing staging data on the primary outcome will also be explored through use of multiple imputation methods making use of auxiliary variables such as survival time, morphology and grade to improve the Missing At Random (MAR) assumption in line with previous work).(40)

Data management

Cancer registry data (NCRAS) will be managed and prepared by the registry themselves and securely, electronically transferred to the study team. There will be no patient identifiable data within these datasets. Data from NCRAS will be stored on the Secure Data Resource Hub at the University of Exeter (which meets requirements for secure storage of sensitive data) and linked to existing practice data held within ExeCTU's REDCap database. The data will be stored and retained in accordance with registry policies.

For peer review only - http://bmjopen.bmj.com/site/about/guidelines.xhtml

The nested studies rely on identifying patients from in-practice usage reports. These reports contain depersonalised (pseudo-anonymised) data. The practice will send a copy to the trial team with the original practice ID number removed. The local at practice reports will be securely and electronically transferred to a secure Exeter CTU computer.

In the recruitment of patients (and NHS staff) for interviews, questionnaires, or permission for access to medical notes, participant details will be passed securely between NHS services and the research team. All participants agreeing to interview, to complete a questionnaire, and/or medical notes review, and all GPs agreeing to interview will be allocated a unique study ID, and the information linking their ID to their personal details will be kept securely at the University of Exeter. All other participant-related paper records will be anonymised and stored separately from the personal information. The electronic database for the trial will be stored on the secure servers of the University of Exeter with password-controlled access provided for the research team by ExeCTU. Single data entry with extensive in-built validity checks will be used to reduce the risk of transcription errors.

Audio recordings will be digitised, encrypted and stored on the University's secure server. Audio recordings will be retained until after anonymised transcripts have been finalised and analysed. At this stage they will be securely and permanently deleted. Access to personal data will be restricted to the research team. Names and participant details will not be passed to any third parties and no named individuals will be included in the outputs. All participants (patients, NHS staff) will be asked for their consent for the study team to retain interview transcripts for the purposes of future research by those involved directly in the study team or to be used for educational purposes.

Informatica Systems Ltd has developed a separate agreement ('Data processing deed') for intervention practices which will be used between the GP practices and Informatica Systems Ltd. The deed was necessary because the development of Skyline has impacted on the processing arrangements for the eRATs software that is used. The ERICA research study will still use the Organisation Information Document which outlines the research team's data processing requirements, to be signed between the practice and Sponsor.

All study data will be kept for 10 years (unless data registry policy requires otherwise) under secure conditions on University of Exeter secure servers. Data will also be subject to standard secure storage and usage policies.

For peer review only - http://bmjopen.bmj.com/site/about/guidelines.xhtml

Trial monitoring and management

Trial Sponsor and Funders

The University of Exeter is the trial sponsor. The trial funders are providing finance to run the trial. None of the funders or sponsor will be involved in the design or day-to-day conduct of the trial, analysis of data, or interpretation of findings.

Trial Steering Committee (with Data Monitoring Committee responsibilities)

The responsibilities of the Trial Steering Committee (TSC) will be to review the main study protocol and any amendments, monitor and supervise the trial towards its interim and overall objectives, review relevant information from other sources, and to help resolve problems brought by the Trial Management group (TMG). The TSC will therefore provide overall independent supervision for ERICA on behalf of the funders and the Sponsor. Meetings will be held at regular intervals determined by need and not less than twice a year. Routine business will be conducted by telephone, videoconference, and email. The TSC will also operate as a Data Monitoring Committee with responsibility to monitor the overall conduct of the trial. There will be a time lag between practices 'entering the trial' and data availability from cancer registries. The time lag will be such that data will only be available once practices have completed data collection. Therefore, interim analyses to assess whether the trial was effective, and to support a decision whether to stop the trial early, would be unnecessary as data collection (and practice participation) would have already ceased.

Trial Management Group

A TMG has been established and includes those responsible for the day-to-day management of the trial and those supporting the delivery of the trial and associated stakeholders, including representatives of the Local Clinical Research Networks (LCRN) and Macmillan. The group will monitor all aspects of the conduct and progress of the trial, ensure that the protocol is adhered to and take appropriate action to safeguard participants and the quality of the trial itself. The group will meet regularly (monthly in the first instance, until recruitment has completed) in person and/or by phone or over the internet (via MS Teams).

Core Study Team

The core study team (Chief investigator, Trial Manager (TM)) will meet weekly during the study. Day-to-day running of the trial will be the responsibility of the TM. The TM will have access to the ExeCTU suite of standard operating procedures (SOPs) and will ensure that the trial is run in compliance with all relevant SOPs (e.g., assessment, processes and reporting, data management, study staff health and safety).

For peer review only - http://bmjopen.bmj.com/site/about/guidelines.xhtml

Nested Studies

Health Economics

We will estimate the cost and cost-effectiveness of the eRATs versus usual diagnostic practice using the primary perspective of the NHS and Personal Social Services (i.e. third-party payer). We will estimate the cost-effectiveness of the intervention based upon the primary outcome and secondary survival outcomes (30-day and 1-year; 5-year survival will be a subsidiary report) for the six cancer sites with eRATs and report the results using the latest guidelines.(41) For three cancer sites we will use decision analytic models to combine data from the within-trial analysis of ERICA intervention on costs and benefits, with longer estimates derived from the evidence synthesis of the costs and benefits of stage of diagnosis and disease progression to estimate the cost per Quality Adjusted Life Year (QALY) over the longer term.(42) For fuller details see Appendix B.

Service Delivery Modelling

This will investigate the key factors central to the (re) organisation of NHS diagnostic services for cancer referrals. We will use a range of methods, both quantitative and qualitative, to analyse service delivery alternatives. Specifically, we will aim to use modelling approaches to explore the likely implications of different scenarios across dimensions of performance, outcomes and costs. Fuller details are in Appendix C.

Process Evaluation

The process evaluation work aims to identify and investigate the contextual factors that impact upon the effectiveness of the eRATs with a particular focus on intervention fidelity and GP engagement. The impact of the eRATs on the patients' experience of their GP consultation and their experiences of subsequent care will also be explored. Fuller details are in Appendix D.

GP Workload

This nested study aims to explore, in terms of consultation time, the impact of GPs using eRATs on GP workload and patient 'flow' through consulting sessions. It will also explore workload in the week following the index consultation in which an eRAT was activated. Fuller details are in Appendix E.

Patient and Public Involvement and Engagement

Our Patient and Public Involvement and Engagement (PPIE) group, including cancer survivors, have been consulted widely during the development of this study. The PPIE group have reviewed and

For peer review only - http://bmjopen.bmj.com/site/about/guidelines.xhtml

commented on the protocol and supported the development of all patient-facing materials including information sheets and study lay summaries. One experienced PPIE representative sits on the TMG and another is on the TSC. A total of seven people have joined our PPIE group for this study and will contribute by reviewing study materials and documentation, commenting upon and proof reading reports and contributing to dissemination activities. This group will be supported in their work by the South West Peninsula Applied Research Collaboration (PenARC) PPIE team, for example by attending workshops on critical appraisal skills. All PPIE representatives will be recompensed for their time given to the study.

Ethics and Dissemination

A trial publication policy will be developed which outlines the plan for dissemination and will be in accordance with the International Committee of Medical Journal Editors. The results of the trial will be reported first to study collaborators and to the funder. The main report will be drafted by the TMG and circulated to all collaborators and the TSC for comment.

Access to the final trial datasets will be made publicly available unless contractual agreements between data providers limit such access.

Ethical review

The trial has received favourable Ethical review from London City & East Research Ethics committee, reference number 19/LO/0615, with eight amendments between then and 2022, relating to three main areas: the delays caused by the COVID-19 pandemic, with its recruitment moratorium; an alteration in the mechanism by which the eRATs software were delivered; and the inclusion of a nested study focussing on the impact of eRATs on GP workload. Current protocol version – V 6.0, 8th August, 2022.

Author contributions: WH conceived of the trial. Substantial contributions to the design of the methods and research processes were made by WH, JC, LM, SD, GA, MP, AS, AML, FW, EF, ES, MS, AM and RC. The protocol was written by RC, LM, SD, GA, AS, EF, and MP under the overall editorial control of WH. All authors critically reviewed the protocol and provided approval of the final version.

Funding statement: This research is funded by a philanthropic donation by The Dennis and Mireille Gillings Foundation, Cancer Research UK (C8640/A23385), plus support from Macmillan in provision of staff time, and the University of Exeter. The trial is registered with ISRCTN: (trial no: ISRCTN22560297) and on the CRUK trial registry (CRUK database no: 16163).

For peer review only - http://bmjopen.bmj.com/site/about/guidelines.xhtml

Acknowledgements: We would like to thank the NIHR Clinical Research Network for their support with recruitment, Macmillan for their contributions to the early eRAT work and ongoing support with practice recruitment and pilot testing. SD's time is partially supported by the National Institute of Health Research Applied Research Collaboration (ARC) South-West Peninsula.

Disclaimer: The views expressed are those of the authors and not necessarily those of the NHS, the NIHR or Department of Health.

Competing interests statement: WH has intellectual property rights to the original RATs, though has never sought to commercialize these. All other authors: no competing interests to declare.

For peer review only

For peer review only - http://bmjopen.bmj.com/site/about/guidelines.xhtml

References

1. Richards MA. The size of the prize for earlier diagnosis of cancer in England. Br J Cancer. 2009;101.
2. Richards M, Thorlby R, Fisher R, Turton C. Unfinished business: An assessment of the national approach to improving cancer services in England 1995–2015. London; 2018.
3. NHS England. (2019) The NHS Long Term Plan [Available from: https://www.longtermplan.nhs.uk/.
4. de Koning HJ, van der Aalst CM, de Jong PA, Scholten ET, Nackaerts K, Heuvelmans MA, et al. Reduced Lung-Cancer Mortality with Volume CT Screening in a Randomized Trial. New England Journal of Medicine. 2020;382(6):503-13.
5. Richards M. THE INDEPENDENT REVIEW OF ADULT SCREENING PROGRAMMES. NHS England; 2019. Contract No.: 01089.
6. Landy R, Pesola F, Castanon A, Sasieni P. Impact of cervical screening on cervical cancer mortality: estimation using stage-specific results from a nested case-control study. Br J Cancer. 2016;115(9):1140-6.
7. Abel GA, Mendonca SC, McPhail S, Zhou Y, Elliss-Brookes L, Lyratzopoulos G. Emergency diagnosis of cancer and previous general practice consultations: insights from linked patient survey data. British Journal of General Practice. 2017.
8. Hamilton W, Walter FM, Rubin G, Neal RD. Improving early diagnosis of symptomatic cancer. Nature Reviews Clinical Oncology. 2016;13:740.
9. Hamilton W, Peters TJ, Round A, Sharp D. What are the clinical features of lung cancer before the diagnosis is made? A population based case-control study. Thorax. 2005;60(12):1059-65.
10. NICE. Suspected cancer: recognition and referral [NG12] 2015 [Available from: https://www.nice.org.uk/guidance/ng12.
11. Hamilton W, Green T, Martins T, Elliott K, Rubin G, Macleod U. Evaluation of risk assessment tools for suspected cancer in general practice: a cohort study. British Journal of General Practice. 2013;63(606):e30-e6.
12. Price S, Spencer A, Medina-Lara A, Hamilton W. Availability and use of cancer decision-support tools: a cross-sectional survey of UK primary care. British Journal of General Practice. 2019;69(684):e437.
13. Medina-Lara A, Grigore B, Lewis R, Peters J, Price S, Landa P, et al. Cancer diagnostic tools to aid decision-making in primary care: mixed-methods systematic reviews and cost-effectiveness analysis. Health technology assessment (Winchester, England). 2020;24:66.
14. Rubin G, Walter FM, Emery J, Hamilton W, Hoare Z, Howse J, et al. Electronic clinical decision support tool for assessing stomach symptoms in primary care (ECASS): a feasibility study. BMJ Open. 2021;11(3):e041795.
15. Kostopoulou O, Arora K, Pálfi B. Using cancer risk algorithms to improve risk estimates and referral decisions. Communications Medicine. 2022;2(1):2.
16. Hamilton W. Diagnosing symptomatic cancer in the NHS. BMJ : British Medical Journal. 2015;351:h5311.
17. Møller H, Gildea C, Meechan D, Rubin G, Round T, Vedsted P. Use of the English urgent referral pathway for suspected cancer and mortality in patients with cancer: cohort study. BMJ. 2015;351.
18. Elliss-Brookes L, McPhail S, Ives A, Greenslade M, Shelton J, Hiom S. Routes to diagnosis for cancer - determining the patient journey using multiple routine data sets. Br J Cancer. 2012;107.
19. Hamilton W. The CAPER studies: five case-control studies aimed at identifying and quantifying the risk of cancer in symptomatic primary care patients. Br J Cancer. 2009;101 Suppl 2:S80-6.
20. Hamilton W, Peters TJ, Bankhead C, Sharp D. Risk of ovarian cancer in women with symptoms in primary care: population based case-control study. BMJ. 2009;339:b2998.

For peer review only - http://bmjopen.bmj.com/site/about/guidelines.xhtml

21. Hamilton W, Round A, Sharp D, Peters TJ. Clinical features of colorectal cancer before diagnosis: a population-based case-control study. Br J Cancer. 2005;93(4):399-405.
22. Shephard E, Neal R, Rose P, Walter F, Hamilton WT. Clinical features of kidney cancer in primary care: a case-control study using primary care records. The British journal of general practice : the journal of the Royal College of General Practitioners. 2013;63(609):e250-5.
23. Shephard EA, Stapley S, Neal RD, Rose P, Walter FM, Hamilton WT. Clinical features of bladder cancer in primary care. The British journal of general practice : the journal of the Royal College of General Practitioners. 2012;62(602):e598-604.
24. Stapley S, Peters TJ, Neal RD, Rose PW, Walter FM, Hamilton W. The risk of oesophago-gastric cancer in symptomatic patients in primary care: a large case-control study using electronic records. Br J Cancer. 2013;108(1):25-31.
25. NICE. Suspected cancer: recognition and referral [NG12]2015. Available from: http://www.nice.org.uk/guidance/NG12.
26. Macmillan. (2015) Rapid referral guidelines [available at: https://www.macmillan.org.uk/_images/rapid-referral-toolkit-desktop_tcm9-291864.pdf].
27. Cancer Research UK. (2017) Cancer Stats 2017 [Available from: http://www.cancerresearchuk.org/health-professional/cancer-statistics].
28. Collins GS, Altman DG. Identifying patients with undetected gastro-oesophageal cancer in primary care: External validation of QCancer(R) (Gastro-Oesophageal). European journal of cancer (Oxford, England : 1990). 2013;49(5):1040-8.
29. Hamilton W, Stapley S, Campbell C, Lyratzopoulos G, Rubin G, Neal R. For which cancers might patients benefit most from expedited symptomatic diagnosis? Construction of a ranking order by a modified Delphi technique. BMC Cancer. 2015;15:820.
30. McPhail S, Johnson S, Greenberg D, Peake M, Rous B. Stage at diagnosis and early mortality from cancer in England. Br J Cancer. 2015;112(s1):S108-S15.
31. Shephard EA, Neal RD, Rose PW, Walter FM, Hamilton W. Symptoms of adult chronic and acute leukaemia before diagnosis: large primary care case-control studies using electronic records. British Journal of General Practice. 2016;66(644):e182-e8.
32. UK CR. Statistics by Cancer Type 2022 [Available from: https://www.cancerresearchuk.org/health-professional/cancer-statistics/statistics-by-cancer-type.
33. Barclay ME, Lyratzopoulos G, Greenberg DC, Abel GA. Missing data and chance variation in public reporting of cancer stage at diagnosis: Cross-sectional analysis of population-based data in England. Cancer Epidemiology. 2018;52:28-42.
34. Campbell JL, Fletcher E, Britten N, Green C, Holt TA, Lattimer V, et al. Telephone triage for management of same-day consultation requests in general practice (the ESTEEM trial): a cluster-randomised controlled trial and cost-consequence analysis. The Lancet. 2014;384(9957):1859-68.
35. Downs M, Turner S, Bryans M, Wilcock J, Keady J, Levin E, et al. Effectiveness of educational interventions in improving detection and management of dementia in primary care: cluster randomised controlled study. BMJ. 2006;332(7543):692.
36. Flottorp S, Oxman AD, Håvelsrud K, Treweek S, Herrin J. Cluster randomised controlled trial of tailored interventions to improve the management of urinary tract infections in women and sore throat. Bmj. 2002;325(7360):367.
37. National Disease Registration Service:. Case-mix adjusted percentage of cancers diagnosed at stages 1 and 2 in England, by Clinical Commissioning Group2020 25 Jan 2022. Available from: https://www.gov.uk/government/statistics/case-mix-adjusted-percentage-cancers-diagnosed-at-stages-1-and-2-by-ccg-in-england/national-disease-registration-service-case-mix-adjusted-percentage-of-cancers-diagnosed-at-stages-1-and-2-in-england-by-clinical-commissioning-group.
38. Lyratzopoulos G, Abel GA, Brown CH, Rous BA, Vernon SA, Roland M, et al. Socio-demographic inequalities in stage of cancer diagnosis: evidence from patients with female breast, lung, colon, rectal, prostate, renal, bladder, melanoma, ovarian and endometrial cancer. Annals of Oncology. 2012;24(3):843-50.
39. Bozdogan H. Model selection and Akaike's Information Criterion (AIC): The general theory and its analytical extensions. Psychometrika. 1987;52(3):345-70.

For peer review only - http://bmjopen.bmj.com/site/about/guidelines.xhtml

40. Lyratzopoulos G, Abel GA, Brown CH, Rous BA, Vernon SA, Roland M, et al. Socio-demographic inequalities in stage of cancer diagnosis: evidence from patients with female breast, lung, colon, rectal, prostate, renal, bladder, melanoma, ovarian and endometrial cancer. Ann Oncol. 2013;24(3):843-50.
41. Husereau D, Drummond M, Augustovski F, de Bekker-Grob E, Briggs AH, Carswell C, et al. Consolidated Health Economic Evaluation Reporting Standards 2022 (CHEERS 2022) statement: updated reporting guidance for health economic evaluations. BMJ. 2022;376:e067975.
42. Weinstein MC, O'Brien B, Hornberger J, Jackson J, Johannesson M, McCabe C, et al. Principles of Good Practice for Decision Analytic Modeling in Health-Care Evaluation: Report of the ISPOR Task Force on Good Research Practices—Modeling Studies. Value in Health. 2003;6(1):9-17.

For peer review only

For peer review only - http://bmjopen.bmj.com/site/about/guidelines.xhtml

A pragmatic cluster randomised controlled trial assessing the clinical effectiveness and cost-effectiveness of electronic risk-assessment for cancer for patients in general practice (ERICA): Appendices

For peer review only

Appendix A. Managing practice splits and mergers in analyses

Although we will exclude practices that report imminent restructuring during recruitment, there may be unforeseen mergers or splits of practices. Where mergers and splits are concerned, this could mean, for example, that some of our practices who were in the control arm may merge with an intervention practice. Similarly, a non-trial practice may become part of a trial practice (intervention or control). Changes in practice size have implications on the denominator – the number of patients that each practice is likely to be contributing to our sample – and is a particular issue for three of our secondary outcome measures based on rates (cancer diagnosis rate, two-week wait referral rate, and adverse event rate). Importantly, however, this issue is not a problem for our primary outcome of staging.

We define a split and mergers as follows: Split – Where a population of patients registered to a single practice with a single practice code become registered with two or more individual practices with different practice codes. The practice codes of the new practices may be new codes (i.e. did not exist prior to the split) or one may inherit the original practice code (although this is not a requirement). The change in registration of patients must occur to a substantial number of patients and not at their request. Merger – Where a population of patients registered to one or more practices with different practice codes become registered at a single individual practice with a single practice code. The practice code of the new practice may be a new code (i.e., did not exist prior to the split) or it may inherit one of the original practice codes. A federation is not a “merger” in these terms.

Excluding practices who restructure during the trial may unnecessarily reduce our power. Therefore, we will try and accommodate changes in status. The Table outlines our approach. The assumption is that the change takes place at time T. Any practice which splits goes from X to Y and Z, and mergers are Z plus Y becoming X. Intervention practices are I, and comparison practices C.

Practice size fluctuations will be monitored in real time. Practice size data are freely and publicly available from NHS Digital and are updated monthly. Each month during the data collection, the trial statistician will download the practice size data and inspect size for all the practices in the trial (the statistician will remain blinded to outcome allocation). If the practice size differs by more than 10% the statistician will alert the trial manager, who will contact the research champion in the relevant practice to explore the reasons for this practice size change. Reasons (e.g., mergers, splits) will be recorded.

For peer review only

Table: managing changes in practice size – mergers and splits

<table>
<tr><th>Split or merger</th><th>X pre change</th><th>Y pre change</th><th>Z pre change</th><th>X post change</th><th>Y post</th><th>Z post</th></tr>
<tr><td rowspan="2">Split</td><td>I</td><td></td><td></td><td></td><td>I</td><td>I</td></tr>
<tr><td>C</td><td></td><td></td><td></td><td>C</td><td>C</td></tr>
<tr><td colspan="7">We will allow the daughter practices to withdraw from the trial if they desire, which would mean we lose Y or Z (or both). If daughter practices decide to withdraw, we will include data up to time T plus 2 months to allow for average diagnostic time to cancer.</td></tr>
<tr><td rowspan="5">Merger</td><td></td><td>I</td><td>I</td><td>I</td><td></td><td></td></tr>
<tr><td></td><td>I</td><td>C</td><td>I</td><td rowspan="2" colspan="2">There is likely to be wash over under these conditions, so the merged practice will be considered as I</td></tr>
<tr><td></td><td>I</td><td>Non-trial</td><td>I</td></tr>
<tr><td></td><td>C</td><td>Non-trial</td><td>C</td><td></td><td></td></tr>
<tr><td></td><td>C</td><td>C</td><td>C</td><td colspan="2"></td></tr>
</table>

For peer review only

We will manage changes in practice size at the data analysis stage of the trial. Where changes in list size of more than 10% within a month are seen, data for that practice will not be included in the analysis of rate outcomes from one month prior to the change. There are two exceptions to this; 1) splits where all the daughter practices remain in the trial and we continue to treat them as a single practice for rate analyses, 2) mergers where merged practices are in the same arm of the trial, and we will analyse them as a single practice from the start for rate analyses.

Appendix B

Health Economics

Intervention costings. The resources used in developing the training materials and videos (preparation and IT support) will be collected from the trial manager; nationally applicable unit costs will be applied. Estimates of the extent to which these videos are watched by practice staff will be based on information available from the website platform hosting the videos. Information on the resources use to install the eRATs onto the EMIS and SystmOne practice IT systems will be estimated in consultation with practice champions. These estimates will additionally aim to estimate: 1) the cost of installation in the trial and 2) the anticipated cost of future installation should eRATs be implemented nationally.

Health related quality of life and resource use. The Health Economics analysis will draw on the estimated number of imaging investigations (colonoscopies, sigmoidoscopies, upper gastro-intestinal endoscopies, chest x-rays, abdominal ultrasounds, and abdominal CT scans) using data from the Diagnostic Imaging Dataset available in the main trial as well as estimates of GP workload from the process evaluation. Practices will be offered remuneration of nearly £200 for the additional work.

To investigate whether the eRATs intervention was associated with a change in health-related quality of life using the EQ5D-5L and to provide more detailed information on primary care services and tests used, we will sample patients in the intervention arm who had a consultation where an eRAT alert occurred, and patients in the control arm who had a consultation where an eRAT alert would have occurred. We will strategically target practices in both trial arms who have either high, medium, or low two-week wait referral rates, matching the minimisation criteria in the main trial. It is anticipated that 15-20 patients per practice over a 2-week period will have a consultation with an eRAT alert. All patients who have an eRAT alert will be invited to complete a baseline questionnaire and a 3 month follow-up Health Economics questionnaire, as will equivalent patients in the control arm. We anticipate that 40% of patients will accept, and of these there will be 20% who do not respond. With a conservative estimate of a cluster size of five patients responding to the questionnaire, plususing an minimum clinically important difference of 0.1 for the EQ5D-5L (2) and a standard deviation of 0.23(3), with an inter-cluster correlation coefficient of 0.03 (4), and an estimated coefficient of variation of cluster size of 0.7, the sample size required to detect a between group difference with 90% power and alpha of 0.05 was 28 clusters (140 participants) per arm. Participants who agree to take part will receive the questionnaire as a hard copy, through the post, or electronically via email, depending on the participant's preference. Nationally applicable unit costs will be used for all community health and social care contacts (5) and secondary care services, tests and investigations will be costed using the National Schedule of Reference Costs 2016-2017. (6)

For peer review only

Decision Analytic Model

The modelling aims to predict the expected impact of a change in stage of diagnosis, and any resulting change in the distribution of cancer stage at diagnosis (intervention vs. control) over time, building on the published literature in this area.(7-10) The decision analytic models will not need to separately model the diagnostic phase, and we will take the trial's primary outcomes, stage at diagnosis (Stage 1-4 separately and not collated into Stage 1-2 and Stage 3-4), to model the longer term effects on survival, QALYs and secondary care costs.

Scenario analysis will be used to examine the impact on the results of multiple parameters changing simultaneously (based on *a priori* judgement about the combination of parameters to include).(11) Probabilistic sensitivity analysis will be used to explore the proportion of results that are considered cost-effective in relation to a given cost-effectiveness threshold and these results will be illustrated graphically using a cost-effectiveness acceptability curve.(12)

The study will follow the CHEERs guidelines for reporting cost-effectiveness studies and models,(13) and will discount both costs and outcomes at 3.5% as recommended by the National Institute of Health and Care Excellence.(14) Sensitivity analyses will examine alternative assumptions about the missing data mechanisms.(15)

Service Evaluation

We will draw upon published systematic reviews of Quality of Life measures, that are based on public preferences and measured in patients (as required by NICE guidelines (16) and that have been used for economic evaluation modelling studies.(17)

Appendix C

Service Delivery Modelling

Background and rationale

Cancer diagnosis has become one of the principal areas of focus and concern for the NHS in England.(18) For some time, NHS performance in both early diagnosis, delays in referral, and associated survival rates has been poor relative to our national aspirations and when compared with other first world countries. This has worsened during the COVID pandemic. In this context, many of the issues of concern are centred on key aspects of service delivery. How the NHS organises its services is often pivotal in determining the cost, feasibility, and effectiveness. For instance, factors such as workforce availability, prioritisation, service location, scale, and resources are fundamental to the performance of the NHS in delivering effective cancer services.

This component of the ERICA programme will investigate the key factors central to the organisation of NHS diagnostic services for cancer referrals. We will use a range of methods, both quantitative and qualitative, to analyse service delivery alternatives. Specifically, we will aim to build an economic model to assess the likely implications of different scenarios. Implementation of the eRAT diagnostic tool at primary care level is likely to impact directly on the follow-on pathway for cancer diagnosis (for example in terms of the volume and case mix of referred patients for diagnosis). Our model will

For peer review only

therefore provide an assessment of the likely effect of this impact in terms of costs and performance, and highlight any changes in organisation that might be implied by the introduction of the eRAT tool.

This research will run in parallel with the substantive work conducted for the controlled trial of eRAT implementation within ERICA. It will also liaise closely with the detailed and standard analysis of cost-effectiveness for disease progression (which is inherently abstracted from the service delivery aspects of care) in order to provide an added dimension to the cost-effectiveness outputs from the ERICA study as a whole.

Objectives

To build and populate a model of the cancer diagnostic pathway for England, in order to provide an assessment of the costs and effectiveness of different scenarios for service delivery. In particular, we will investigate the potential aspects relative to implementation of eRATs based on the study data collected from the ERICA trial. In addition, qualitative research with NHS staff in secondary care will be used to assess key areas central to successful implementation and sustainability.

Methods

A wide range of methods will be essential to fulfil the objectives of the work outlined here. Early work will include a literature search and survey of current systems for diagnostics in cancer. We will therefore conduct a systematic review of the related literature in the field and carry out a survey of current service delivery organisation across a range of settings. This work will aim to identify the key factors bearing on the organisation of services such a regional variation, metropolitan versus rural context, and population case mix differences.

Phase two work will aim to build a model in order to capture the key elements of service delivery for diagnostic services for cancer. This will explore a range of modelling approaches and test which is most suited to specific needs. For example, discrete event simulation, Systems Dynamics, geographic analysis, and Markov modelling will all be tested in terms of their relevance and appropriateness to specific requirements. In this context it is highly likely that different modelling tools will be relevant to the diverse needs of the study, so no single approach will be dominant.

Phase three will focus on the service delivery implications for the introduction of the eRAT diagnostic tool in primary care looking particularly at the potential knock-on effects in other areas of service.

In addition to our modelling work, we will use qualitative methods, such as problem structuring methods, soft systems mapping, to provide an assessment of some key elements of implementation.

For peer review only

Data

A wide range of data will be used to complete this component of the work. We will aim to integrate sources from across routinely collected datasets such as those listed below to construct our models: NHS activity data, Waiting time data, Reference cost data, Diagnostic Imaging Data (DIDs), Hospital Episode Statistics (HES), Workforce reference data, GP and hospital referral data, QOF data, Population data (e.g. ONS). In addition, we will aim to incorporate the primary data derived from the main ERICA study in order to model and assess the pathway impact from the use of eRATs. We will also use the outputs from the standard economic analysis as an input for the cost effectiveness of the service delivery modelling. Output from the qualitative research will also provide important data for informing the outputs of this work, for example in feeding into the recommendations and conclusions of the study.

Appendix D

Process Evaluation

Scope of process evaluation

The process evaluation work aims to identify and investigate the contextual factors that impact upon the effectiveness of the eRATs with a particular focus on intervention fidelity and GP engagement. The impact of the eRATs on the patients' experience of their GP consultation and their experiences of subsequent care will also be explored. It is underpinned by the COM-B framework for understanding behaviour change (19). This framework will outline the interactive nature of how the GP's capability (IT skill for using the eRATs), opportunity (eRAT prompts), and motivation (to do the training and use all the eRAT features) might influence their behaviour – i.e. ongoing use of the eRATs, symptom checker, coding of symptoms and changes to referral letters. We will use a mixed-methods approach to explore how the intervention was delivered (including fidelity and dose - if the eRATs were being used as intended and their degree of use across intervention practices and over time) and GP engagement with and acceptability of using the eRATs (GP's experiences of the eRATs).(20) For delivery, we will be particularly interested in fidelity of function. (21) GPs will be given clear training videos on how to use the eRATs and we will explore the extent to which GPs engaged with training as well as how they subsequently engaged with the software, and the GP's experiences of how it impacted on the GP-patient relationship in order to evaluate how they responded to the intervention.

Methods

Intervention fidelity and GP engagement (intervention arm only): Prior to the start of the intervention GPs require a minimum level of training in how to use the eRATs. Although the software is designed to be intuitive, a clinical system specific walkthrough for the two main functions of the eRATs (prompt and symptom checker) and FAQs will be available via separate videos. The research champion will be

For peer review only

given access to the videos and can disseminate the video content to all GPs in the practice (by showing the videos during a practice meeting, providing a demonstration themselves, or passing on the weblink). Once practices have started the data collection phase, we will invite up to 10 research champions to interview to discuss in depth their experiences of the set-up and training procedures and to explore whether their GPs have the capability, opportunity and motivation to use the eRATs. We will purposively sample research champions based on whether they are from a practice with a high, middle or low two-week wait referral rate, which software system their practice uses, their gender, and their level of experience in practice (10+ years vs. less than 10 years in practice).

Detailed eRAT usage can be captured for all IT systems. Usage will be captured in two ways – i) via a central log and ii) via local 'at practice' reports. For i), usage logs will be routinely and automatically sent from the practice to the Informatica 'digital warehouse' and will contain anonymised, practice-level data for each eRAT including reports of: how many times the prompt was shown, how many times the symptom checker was used, the number of times the symptoms were changed during use of the symptom checker, the length of time the symptom checker was open for, and whether clinical guidance was accessed from the eRAT. These centrally reported logs will be available on a monthly basis throughout the course of the trial and will be securely sent from Informatica to the research team who will add the data to the trial database.

For ii), usage will be examined via reports run locally at each practice. These reports include individual patient level data outlining which eRAT was triggered, the patient's risk score on the eRAT, when the symptom checker was opened and closed, patient's age and sex, and a list of possible eRAT symptoms and whether they were changed. These reports contain depersonalised (pseudo-anonymised) data. As it is possible to potentially identify the patient via the practice ID number we will ask practices to make a copy of the report, add in a new patient study ID variable (e.g., p1, p2, p3, etc) and save it to the practice computer. We will then ask them to send a copy to the trial tram with the original practice ID number removed. They will also send the file with a predetermined practice ID number. These measures should ensure the data is anonymised. The local at practice reports will be securely and electronically transferred to a secure Exeter CTU computer.

Intervention fidelity (Intervention and control). We will ask all research champions in the intervention practice to complete a short questionnaire (online via a secure, University approved provider) detailing their experience of installing software, using the eRATs, and whether alternative risk tools have also been used. We will ask research champions at control practices their experiences of being in the trial and whether they have started using any cancer risk tools. The questionnaires will be

For peer review only

completed at two time points – i) within 12 months of the start of the intervention; ii) at the end of the data collection period.

For identifying GPs to interview, we will use maximum variation purposive sampling (sampling on practice two-week wait referral rate (high vs. medium vs. low); software system used, gender, length of time in practice (10+years vs. < 10 years), and working status (part time vs full time)) and expect to interview up to 18 GPs from intervention practices to ask them about their experience of the eRATs including the training provided, any impacts on the consultation and their clinical decision making, as well as any changes in symptom coding behaviour. We will invite GPs to interview after the intervention has been running for at least 3 months. Written information will be provided about the interview study and written consent will be taken prior to the interview and will be verbally confirmed before the interview commences. Interviews will be audio-recorded and carried out by telephone, face-to-face (only if it is safe to do so), or over the internet (e.g., Zoom or MS Teams) depending on the GP's preference, by members of the research team using a pre-defined topic guide that focuses on their training and capability to use eRATs, their opportunity to use the eRATS over the study period and their motivation to continue using the system. If a face-to-face interview is chosen (and safe to perform), interviews will take place in a private room at the practice. The researcher will comply with the lone worker policy, ensuring that have a 'buddy' within the research team monitoring their activities and whereabouts. The interviews may raise sensitive issues such as workload and GP overburden or burnout: the interview study information sheet will provide appropriate sources for accessing confidential support. GPs will be reminded that they have the right to not answer any question, stop the interview or withdraw from the interview study; if there is insufficient time to fully discuss issues GPs will be offered a follow-up time to complete the interview.

GP coding behaviour: It is possible that the eRATs will impact GP coding behaviour - GP coding behaviour for cancer specific symptoms may increase; this would cause a minor increase in triggering of eRATs. We will explore the impact of eRATs on coding behaviour in the interviews (see above) and, resources permitting, will also examine the impact on coding rates using the following approach. We will purposively sample 12 intervention practices and 12 control practices in the South/South West of England based on two-week wait referral rate (i.e., 4 low, 4 moderate, 4 high referring practices) and which software system is being used. In the first instance we will invite practices who are participating in the nested study to support this work. If insufficient numbers agree, we will approach other practices who are not participating in the nested studies. We will explore the rate of coding of the most frequent symptom for each eRAT cancer in the study that underpins that particular cancer (e.g. cough, abdominal pain, haematuria)(22-25) for a month in the first three months of entry into ERICA, and for the same calendar month a year and two years later (as some symptoms have seasonal

For peer review only

variation). This will be performed retrospectively, by the search code being given to the research champion, who will arrange for the search to be conducted in the practice. The results of the search will be emailed to the research team.

Patient experience of care: We will adopt a phased, targeted recruitment strategy with an aim to purposively sample up to (based on two-week rate referral rate (low vs. medium vs. high); gender, age (40-60 vs. 60+)) 32 patients from the intervention arm. We will approach five practices at a time (and expect to recruit around 20 practices to reach the target number of participants), to ensure that we can interview participants in a timely manner.

The in-practice eRAT reports are the mechanism by which we will be able to identify individuals to invite to participate in the activities associated with the process evaluation. The local (at practice) reporting mechanism will allow the research team to identify individuals for whom the eRATs were used and thus who are potentially eligible to participate in a semi-structured interview. Purposive sampling will take place – practices will hold the master eRAT report containing both the patients practice ID number and the new patient study ID. The research team will let the practice know the patient study IDs for those whom an invitation letter will be sent. Practices will be offered remuneration of nearly £200 for the additional work.

Via the GP practice, the research team will send out a letter and information booklet to the identified patients to invite participation in an interview to discuss their experience of care. We will adopt a longitudinal case study design (26) – patients' care pathways will differ, some will receive referrals into secondary care for investigations and tests, while some will be on a 'watch and wait' plan, revisiting their GP at an agreed interval. Some patients will have tests for cancer and the test will indicate that there is no cancer (false positives) whereas some patients will be diagnosed with cancer. So that we can fully capture all patient groups at different stages of their care, individuals will be invited for repeat interviews at regular intervals (i.e., at least one month apart and no more than 3 interviews within 12 months).

We aim to perform the first interview within one month of the consultation in which an eRAT was triggered. Written information about the interview study will have been provided and written informed consent will be taken prior to all interviews, and will be verbally confirmed before the interview commences. Interviews will be audio-recorded and carried out by members of the research team using pre-defined topic guides. The initial interview will be conducted face-to-face at the participant's home or via video conferencing software such as MS Teams at a time convenient for the participant, with any subsequent interview conducted either face-to-face, over the phone, or via video

For peer review only

conference software, depending upon the participant's preference. We will monitor the progression of the Covid-19 pandemic and fully adhere to government advice around social distancing and travel. We will not put the research team or participants at risk and will primarily conduct interviews online. If it is safe to conduct face-to-face interviews, the researcher will comply with the lone worker policy, ensuring that have a 'buddy' within the research team monitoring their activities, whereabouts and expected completion time. The interviews may raise anxiety or concerns related to uncertainty about diagnosis during the referral and investigation period or the watch and wait period; or psychological distress associated with a cancer diagnosis or a false-positive result. The interview study information sheet will provide appropriate sources for accessing confidential support and patients will be reminded that they have the right to not answer any question, stop the interview or withdraw from the interview study.

Management of adverse consequences

As a result of being referred for tests or investigations there is a risk of an adverse incident. If referral rates do increase as a result of access to eRATs, there is an increased risk of an adverse event (AE) to patients of practices allocated to the intervention. We are not routinely tracking individuals throughout the trial and there is no mechanism for monitoring any AEs as a result of referral. However, psychological distress may be a consequence of referral. Individuals for whom cancer is diagnosed at an early stage may be relieved by the diagnosis and see the psychological distress as justifiable. Individuals for whom a referral does not lead to a diagnosis of cancer (false positives) may have undergone unnecessary psychological distress. Our process evaluation work will help us to understand the extent of this and its potential impact on the individuals' life.

During interviews, patients may report being distressed – either as a result of research activity or as a result of their health, and events in their private lives. Should such a situation arise, the researchers will implement the trial risk protocol and manage the participant in accordance with this policy. Participants will be reminded that they have the right to not answer any question, stop the interview or withdraw from the interview study. Under high-risk situations (e.g. where there is perceived immediate risk to a participant's health), the study team may be required to break confidentiality, to inform appropriate authorities who will need to provide essential care services. We will also signpost participants to sources of support. This information will be outlined in the Participant Information Sheet. Participants will be informed of possible benefits and known risks of participation in the interviews by means of a Patient Information Sheet and through discussion with the research team. Written consent will be obtained immediately prior to the interview study.

For peer review only

There are minimal risks to researchers as most interviews will take place in the GP practices or by telephone/online; however, if a home visit is undertaken to interview patient participants the researcher will follow the lone worker policy: researchers will make sure that their whereabouts, contact telephone number and estimated time of return are known to their colleagues and/or manager. Researchers will also have the opportunity to debrief with a senior colleague on the research team should they need any support after conducting an interview; this debrief may be in person or by telephone.

Analysis

For the quantitative results the individual data sources will be summarised descriptively, including a summary of data completeness. For the qualitative data we will adopt a framework approach (27) which allows the inclusion of key concepts and ideas identified from the literature, alongside themes emerging from the data. The framework approach produces a structured output matrix, with cells of data organised by practice and by code (a descriptive label applied to a section of transcript).

At least two researchers will work on the analysis. Interviews will be audio recorded, transcribed and anonymised. Data familiarisation will be achieved through the listening to and reading of interview recordings and transcripts. Transcripts will be imported into the qualitative data analysis software package NVivo 11 (QSR International) to facilitate data management, sharing and development of a coding framework. A proportion of the interview transcripts will be coded by each researcher. The 'constant comparative method' (28) will be utilised: each incident in the data will be compared with other incidents for similarities and differences and any 'negative cases', where a case does not fit the pattern or cannot be explained by the emerging analysis, will be explored and recorded. Following this initial coding, a PPIE meeting (one for the GP interviews and one for the patient interviews) will be held to discuss the emerging themes from the interviews, and to gain alternative perspectives from the PPIE group on those themes. Following the PPIE meeting, the analytical framework will be developed, incorporating researcher and PPIE perspectives on the results, with a final set of themes and codes being agreed upon.

The analytical framework will be applied to all interview transcripts; one researcher will index all transcripts, with a second researcher indexing a proportion, to check the reliability of the indexing and to ensure that the themes of the framework are being interpreted consistently. Any differences in interpretation will be discussed between the two researchers. Following the indexing process, data will be charted into the structured output matrix, which will summarise the data on each theme from all transcripts. A subsequent meeting of the PPIE group will be held once all of the results from the process evaluation have been gathered to gain a users' perspective of the global findings.

For peer review only

The final step in the process evaluation analysis will be to integrate results from the various mixed method data sources using a triangulation protocol(29) to give a more complete picture once individual data sources have been individually analysed. We plan to create a summary matrix, known as a convergence coding matrix, which summarises the findings from each data source after assessing whether the findings are in agreement, partial agreement or no agreement, or whether the data source is silent for the finding under consideration i.e. when a theme or finding arises from one data set but not another.

Reporting

The process evaluation results will be briefly summarised for inclusion in the main trial report and publication, separate dissemination (reports, presentations and publications) will provide further details of the process evaluation findings.

Appendix E

GP Workload

Background and rationale

GPs manage a high and rising workload of increasingly complex patient care with many competing demands to attend to within ten-minute consultations. (30) This, combined with ongoing recruitment and retention challenges, has contributed to a GP workforce 'crisis'. (31-36) The workload implications for GPs of using electronic tools such as eRATs during consultations is unclear.(37) ERICA provides an opportunity to examine whether the use of eRATs by GPs, and the possible subsequent discussion of cancer risk with patients, may impact consultation length and patient 'flow' through consulting sessions. This nested study aims to explore, in terms of consultation time, the impact of GPs using eRATs on GP workload and patient 'flow' through consulting sessions. It will also explore workload in the week following the index consultation in which an eRAT was activated, when relevant letters may be generated, referrals made, investigations followed through, or clinical discussions engaged in.

Objectives

The specific objectives in respect of consultations and sessions are:

(i) to measure and compare the duration of consultations and sessions in which an eRAT has been activated with consultations where eRATs have not been activated;

For peer review only

(ii) to measure and compare the duration of subsequent consultations in the same session after an eRAT has been activated with consultations in sessions where eRATs have not been activated;

(iii) to explore the frequency of interactions with patients' medical records by a GP in the week following a consultation during which an eRAT was activated.

Methods

An observational quantitative study will be conducted in a sub-sample of ERICA intervention practices to examine the durations of consultations and consulting sessions in which eRATs are activated.

Sample size

The basis for the sub-study sample size calculation is on the number of consultations likely to occur over a two-week period within ERICA practices, in which an eRAT will be 'activated' (i.e. an eRAT prompt is shown and/or clinician uses an eRAT symptom checker). A number of assumptions are of note:

The first assumption is that a half-day GP consulting session, typically lasting four hours and comprised of ten-minute consultations, would be associated with a total of 24 consultations. Second, practices have an average headcount of seven GPs (informed by GP workforce data from NHS Digital). (38) Third, a GP is assumed to work an average of 6.7 half-day consulting sessions per week. (39) An average practice would therefore provide a total of 1,126 GP consultations per week.

Accurate estimations of how often an eRAT will be activated, are not yet established in previous research on usage of cancer decision tools in UK general practice. (40,41) Two clinical members of the research team have estimated that an eRAT may be expected to be activated once per GP, per week. This estimate would suggest that approximately 15% of consulting sessions will involve a consultation where the eRAT tool was activated.

The standard deviation for both the length of a consultation and of a whole consulting session from previous literature was four minutes and 20 minutes respectively. (42-44) Project team discussion concluded that a minimally important difference in time for an individual consultation would be between two and five minutes; for a consulting session this minimally important difference would be approximately 10 minutes.

Statistical power to detect a time difference of between two and five minutes in eRAT consultations versus non-eRAT consultations is also in excess of >80%, even if eRATs are observed to have been activated in just 1:40 consulting (2.5% of sessions), the basis of the most conservative estimate. The

For peer review only

power to detect a difference of 10 minutes in sessions where eRATs have been activated compared with sessions where eRATs have not been activated is >80%, even if eRATs affect only 2.5% of sessions. A two-week observation period would provide sufficient data and power to detect differences in the length of consultations and sessions where an eRAT is activated and those where an eRAT is not activated.

Outcome measures

Primary outcome

The primary outcome is the length of time (in minutes) of consultations. These will be consultations during which an eRAT is activated and also those during which an eRAT is not activated. For the purposes of this sub-study, a consultation is defined as starting when the patient's electronic medical record is opened by a GP, for the purpose of conducting either a face-to-face or telephone/video interaction with the patient, and ending when the record is closed. Home visits will be excluded due to difficulty in accessing accurate time information. Consultations with health professionals who would not make referral decisions (e.g. practice nurses, physiotherapists, pharmacists, healthcare assistants) will also be excluded.

Secondary outcomes

In addition to our primary outcome, we propose to examine the following secondary outcomes:

- The length of time (in minutes) of consulting sessions. For the purposes of this study, a session is defined as a half-day period comprised of individual patients' pre-booked or same-day consultations. The half-day periods are typically 'morning' or 'afternoon', although some practices offer early morning and evening sessions as well. (45)
- The number of instances of opening a patient's electronic medical record in the week following an eRAT being activated.

Practice recruitment

An initial pilot in up to three ERICA intervention practices will be undertaken and plans for data collection methods revisited at that point. Practices will be approached by an invitation email and provided with an information sheet detailing the nature of the study and providing contact details of the researcher. No individual patients will be recruited.

A note on practice recruitment to the nested studies: We expect to recruit up to 91 practices across the nested studies (56 in the health economics nested study, up to 20 in the process evaluation and up to 15 in the sub-study on GP workload) practices. Practices will only be asked to help with one of the health economic nested study, the process evaluation nested study, or the GP workload sub-study.

For peer review only

Data collection

Identifying consultations where an eRAT is activated

The Process Evaluation describes earlier how a local 'at practice' report will be run for practices in order to collect patient-level data on eRATs usage. This report will be run for practices recruited to this nested study, covering a two-week period.

Measuring durations of consultations and sessions

The eRATs usage report will provide the start and end time of the tool usage, but not the duration of a consultation. A further search function (developed within SystmOne for this nested study) will provide data on the timings of all consultations occurring between two dates (referred to as the 'appointments report'). The consultations identified in the eRAT usage report will be cross-referenced with the consultations in the appointments report. A variable will be added to denote which consultations involved an eRAT being activated and which did not.

Measuring workload in the week following an eRAT being activated

The eRATs usage report will identify the relevant patient records for which an audit will be run in SystmOne. The audit will provide data on instances of the records being opened and closed by practice staff during the week following the index activation of an eRAT.

Data analysis

Data will be analysed in Stata. Descriptive statistics summarising participating practices and GPs will be presented. Although practice level data will be presented, it will be anonymised (e.g. practice A, B, etc) to protect the identities of individual practitioners or practices.

The primary analysis of the durations of consultations in which an eRAT is activated, will take the form of a mixed-effects linear regression with random intercepts to account for clustering within GPs and for GPs clustering within practices. This regression will adjust for consulting GP, time of day, day of week, and consultation mode (face-to-face, telephone, video). Residuals will be checked for normality. As duration data are typically not normally distributed, the data will be transformed if needed, using log transformation. Bootstrapping of the data will also be undertaken if needed. Similar mixed-effects linear regression models with random intercepts will also be performed for secondary outcomes; the duration of consulting sessions, and the number of instances of opening a patient's electronic medical record in the week following an eRAT being activated. For all models where duration is the outcome linear models will be used, but for the count of opening medical records Poisson models will be used.

For peer review only

Governance and ethical considerations

Consent

Individual patient consent is not sought within ERICA for the running of the eRAT usage report. The reports in SystmOne, described for this nested study, will not contain identifiable patient data nor clinically sensitive information and so patient consent for these reports will also not be sought.

Data protection/management and confidentiality

The eRAT usage report and the SystmOne reports will contain pseudo-anonymised data: a patient identifier. However, the reports will contain variables denoting date, time and consulting GP, which will allow cross-referencing, so practices will be asked to delete the patient identifier before sending the report securely and electronically to a secure Exeter CTU computer using a predetermined practice ID number. These measures will ensure the data are anonymised. In the event that the researcher visits the practice to run the SystmOne reports, the files will be anonymised in the same way before the researcher leaves. Practices will keep the original 'master' report files containing the patient's practice computer ID.

Finance

The additional work for the nested study, outside of ERICA costs, is for practices to run the reports in SystmOne and send the report files securely to the researcher. Alternatively, the researcher will visit the practice to run the reports, which may require time of a practice administrator or manager for logging in to the clinical system and orientation. In both scenarios, this time would be covered by nested study research costs at a rate of £50 per hour, and each practice will be offered reimbursement for up to 2 hours. Travel costs for the researcher to visit practices where needed are estimated at £0.45 per mile for a 75 mile round-trip per practice (South West).

For peer review only

Appendix F. A simplified schema illustrating the pathways to a cancer diagnosis in the UK. The size of the arrow reflects the approximate proportion of cancers taking each route. The yellow central arrow represents where eRATs are expected to have an effect.

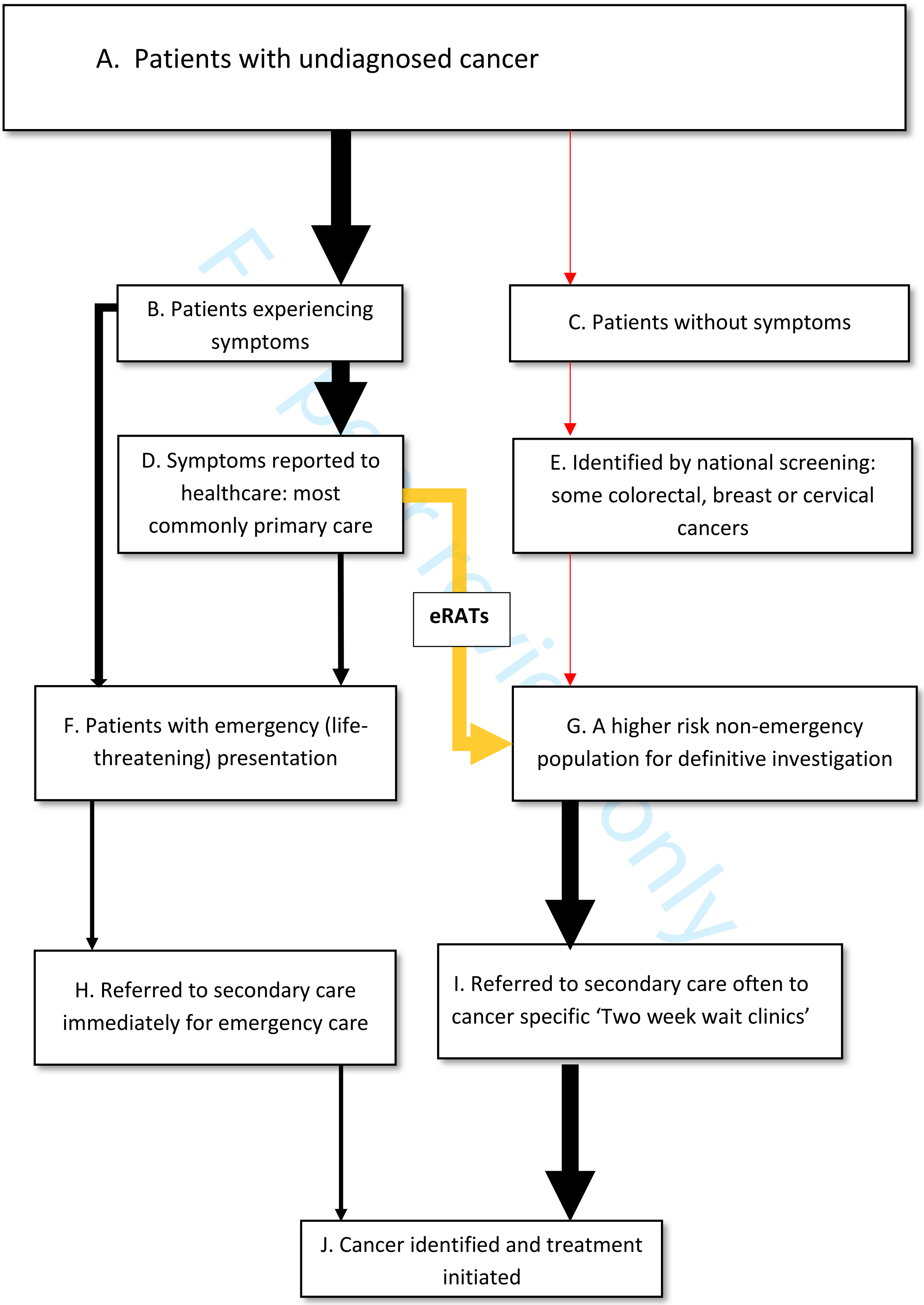

References

1. Neal RD, Din NU, Hamilton W, Ukoumunne OC, Carter B, Stapley S, et al. Comparison of cancer diagnostic intervals before and after implementation of NICE guidelines: analysis of data from the UK General Practice Research Database. Br J Cancer. 2014;110(3):584-92.
2. Pickard AS, Neary MP, Cella D. Estimation of minimally important differences in EQ-5D utility and VAS scores in cancer. Health and quality of life outcomes. 2007;5:70-.
3. Kind P, Hardman G, Macran S. UK population norms for EQ-5D. Centre for Health Economics, University of York; 1999.
4. Man M-S, Chaplin K, Mann C, Bower P, Brookes S, Fitzpatrick B, et al. Improving the management of multimorbidity in general practice: protocol of a cluster randomised controlled trial (The 3D Study). BMJ Open. 2016;6(4):e011261.
5. Curtis L. Unit Costs of Health and Social Care 2020 [Available from: https://www.pssru.ac.uk/project-pages/unit-costs/unit-costs-2020/]. 2020.
6. Department of Health and Social Care. NHS reference costs 2019 to 2020 [Available from: https://improvement.nhs.uk/resources/reference-costs/]. 2020.
7. Caro JJ, Briggs AH, Siebert U, Kuntz KM. Modeling good research practices--overview: a report of the ISPOR-SMDM Modeling Good Research Practices Task Force-1. Medical decision making : an international journal of the Society for Medical Decision Making. 2012;32(5):667-77.
8. Siebert U, Alagoz O, Bayoumi AM, Jahn B, Owens DK, Cohen DJ, et al. State-Transition Modeling:A Report of the ISPOR-SMDM Modeling Good Research Practices Task Force–3. Medical Decision Making. 2012;32(5):690-700.
9. Schwander B, Hiligsmann M, Nuijten M, Evers S. Systematic review and overview of health economic evaluation models in obesity prevention and therapy. Expert review of pharmacoeconomics & outcomes research. 2016;16(5):561-70.
10. Wang BC, Furnback W. Modelling the long-term outcomes of bariatric surgery: A review of cost-effectiveness studies. Best practice & research Clinical gastroenterology. 2013;27(6):987-95.
11. Andronis L, Barton P, Bryan S. Sensitivity analysis in economic evaluation: an audit of NICE current practice and a review of its use and value in decision-making. Health Technol Assess. 2009;13(29):iii, ix-xi, 1-61.
12. Fenwick E, O'Brien BJ, Briggs A. Cost-effectiveness acceptability curves--facts, fallacies and frequently asked questions. Health economics. 2004;13(5):405-15.
13. Husereau D, Drummond M, Petrou S, Carswell C, Moher D, Greenberg D, et al. Consolidated Health Economic Evaluation Reporting Standards (CHEERS) statement. Value Health. 2013;16(2):e1-5.
14. NICE. Guide to the Methods of Technology Appraisals, Section 5.6 Discounting [Available from: https://www.nice.org.uk/process/pmg9/chapter/the-reference-case#discounting]. 2013.
15. Faria R, Gomes M, Epstein D, White IR. A guide to handling missing data in cost-effectiveness analysis conducted within randomised controlled trials. PharmacoEconomics. 2014;32(12):1157-70.
16. Brazier J, Longworth L. NICE DSU Technical Support Document 8: An introduction to the measurement and valuation of health for NICE submissions. In: Health, editor. 2011.
17. Tengs TO, Wallace A. One thousand health-related quality-of-life estimates. Medical care. 2000;38(6):583-637.
18. NHS England. (2019) The NHS Long Term Plan [Available from: https://www.longtermplan.nhs.uk/.
19. Michie S, van Stralen MM, West R. The behaviour change wheel: a new method for characterising and designing behaviour change interventions. Implementation science : IS. 2011;6:42-.
20. Grant A, Treweek S, Dreischulte T, Foy R, Guthrie B. Process evaluations for cluster-randomised trials of complex interventions: a proposed framework for design and reporting. Trials. 2013;14:15.
21. Hawe P, Shiell A, Riley T. Complex interventions: how "out of control" can a randomised controlled trial be? BMJ (Clinical research ed). 2004;328(7455):1561-3.
22. Shephard EA, Stapley S, Neal RD, Rose P, Walter FM, Hamilton WT. Clinical features of bladder cancer in primary care. The British journal of general practice : the journal of the Royal College of General Practitioners. 2012;62(602):e598-604.
23. Shephard E, Neal R, Rose P, Walter F, Hamilton WT. Clinical features of kidney cancer in primary care: a case-control study using primary care records. The British journal of general practice : the journal of the Royal College of General Practitioners. 2013;63(609):e250-5.

For peer review only

24. Hamilton W. The CAPER studies: five case-control studies aimed at identifying and quantifying the risk of cancer in symptomatic primary care patients. Br J Cancer. 2009;101 Suppl 2:S80-6.
25. Stapley S, Peters TJ, Neal RD, Rose PW, Walter FM, Hamilton W. The risk of oesophago-gastric cancer in symptomatic patients in primary care: a large case-control study using electronic records. Br J Cancer. 2013;108(1):25-31.
26. Yin R. Case Study Research: Design and Methods. London: Sage Publications 2003.
27. Ritchie J, Spencer L, O'Connor W. Carrying out Qualitative Analysis. In: Ritchie J, Lewis J, editors. Qualitative Research Practice, A guide for Social Science Students and Researchers London: Sage; 2003.
28. Corbin J, Strauss A. Basics of qualitative research: Techniques and procedures for developing grounded theory. London: Sage publications; 2014.
29. O'Cathain A, Murphy E, Nicholl J. Three techniques for integrating data in mixed methods studies. BMJ. 2010;341:c4587.
30. Hobbs FDR, Bankhead C, Mukhtar T, Stevens S, Perera-Salazar R, Holt T, et al. Clinical workload in UK primary care: a retrospective analysis of 100 million consultations in England, 2007-14. Lancet 2016;387:2323-30. http://dx.doi.org/10.1016/S0140-6736(16)00620-6
31. Baird B, Charles A, Honeyman M, Maguire D, Das P. Understanding pressures in general practice: The Kings Fund; 2016.
32. Campbell J CR, Sansom A. Retaining the experienced GP workforce in direct patient care (ReGROUP): final report. Exeter: University of Exeter Medical School; 2015.
33. Sansom A, Terry R, Fletcher E, Salisbury C, Long L, Richards SH, et al. Why do GPs leave direct patient care and what might help to retain them? A qualitative study of GPs in South West England. BMJ Open 2018;8:e019849. http://dx.doi.org/10.1136/bmjopen-2017-019849
34. Roland M, Everington S. Tackling the crisis in general practice. BMJ 2016;352:i942. http://dx.doi.org/10.1136/bmj.i942
35. Fletcher E, Abel GA, Anderson R, Richards SH, Salisbury C, Dean SG, et al. Quitting patient care and career break intentions among general practitioners in South West England: findings of a census survey of general practitioners. BMJ Open 2017;7:e015853. http://dx.doi.org/10.1136/bmjopen-2017-015853
36. Walker B, Moss, C., Gibson, J., Sutton, M., Spooner, S., Checkland, K. Tenth National GP Worklife Survey. Manchester: Policy Research Unit in Commissioning and the Healthcare System Manchester Centre for Health Economics; 2019.
37. Fletcher E, Burns, A., Wiering, B., Lavi, D., Shephard, E., Hamilton, W., Campbell, J. L., Abel, G. Workload and workflow implications associated with the use of electronic risk assessment tools used by health professionals in general practice: a scoping review. In: University of Exeter Medical School; 2022.
38. NHS Digital. General Practice Workforce 30 September 2019. NHS Digital; 2019. URL: https://digital.nhs.uk/data-and-information/publications/statistical/general-and-personal-medical-services/final-30-september-2019#resources (Accessed 11/02/20).
39. Gibson J, Sutton M, Spooner S, Checkland K. Ninth National GP Worklife Survey 2017. In; 2017.
40. Moffat J, Ironmonger L. Clinical Decision Support Tool for Cancer (CDS) Project evaluation report to the Department of Health; 2014.
41. Robinson R, Poirier V, Watson S. Using Cancer Decision Support Tools to support the early diagnosis of cancer; 2017.
42. Salisbury C, Procter S, Stewart K, Bowen L, Purdy S, Ridd M, et al. The content of general practice consultations: cross-sectional study based on video recordings. British Journal of General Practice 2013;63:e751. http://dx.doi.org/10.3399/bjgp13X674431
43. Elmore N, Burt J, Abel G, Maratos FA, Montague J, Campbell J, et al. Investigating the relationship between consultation length and patient experience: a cross-sectional study in primary care. Br J Gen Pract 2016;66:e896-e903. http://dx.doi.org/10.3399/bjgp16X687733
44. Orton PK, Pereira Gray D. Factors influencing consultation length in general/family practice. Fam Pract 2016;33:529-34. http://dx.doi.org/10.1093/fampra/cmw056
45. NHS. Working life (GP). NHS; 2022. URL: https://www.healthcareers.nhs.uk/explore-roles/doctors/roles-doctors/general-practice-gp/working-life (Accessed 2022).

For peer review only

STANDARD PROTOCOL ITEMS: RECOMMENDATIONS FOR INTERVENTIONAL TRIALS

SPIRIT 2013 Checklist: Recommended items to address in a clinical trial protocol and related documents*

Section/item	Item No	Description	Addressed on page number
**Administrative information**			
Title	1	Descriptive title identifying the study design, population, interventions, and, if applicable, trial acronym	Title page
Trial registration	2a	Trial identifier and registry name. If not yet registered, name of intended registry	1 & 15
	2b	All items from the World Health Organization Trial Registration Data Set	n/a
Protocol version	3	Date and version identifier	1 & 15
Funding	4	Sources and types of financial, material, and other support	15
Roles and responsibilities	5a	Names, affiliations, and roles of protocol contributors	15
	5b	Name and contact information for the trial sponsor	1
	5c	Role of study sponsor and funders, if any, in study design; collection, management, analysis, and interpretation of data; writing of the report; and the decision to submit the report for publication, including whether they will have ultimate authority over any of these activities	12
	5d	Composition, roles, and responsibilities of the coordinating centre, steering committee, endpoint adjudication committee, data management team, and other individuals or groups overseeing the trial, if applicable (see Item 21a for data monitoring committee)	12-13

For peer review only

Introduction

Background and rationale	6a	Description of research question and justification for undertaking the trial, including summary of relevant studies (published and unpublished) examining benefits and harms for each intervention	2-3
	6b	Explanation for choice of comparators	5-7
Objectives	7	Specific objectives or hypotheses	3-4
Trial design	8	Description of trial design including type of trial (eg, parallel group, crossover, factorial, single group), allocation ratio, and framework (eg, superiority, equivalence, noninferiority, exploratory)	4

Methods: Participants, interventions, and outcomes

Study setting	9	Description of study settings (eg, community clinic, academic hospital) and list of countries where data will be collected. Reference to where list of study sites can be obtained	4
Eligibility criteria	10	Inclusion and exclusion criteria for participants. If applicable, eligibility criteria for study centres and individuals who will perform the interventions (eg, surgeons, psychotherapists)	7
Interventions	11a	Interventions for each group with sufficient detail to allow replication, including how and when they will be administered	4-5
	11b	Criteria for discontinuing or modifying allocated interventions for a given trial participant (eg, drug dose change in response to harms, participant request, or improving/worsening disease)	n/a
	11c	Strategies to improve adherence to intervention protocols, and any procedures for monitoring adherence (eg, drug tablet return, laboratory tests)	14 and Appx D
	11d	Relevant concomitant care and interventions that are permitted or prohibited during the trial	4-7
Outcomes	12	Primary, secondary, and other outcomes, including the specific measurement variable (eg, systolic blood pressure), analysis metric (eg, change from baseline, final value, time to event), method of aggregation (eg, median, proportion), and time point for each outcome. Explanation of the clinical relevance of chosen efficacy and harm outcomes is strongly recommended	8-9
Participant timeline	13	Time schedule of enrolment, interventions (including any run-ins and washouts), assessments, and visits for participants. A schematic diagram is highly recommended (see Figure)	6

For peer review only - http://bmjopen.bmj.com/site/about/guidelines.xhtml

Sample size	14	Estimated number of participants needed to achieve study objectives and how it was determined, including clinical and statistical assumptions supporting any sample size calculations	6-7
Recruitment	15	Strategies for achieving adequate participant enrolment to reach target sample size	7
**Methods: Assignment of interventions (for controlled trials)**			
Allocation:			
Sequence generation	16a	Method of generating the allocation sequence (eg, computer-generated random numbers), and list of any factors for stratification. To reduce predictability of a random sequence, details of any planned restriction (eg, blocking) should be provided in a separate document that is unavailable to those who enrol participants or assign interventions	7
Allocation concealment mechanism	16b	Mechanism of implementing the allocation sequence (eg, central telephone; sequentially numbered, opaque, sealed envelopes), describing any steps to conceal the sequence until interventions are assigned	7
Implementation	16c	Who will generate the allocation sequence, who will enrol participants, and who will assign participants to interventions	7
Blinding (masking)	17a	Who will be blinded after assignment to interventions (eg, trial participants, care providers, outcome assessors, data analysts), and how	7-8
	17b	If blinded, circumstances under which unblinding is permissible, and procedure for revealing a participant’s allocated intervention during the trial	n/a
**Methods: Data collection, management, and analysis**			
Data collection methods	18a	Plans for assessment and collection of outcome, baseline, and other trial data, including any related processes to promote data quality (eg, duplicate measurements, training of assessors) and a description of study instruments (eg, questionnaires, laboratory tests) along with their reliability and validity, if known. Reference to where data collection forms can be found, if not in the protocol	9-12
	18b	Plans to promote participant retention and complete follow-up, including list of any outcome data to be collected for participants who discontinue or deviate from intervention protocols	11-12

For peer review only - http://bmjopen.bmj.com/site/about/guidelines.xhtml

Data management	19	Plans for data entry, coding, security, and storage, including any related processes to promote data quality (eg, double data entry; range checks for data values). Reference to where details of data management procedures can be found, if not in the protocol	11-12
Statistical methods	20a	Statistical methods for analysing primary and secondary outcomes. Reference to where other details of the statistical analysis plan can be found, if not in the protocol	9-11
	20b	Methods for any additional analyses (eg, subgroup and adjusted analyses)	11
	20c	Definition of analysis population relating to protocol non-adherence (eg, as randomised analysis), and any statistical methods to handle missing data (eg, multiple imputation)	11
**Methods: Monitoring**			
Data monitoring	21a	Composition of data monitoring committee (DMC); summary of its role and reporting structure; statement of whether it is independent from the sponsor and competing interests; and reference to where further details about its charter can be found, if not in the protocol. Alternatively, an explanation of why a DMC is not needed	12-13
	21b	Description of any interim analyses and stopping guidelines, including who will have access to these interim results and make the final decision to terminate the trial	n/a
Harms	22	Plans for collecting, assessing, reporting, and managing solicited and spontaneously reported adverse events and other unintended effects of trial interventions or trial conduct	9, Appendix D
Auditing	23	Frequency and procedures for auditing trial conduct, if any, and whether the process will be independent from investigators and the sponsor	n/a
**Ethics and dissemination**			
Research ethics approval	24	Plans for seeking research ethics committee/institutional review board (REC/IRB) approval	15
Protocol amendments	25	Plans for communicating important protocol modifications (eg, changes to eligibility criteria, outcomes, analyses) to relevant parties (eg, investigators, REC/IRBs, trial participants, trial registries, journals, regulators)	12-13

For peer review only - http://bmjopen.bmj.com/site/about/guidelines.xhtml

Consent or assent	26a	Who will obtain informed consent or assent from potential trial participants or authorised surrogates, and how (see Item 32)	11 & Appendices B & D (only relevant for nested studies)
	26b	Additional consent provisions for collection and use of participant data and biological specimens in ancillary studies, if applicable	11 & Appendices B & D (only relevant for nested studies)
Confidentiality	27	How personal information about potential and enrolled participants will be collected, shared, and maintained in order to protect confidentiality before, during, and after the trial	11-12
Declaration of interests	28	Financial and other competing interests for principal investigators for the overall trial and each study site	15
Access to data	29	Statement of who will have access to the final trial dataset, and disclosure of contractual agreements that limit such access for investigators	14
Ancillary and post-trial care	30	Provisions, if any, for ancillary and post-trial care, and for compensation to those who suffer harm from trial participation	n/a
Dissemination policy	31a	Plans for investigators and sponsor to communicate trial results to participants, healthcare professionals, the public, and other relevant groups (eg, via publication, reporting in results databases, or other data sharing arrangements), including any publication restrictions	14
	31b	Authorship eligibility guidelines and any intended use of professional writers	14
	31c	Plans, if any, for granting public access to the full protocol, participant-level dataset, and statistical code	14
**Appendices**			
Informed consent materials	32	Model consent form and other related documentation given to participants and authorised surrogates	Not applicable for main trial. Multiple documents for each nested study, available from authors upon request

For peer review only - http://bmjopen.bmj.com/site/about/guidelines.xhtml

Biological specimens	33	Plans for collection, laboratory evaluation, and storage of biological specimens for genetic or molecular analysis in the current trial and for future use in ancillary studies, if applicable	n/a

*It is strongly recommended that this checklist be read in conjunction with the SPIRIT 2013 Explanation & Elaboration for important clarification on the items. Amendments to the protocol should be tracked and dated. The SPIRIT checklist is copyrighted by the SPIRIT Group under the Creative Commons "Attribution-NonCommercial-NoDerivs 3.0 Unported" license.

For peer review only

For peer review only - http://bmjopen.bmj.com/site/about/guidelines.xhtml

BMJ Open

Protocol for a pragmatic cluster randomised controlled trial assessing the clinical effectiveness and cost-effectiveness of electronic risk-assessment for cancer for patients in general practice (ERICA)

Journal:	*BMJ Open*
Manuscript ID	bmjopen-2022-065232.R2
Article Type:	Protocol
Date Submitted by the Author:	27-Feb-2023

Complete List of Authors:	Hamilton, Willie; University of Exeter, Primary Care Diagnostics<br>Mounce, Luke; University of Exeter, Institute of Health Research<br>Abel, Gary; University of Exeter, University of Exeter Medical School (Primary Care)<br>Dean, Sarah; PenCLAHRC University of Exeter Medical School,<br>Campbell, John; University of Exeter, Primary Care<br>Warren, Fiona; University of Exeter Medical School, Institute of Health Research<br>Spencer, Anne; University of Exeter Medical School, Health Economics<br>Medina-Lara, Antonieta; University of Exeter Medical School, Health Economics Group<br>Pitt, Martin; University of Exeter, University of Exeter: Medical School<br>Shephard, Elizabeth; University of Exeter,<br>Shakespeare, Marijke; University of Exeter Medical School, Primary Care<br>Fletcher, Emily; University of Exeter Medical School, Primary Care Research Group<br>Mercer, Adrian; University of Exeter Medical School, Primary Care<br>Calitri, Raff; University of Exeter Medical School, Primary Care

**Primary Subject Heading**:	General practice / Family practice
Secondary Subject Heading:	Health services research
Keywords:	HEALTH SERVICES ADMINISTRATION & MANAGEMENT, Risk management < HEALTH SERVICES ADMINISTRATION & MANAGEMENT, PRIMARY CARE, Clinical trials < THERAPEUTICS

SCHOLARONE™
Manuscripts

Protocol for a pragmatic cluster randomised controlled trial assessing the clinical effectiveness and cost-effectiveness of electronic risk-assessment for cancer for patients in general practice (ERICA)

Willie Hamilton,[1] Luke Mounce, [1] Gary Abel, [1] Sarah Dean,[1] John Campbell, [1] Fiona Warren,[1] Anne Spencer, [1] Antonieta Medina-Lara, Martin Pitt, [1] Elizabeth Shephard,[1] Marijke Shakespeare, [1] Emily Fletcher, [1] Adrian Mercer,[1] Raff Calitri.[1]

[1]University of Exeter, St Luke's campus, Exeter, EX1 2LU

Willie Hamilton, Professor of Primary Care Diagnostics: w.hamilton@exeter.ac.uk, ORCID ID 0000-0003-1611-1373 (Corresponding author)

Luke Mounce, Research Fellow: l.t.a.mounce@exeter.ac.uk, ORCID ID 0000-0002-6089-0661

Gary Abel, Associate Professor: g.a.abel@exeter.ac.uk, ORCID ID 0000-0003-2231-5161

Sarah G Dean, Professor in Psychology Applied to Rehabilitation and Health: s.dean@exeter.ac.uk, ORCID ID 0000-0002-3682-5149

John Campbell, Professor of General Practice and Primary Care: john.compbell@exeter.ac.uk, ORCID ID 0000-0002-6752-3493

Fiona Warren, Senior Lecturer in Medical Statistics: f.c.warren@exeter.ac.uk, ORCID ID 0000-0002-3833-0182

Anne Spencer, Associate Professor: a.e.spencer@exeter..ac.uk ORCID ID 0000-0002-8163-3103

Antonieta Medina-Lara, Associate Professor in Health Economics: a.medina-lara@exeter.ac.uk, ORCID ID 0000-0001-7325-8246

Martin Pitt, Professor of Applied Healthcare Modelling and Data Science: m.pitt@exeter.ac.uk

Elizabeth Shephard, Research Fellow: e.a.shephard@exeter.ac.uk, ORCID ID 0000-0002-3610-3680

Marijke Shakespeare, Trial coordinator: m.shakespeare@exeter.ac.uk

Emily Fletcher, Research Fellow: e.fletcher@exeter.ac.uk, ORCID ID 0000-0003-1319-3051

Adrian Mercer, PPIE Representative, zante256@gmail.com

Raff Calitri, Research Fellow and Trial Manager, r.calitri@exeter.ac.uk, ORCID ID 0000-0003-0889-4670

For peer review only

Abstract

Introduction. The UK has worse cancer outcomes than most comparable countries, with a large contribution attributed to diagnostic delay. Electronic risk assessment tools (eRATs) have been developed to identify primary care patients with a ≥2% risk of cancer using features recorded in the electronic record.

Methods and analysis. This is a pragmatic cluster randomised controlled trial in English primary care. Individual general practices will be randomised in a 1:1 ratio to intervention (provision of eRATs for six common cancer sites) or to usual care. The primary outcome is cancer stage at diagnosis, dichotomised to stage 1 or 2 (early) or stage 3 or 4 (advanced) for these six cancers, assessed from national cancer registry data. Secondary outcomes include stage at diagnosis for a further six cancers without eRATs, use of urgent referral cancer pathways, total practice cancer diagnoses, routes to cancer diagnosis, and 30-day and 1-year cancer survival. Economic and process evaluations will be performed along with service delivery modelling. The primary analysis explores the proportion of cancer patients with early stage at diagnosis. The sample size calculation used an odds ratio of 0.8 for a cancer being diagnosed at advanced stage in the intervention arm compared with the control arm, equating to an absolute reduction of 4.8% as an incidence-weighted figure across the six cancers. This requires 530 practices overall, with the intervention active from April 2022 for 2 years.

Ethics and dissemination. The trial has approval from London City & East Research Ethics committee, reference number 19/LO/0615; protocol version 5.0, 9th May 2022. It is sponsored by the University of Exeter. Dissemination will be by journal publication, conferences, use of appropriate social media and direct sharing with cancer policymakers.

Registration. The trial is registered with ISRCTN: (trial no: ISRCTN22560297).

Word Count: 5665

Key words: Early cancer diagnosis, randomised controlled trial, clinical risk-assessment tools, General Practice

For peer review only - http://bmjopen.bmj.com/site/about/guidelines.xhtml

Article summary

Strengths and limitations of this study

- Improvements in primary care are seen as a key for improving early cancer diagnosis in the UK, and this trial is targeting that part of the diagnostic pathway.
- This is a large, definitive trial, powered to identify a clinically important difference in cancer stage at diagnosis.
- The trial is designed to minimise impact on participating practices with outcome data being obtained from routinely collected National Health Service data.
- One limitation is that the UK's national imperative to improve cancer diagnosis after the COVID pandemic may mean use of other interventions (or eRATs themselves) are encouraged by policymakers, reducing the validity and reliability of the trial.

For peer review only

Introduction

An estimated 10,000 UK cancer deaths each year would not occur if the UK matched the outcomes of other European countries.(1) Much of the difference is attributed to diagnostic delay.(2) The NHS Long Term plan, published in January 2019, specifically targets an increase in the percentage of cancer patients whose cancer is stage 1 or 2 (thus potentially curable) at diagnosis to rise from the current 54% to 75% by 2028.(3) Diagnosis of cancer may occur by several routes, but the main ones are population screening, and diagnosis after symptoms have occurred. Although screening for cancer is effective for colorectal, breast, lung and cervical cancers,(4-6) less than 10% of the total new UK cancers are identified by this route. Most of the remainder are diagnosed after presenting with symptoms, usually to primary care. Of patients with cancer, just under 20% present with an emergency complication of their cancer; however, many of these patients have previously reported symptoms attributable to their cancer to primary care, but this presentation did not lead to a diagnosis of cancer.(7) Within general practice, many studies have aimed at identifying the symptoms of possible cancer and quantifying their predictive value.(8) One main output has been Risk Assessment Tools (generally abbreviated to RATs); these give precise estimates of the chance of an underlying cancer as a percentage figure. RATs provide precise estimates for single symptoms (e.g. the risk of cancer of the lung for a person aged 40 years or more with haemoptysis is 2.4%), as pairs of symptoms (haemoptysis accompanied by loss of weight is 9.2%) or as repeated symptoms (a re-attendance with haemoptysis is 17%).(9) RATs are published for the 18 most common adult cancers, accounting for nearly 90% of the total cancer burden. These publications have been highly influential: in particular, they strongly contributed to the National Institute of Healthcare Excellence (NICE) guideline, Suspected cancer: recognition and referral [NG12], which guides symptomatic diagnosis of cancer in the UK.(10)

For peer review only - http://bmjopen.bmj.com/site/about/guidelines.xhtml

The initial RATs, of paper, mouse mat, calendar, or web-based forms, increased cancer diagnostic activity,(11) though impacts on hard outcomes such as stage at diagnosis or cancer survival were unknown. Electronic RATs (eRATs) for seven major cancers (lung, colorectal, pancreas, oesophago-gastric, bladder, kidney and ovary) have been developed for the two largest UK primary care electronic healthcare record systems, SystmOne and EMIS, used in around 80% of English practices. The software performs daily calculations of individual cancer risk in patients aged 40 and over, using coded symptoms and laboratory results in the patient's record over the past year, and prompts the general practitioner (GP) when the risk of one or more of these cancers is equal to or above 2%. Some form of electronic clinical decision support for cancer diagnosis has been downloaded by practices and used by at least one practice member in approximately 12% of English practices.(12). Two systematic reviews recently concluded that more research evidence was needed for impact on time to diagnosis and treatment, stage at diagnosis, and health outcomes, as well as research to understand how tools are used in GP consultations.(13) A feasibility trial of the oesophago-gastric eRAT published after these systematic reviews reported installation and regulatory problems that severely restricted usage,(14) and a vignette study of the colorectal RAT suggested it changed the GP's inclination to refer in 26% of usages.(15)

One crucial aspect of eRAT research relates to cost-effectiveness: annual NHS spending on cancer diagnosis is approximately £1bn.(16) Observational data showed increased use of the urgent cancer referral system to improve survival,(17) but there is insufficient data to inform a cost-effectiveness analysis of the subject.(13)

Objectives

The overarching aim of the trial is to assess the clinical and cost-effectiveness of using eRATs for six cancer sites – colorectal, lung, bladder, kidney, oesophago-gastric and ovarian cancers - compared with usual care for patients in general practice. Our hypothesis is that provision of eRATs will expedite the diagnosis of symptomatic cancer resulting in better cancer outcomes.

The primary objective is to compare the effects of using eRATs (vs usual care) on the percentage of patients with a newly diagnosed cancer at one of the six sites whose cancer is staged as being stage 1 or 2 (versus stage 3 or 4).

A secondary objective is to investigate differences in the stage at diagnosis of a further six cancers without eRATs (combined): breast, melanoma, prostate, Non-Hodgkin lymphoma, larynx and uterus. This is to investigate the possibility of an effect whereby eRATs are associated with increased

For peer review only - http://bmjopen.bmj.com/site/about/guidelines.xhtml

diagnostic activity beyond the eRAT cancers. We will also investigate differences in: the number of patients diagnosed with the six eRAT cancers combined, and the total number of cancers (excluding non-melanoma skin cancer) diagnosed, use of the 2-week wait referral system (the main pathway for urgent investigation of possible cancer in England) or equivalent for the six eRAT cancers combined, and across all cancers; the routes to diagnosis for each of the six eRAT cancers,(18) and for the six comparator non-eRAT cancers; the proportion of patients on a 2-week wait pathway receiving a diagnosis of cancer; whether a patient on a 2-week wait pathway has a diagnosis of cancer established (or refuted) within 28 days; 30-day and 1-year survival for those with cancer; the rate of cancer investigations, namely colonoscopies, sigmoidoscopies, upper gastro-intestinal endoscopies, chest x-rays, abdominal ultrasounds, and abdominal CT scans. We will also conduct parallel cost-effectiveness analyses, service delivery modelling and a process evaluation.

Methods and analysis

Design and setting

The study is a pragmatic cluster randomised-controlled trial in England, in primary care medical practices using one of the two (SystmOne or EMIS) electronic record keeping systems. The clusters are practices, a term which includes single practices, and small groups of practices agglomerated administratively to single entities. These will be randomised 1:1 to receive either the intervention (access to the suite of eRATs) or usual care. Appendix A shows pathways to a cancer diagnosis in the UK and illustrates how the intervention is expected to have an effect. It is unrealistic to offer eRATs to individual GPs, as there would be considerable contamination within any practice. Nevertheless, for a practice to be eligible to take part, we ask at least 50% of GPs in that practice to agree to use the eRATs. Although the intervention is at the practice level, some process and resource use measures and all main trial primary and secondary outcomes relate to individual patients.

Intervention

The eRATs

The eRATs have been developed by a specialist IT team, Informatica systems Ltd, in partnership with the cancer charity, Macmillan. The risk estimates in the eRATs are from the original research papers for each cancer site. (9, 19-24) Practices will access the software via a new cloud-based system called Skyline, specifically designed to facilitate efficient integration into GP clinical systems. CA marking of the Skyline version of eRATs was obtained in September 2021.

The eRATs have multiple functions. The first is the *'prompt'*. This collates relevant coded symptoms and blood tests in the patient's medical record from the previous 12 months, which are then assessed for the possibility of cancer, generating a risk score equivalent to the positive predictive value of the

For peer review only - http://bmjopen.bmj.com/site/about/guidelines.xhtml

For peer review only

cancer features for each cancer. A prompt (pop-up), displaying the risk score(s), appears on screen when a registered user opens a patient's medical records and indicates that patient has a risk of 2% or higher for at least one of the studied cancers. A second function is the *'symptom checker'*, allowing the clinician to add additional patient's symptoms to the eRAT checklist on screen; this process automatically recalculates the risk of any of the six cancers. On reviewing the risk score from the prompt and/or symptom checker, the clinician then decides the best course of management, which may be: (i) clinical review in primary care; (ii) ordering of test/investigations; or (iii) referral into secondary care. Embedded within all eRATS are links to authoritative guidance regarding the early diagnosis of cancer, NICE NG12,(25), Macmillan's abbreviated NICE guidance,(26) and Cancer Research UK guidance. (27) These sources of information are added to assist management of the patient, but the decision whether – or not – to investigate is for the clinician and patient. Some EMIS practices also have access to the QCancer risk tool, (28) albeit embedded in a dormant state within the practice IT and record system, and requiring manual activation prior to operation. All practices will be asked not to use it during the trial.

Justification of cancer sites

RATs are available for 18 adult cancers, each varying in their incidence, ease of diagnosis, amenability to treatment and proportion presenting as an emergency.

We elected to study cancer sites a) which were in the top 15 cancers by incidence; b) for which curative treatment is reasonably possible in symptomatic patients;(29) and c) with a significant percentage of patients presenting as an emergency.(30). Using these criteria, six cancer sites were selected, amounting to approximately half of all incident cancers. The selected six were: lung, colorectal, oesophago-gastric, ovary, kidney and bladder. The remaining nine cancers were considered as comparators to examine for any practice level effect of increased cancer diagnostic activity. Three of these nine cancers, brain, pancreas and leukaemia, were removed for clinical and practical reasons: no eRAT is available for brain or leukaemia; in both brain and pancreas, symptomatic diagnosis is considered to have a very small likelihood of improving survival,(29) and in leukaemia, a full blood count (easily available in primary care) will usually establish the diagnosis, making an eRAT unlikely to expedite the diagnosis.(31)

Training practices in using eRATs

Training in the use of the eRATs uses short, pre-recorded videos available online co-ordinated by a practice 'research champion'. These show GPs how to use the prompt and symptom checker functions.

For peer review only - http://bmjopen.bmj.com/site/about/guidelines.xhtml

Duration of intervention

Practice recruitment started in August 2019 and is expected to finish at the end of March 2022, including the installation of the eRATs software. The trial was paused for 6 months in March 2020 due to Covid-19. The formal start of the intervention window will be 01/04/2022 (although some practices may have delayed installation) and will close for all intervention practices on 31/3/2024.

Usual care

Patients presenting to the control practices will experience the GP's usual diagnostic approach. GPs in control practices will have no specific on-screen prompt, though they may have access to hard-copy (e.g. paper or mouse mat) versions of the RATs, or to other cancer tools such as those supporting structured follow-up of symptomatic patients not selected for initial investigation. For EMIS practices with QCancer dormant in the system, control practices are expected to leave it dormant. We will document control practice use of RATs, other decision support tools, and access to and use of eRATs via interim and exit questionnaires completed within the first 12 months of a practice commencing the intervention and at the end of the trial. In line with intervention practices, trial time will formally begin for control practices on 01/04/2022 and end on 31/03/2024.

Data collection window

Outcome data for all practices will be obtained for the 2-year period from 01/06/2022 to 29/05/2024. This data collection window is lagged behind the trial time window (01/04/2022 to 31/03/2024) in order to: a) provide some time for practices to become accustomed to how the intervention functions prior to data collection, and b) to have a 2-month window following the end of the intervention window in order to allow cancers to be diagnosed in patients seen towards the end of that window.

Sample size

There are around 130,000 new diagnoses of the six included cancers in the UK annually.(32) As each of our six cancer sites has different proportions diagnosed at an early stage, the sample size calculation is based on a relative improvement in staging, using an odds ratio of 0.8 for a cancer being diagnosed at Stage 3/4 in the intervention arm compared with the control arm. This difference is quite large and equates to an absolute reduction of 4.8% in the intervention arm as an incidence-weighted figure across the six cancers. A much smaller improvement would still be clinically valuable but would necessitate an impossibly large trial.

For the inflation factor we have used an intra-cluster correlation coefficient based on our previous work, of 0.05.(33) An average cluster size of 23 patients with a diagnosed cancer with recorded stage

For peer review only - http://bmjopen.bmj.com/site/about/guidelines.xhtml

during 2-year follow-up is expected, with a coefficient of variation for cluster size of 0.7, giving a design effect of 2.66. For an individually randomised trial with 90% power and an alpha threshold of 0.05, the sample size would be 2,049 patients per arm. Adding in the design effect, this becomes 5,497 patients, requiring 239 practices per arm, and 478 practices in total. Due to changes in practice structure (such as practice mergers, closures or divisions), we anticipate the loss of up to 10% of recruited practices over the course of the trial; to account for this we will recruit a target of 530 practices overall, expecting 12,190 patients to be diagnosed with cancer in total.

Practice recruitment

A total of 530 primary care practices across England will be recruited, supported by the NIHR Clinical Research Network (CRN) and strategic media releases to raise awareness of the trial. Practices that are proposing a split or a merger are not eligible for the trial, as the practices before or after the change may have been allocated to different arms in the trial. A method for identifying and managing unanticipated splits or mergers during the active phase of the trial is shown in Appendix B.

Patients are not being recruited into this trial - patient consent is not being sought for the use of the eRATs during the consultation. This is because ERATs are essentially an extension and enhancement of existing diagnostic tools already available to the GP to support their clinical decision making. Other randomised controlled trials of interventions in primary care have taken this approach,(34) including the feasibility trial of the oesophago-gastric eRAT.(14, 35, 36) To promote patient awareness of the practice's participation in the ERICA trial, including requesting practices to add it to their websites and any social media feed. A selection of patients will be recruited to the nested process evaluation and health economics studies (see below and Appendices B and D).

Randomisation

Practices will be randomised using a 1:1 ratio into one of two trial arms: usual diagnostic care (control) and usual diagnostic practice plus access to the suite of eRATs, as the intervention. Randomisation will be computer-generated and web-based, conducted by an independent member of staff at the Exeter Clinical Trials Unit (ExeCTU), overseen by the CTU statistician (not the trial statistician). To promote balance between the trial arms in practices' use of the 2-week wait system, and therefore propensity to refer to secondary care, we will minimise randomisation by age-sex standardised 2-week wait referral ratio (the best available proxy) in national tertiles. We will use simple randomisation to allocate the first 50 practices (~10% of the total target), and then apply minimisation by 2-week wait referral ratio tertile, taking into account the previous allocations to inform the minimisation algorithm. All allocations using the minimisation algorithm will retain a stochastic element, aimed at promoting allocation concealment.

For peer review only - http://bmjopen.bmj.com/site/about/guidelines.xhtml

The data analysis will be carried out by the trial statistician and health-economist, blinded to treatment allocation and all primary outcome data are objective assessments of clinical outcome. Staging (the primary outcome) will be performed by pathologists unaware of trial participation or allocation. However, given the nature of the intervention, it is not possible to blind GPs or the GP practice to treatment allocation.

Outcome measures

Primary outcome

Outcome measures will be captured at patient-level, using data routinely collected by the National Cancer Registration and Analysis Service (NCRAS). The primary outcome is whether a patient is diagnosed at stage 1 or 2 (early) or stage 3 or 4 (advanced). This division of staging is commonly used and is a targeted metric in the 2019 NHS Long Term Plan - for stage 1 and 2 cancers (for all staged cancers other than non-melanoma skin cancer) at diagnosis to comprise 75% of the total by 2028. The current UK overall incidence-weighted percentage of early stage at diagnosis was 55% in 2018, though for the six eRAT cancers, it is 35%.(37)

Secondary outcomes

A range of secondary outcomes will be examined:

1. The binary stage at diagnosis of a further six cancers without eRATs will be identified from NCRAS, and compared between intervention and control practices. This is to investigate the possibility of a ‘spill-over’ effect whereby eRATs are associated with increased diagnostic activity beyond the eRAT cancers.
2. The practice’s number of patients diagnosed with the six eRAT cancers combined, and the total number of cancer cases, from NCRAS.
3. The number of patients investigated or referred under the 2-week wait system for the six eRAT cancers combined, and in total, from Cancer Waiting Times data.
4. Route to diagnosis from the Routes to Diagnosis Dataset,(18) which uses Hospital Episode Statistics data. This will be categorised into four possible routes: emergency attendance, 2-week wait referral, GP referral, and “other”. We will collect this information for each of the six eRAT cancers, and for the six comparator non-eRAT cancers.
5. 2-week wait performance measures, from Cancer Waiting Times data, for the six eRAT cancers combined, and for all cancer referrals:

For peer review only - http://bmjopen.bmj.com/site/about/guidelines.xhtml

5.1 Whether a patient on a 2-week wait pathway received a diagnosis of cancer. When aggregated, for example at the practice-level, and expressed as the proportion of patients who received a cancer diagnosis, this is known as the conversion rate.

5.2 The duration between 2-week wait referral and diagnosis of cancer in days

5.3Whether patients referred on a 2-week wait referral and who received a cancer diagnosis were diagnosed within 28 days, the Faster Diagnosis Standard (introduced in 2022).

5.4 Detection rate – the proportion of a practice's cancers which are identified via the 2-week wait pathway.

6. Survival measures (from date of diagnosis): 30-day; 1-year (identified from NCRAS). 5-year survival will also be reported, but the main trial will report on 30 day and 1-year, with 5-year data being a subsidiary report. These outcomes will use all-cause mortality data from the Office for National Statistics.

7. Adverse events (using data from the Diagnostic Imaging Dataset): these are expected to be few, and largely related to complications from hospital investigation such as colonoscopy. There is no mechanism for adverse events to be collected using routine data. We will, however, estimate any change in the expected number of adverse events from imaging investigations (colonoscopies, sigmoidoscopies, upper gastro-intestinal endoscopies, chest x-rays, abdominal ultrasounds, and abdominal CT scans) through investigating any change in the rate of these investigations in intervention practices relative to control practices (see data analysis section). Potential adverse psychological consequences of being labelled with 'possible cancer' will be further explored in the process evaluation.

Data collection

All primary and secondary outcome measures are available from NCRAS, DID and publicly available practice level data, including Cancer Waiting Times data. We will be using depersonalised (pseudo-anonymised) data. The Public Health England Office for Data Release (ODR) guidelines indicated that no legal gateway (e.g., section 251 approval) will be necessary to obtain these data.

Data analysis

All analyses will follow CONSORT guidelines for cluster-randomised and pragmatic trials. The primary analysis, exploring the proportion of cancer patients with early stage at diagnosis, will use mixed-

For peer review only - http://bmjopen.bmj.com/site/about/guidelines.xhtml

effects logistic regression with a random intercept for practice to accommodate the hierarchical nature of the data (i.e. random allocation by practice, with participants nested within practice). This regression will include trial-arm at practice-level, and will adjust for patient-level covariates known to be associated with stage (age, sex, quintile of the income domain from the Index of Multiple Deprivation (IMD), and cancer site),(38) and the practice-level minimisation variable (national tertile of age-sex standardised two-week wait referral ratio). We will further adjust the model at the practice-level for list size, clinical IT system used, and Care Quality Commission (CQC) overall rating, should these variables be associated with stage in preliminary analyses (even if not unbalanced with respect to trial allocation). Trial arm and covariates will all be entered as fixed effects. The degree of change in the percentage of patients diagnosed at a late stage in intervention practices will be investigated by exploring the marginal distributions of trial arm on the probabilities predicted by these models.

For the secondary outcome of the stage at diagnosis of six cancers without eRATs, we will repeat the above model including data on the six non-eRAT cancers as well as the six eRAT cancers. This model will use all the variables described above, plus an indicator variable for whether the cancer site has an eRAT and an interaction term between this variable and trial arm. From this model, we will obtain odds ratios (with 95% CIs) for: (i) the "spill over" effect of having the intervention on cancer sites not included in the intervention, and (ii) for the relative effect of the intervention on stage for included cancer sites compared with those not included in the intervention.

Mixed-effects logistic regression models with a random intercept for practice will also be fitted for the other secondary binary outcomes; route to diagnosis, conversion rate, and timeliness. These models will include trial arm as a practice-level effect, and will adjust at the patient-level for age, sex, and quintile of the Index of Multiple Deprivation (IMD) income domain, and at the practice-level for the minimisation variable (national tertile of age-sex standardised two-week wait referral ratio). These analyses will also adjust at the patient-level for cancer site (routes to diagnosis analyses) or for referral type (2-week wait analyses) as appropriate. The models will be further adjusted as in the main outcome variable analysis.

Time-to-event secondary outcomes (length of waiting time, survival) will be analysed using mixed-effects parametric survival models with a random intercept for practice, and all other variables added as fixed effects. These models will include trial-arm as a practice-level effect, and will adjust for the same patient-level factors as described above (waiting times adjusted for referral pathway rather than cancer site as above), and the practice-level minimisation variable (national tertile of age-sex standardised 2-week wait referral ratio). The models will also use the same adjustment as the primary

For peer review only - http://bmjopen.bmj.com/site/about/guidelines.xhtml

outcome measure. An appropriate distribution to model the baseline hazard will be utilised, as determined by a comparison of the Akaike Information Criteria under different distributions.(39)

For rate outcomes (number of 2-week wait referrals, cancers, and imaging investigations), we will analyse the rates per 100,000 registered patients per year by age-sex strata using mixed-effects Poisson regression models including a random intercept for practice. These models will include trial-arm as a predictor and will adjust for the age and sex of the strata, and at the practice-level for the minimisation variable (2-week wait referral ratio) and deprivation (quintile of IMD overall score). The models will be further adjusted at the practice-level for list size, clinical IT system used, CQC overall rating, and for the age and sex case-mix of practices should these covariates be found to be associated with the outcome (even if not unbalanced with respect to allocation). Case-mix will be incorporated by including variables for counts of practice populations in different age-sex strata (5-year age groups by sex, excluding one age group-sex stratum that can be determined once all others are known).

All the above analyses will combine data for the six eRAT cancers for each model. For outcomes related to two-week wait referrals, data will be combined for all referral pathways relevant to the six eRAT cancers. To investigate whether the eRATs produce a "spill-over" effect, whereby diagnostic activity is increased for other cancers, we will repeat all analyses using data for the six non-eRAT cancers combined for each model. Investigation of a spill-over effect for 2-week wait referral outcomes will use data for all referral pathways combined.

Additional sensitivity analyses will be conducted for the primary outcome in order to explore moderation arising from practice-level characteristics, using interaction terms. Although the trial has not been powered to detect low to moderate subgroup differences, such as differences in a single cancer site, large interaction effects that differ with respect to the direction of effect across subgroups are of interest. The potential impact of missing staging data on the primary outcome will also be explored through use of multiple imputation methods making use of auxiliary variables such as survival time, morphology and grade to improve the Missing At Random (MAR) assumption in line with previous work).(40)

Data management

Cancer registry data (NCRAS) will be managed and prepared by the registry themselves and securely, electronically transferred to the study team. There will be no patient identifiable data within these datasets. Data from NCRAS will be stored on the Secure Data Resource Hub at the University of Exeter (which meets requirements for secure storage of sensitive data) and linked to existing practice

For peer review only - http://bmjopen.bmj.com/site/about/guidelines.xhtml

data held within ExeCTU's REDCap database. The data will be stored and retained in accordance with registry policies.

The nested studies rely on identifying patients from in-practice usage reports. These reports contain depersonalised (pseudo-anonymised) data. The practice will send a copy to the trial team with the original practice ID number removed. The local at practice reports will be securely and electronically transferred to a secure Exeter CTU computer.

In the recruitment of patients (and NHS staff) for interviews, questionnaires, or permission for access to medical notes, participant details will be passed securely between NHS services and the research team. All participants agreeing to interview, to complete a questionnaire, and/or medical notes review, and all GPs agreeing to interview will be allocated a unique study ID, and the information linking their ID to their personal details will be kept securely at the University of Exeter. All other participant-related paper records will be anonymised and stored separately from the personal information. The electronic database for the trial will be stored on the secure servers of the University of Exeter with password-controlled access provided for the research team by ExeCTU. Single data entry with extensive in-built validity checks will be used to reduce the risk of transcription errors.

Audio recordings will be digitised, encrypted and stored on the University's secure server. Audio recordings will be retained until after anonymised transcripts have been finalised and analysed. At this stage they will be securely and permanently deleted. Access to personal data will be restricted to the research team. Names and participant details will not be passed to any third parties and no named individuals will be included in the outputs. All participants (patients, NHS staff) will be asked for their consent for the study team to retain interview transcripts for the purposes of future research by those involved directly in the study team or to be used for educational purposes.

Informatica Systems Ltd has developed a separate agreement ('Data processing deed') for intervention practices which will be used between the GP practices and Informatica Systems Ltd. The deed was necessary because the development of Skyline has impacted on the processing arrangements for the eRATs software that is used. The ERICA research study will still use the Organisation Information Document which outlines the research team's data processing requirements, to be signed between the practice and Sponsor.

All study data will be kept for 10 years (unless data registry policy requires otherwise) under secure conditions on University of Exeter secure servers. Data will also be subject to standard secure storage and usage policies.

For peer review only - http://bmjopen.bmj.com/site/about/guidelines.xhtml

For peer review only

Trial monitoring and management

Trial Sponsor and Funders

The University of Exeter is the trial sponsor. The trial funders are providing finance to run the trial. None of the funders or sponsor will be involved in the design or day-to-day conduct of the trial, analysis of data, or interpretation of findings.

Trial Steering Committee (with Data Monitoring Committee responsibilities)

The responsibilities of the Trial Steering Committee (TSC) will be to review the main study protocol and any amendments, monitor and supervise the trial towards its interim and overall objectives, review relevant information from other sources, and to help resolve problems brought by the Trial Management group (TMG). The TSC will therefore provide overall independent supervision for ERICA on behalf of the funders and the Sponsor. Meetings will be held at regular intervals determined by need and not less than twice a year. Routine business will be conducted by telephone, videoconference, and email. The TSC will also operate as a Data Monitoring Committee with responsibility to monitor the overall conduct of the trial. There will be a time lag between practices 'entering the trial' and data availability from cancer registries. The time lag will be such that data will only be available once practices have completed data collection. Therefore, interim analyses to assess whether the trial was effective, and to support a decision whether to stop the trial early, would be unnecessary as data collection (and practice participation) would have already ceased.

Trial Management Group

A TMG has been established and includes those responsible for the day-to-day management of the trial and those supporting the delivery of the trial and associated stakeholders, including representatives of the Local Clinical Research Networks (LCRN) and Macmillan. The group will monitor all aspects of the conduct and progress of the trial, ensure that the protocol is adhered to and take appropriate action to safeguard participants and the quality of the trial itself. The group will meet regularly (monthly in the first instance, until recruitment has completed) in person and/or by phone or over the internet (via MS Teams).

Core Study Team

The core study team (Chief investigator, Trial Manager (TM)) will meet weekly during the study. Day-to-day running of the trial will be the responsibility of the TM. The TM will have access to the ExeCTU suite of standard operating procedures (SOPs) and will ensure that the trial is run in compliance with

For peer review only - http://bmjopen.bmj.com/site/about/guidelines.xhtml

all relevant SOPs (e.g., assessment, processes and reporting, data management, study staff health and safety).

Nested Studies

Health Economics

We will estimate the cost and cost-effectiveness of the eRATs versus usual diagnostic practice using the primary perspective of the NHS and Personal Social Services (i.e. third-party payer). We will estimate the cost-effectiveness of the intervention based upon the primary outcome and secondary survival outcomes (30-day and 1-year; 5-year survival will be a subsidiary report) for the six cancer sites with eRATs and report the results using the latest guidelines.(41) colorectal, lung and ovarian cancers we will use decision analytic models to combine data from the within-trial analysis of ERICA intervention on costs and benefits, with longer estimates derived from the evidence synthesis of the costs and benefits of stage of diagnosis and disease progression to estimate the cost per Quality Adjusted Life Year (QALY) over the longer term.(42) For fuller details see Appendix C.

Service Delivery Modelling

This will investigate the key factors central to the (re) organisation of NHS diagnostic services for cancer referrals. We will use a range of methods, both quantitative and qualitative, to analyse service delivery alternatives. Specifically, we will aim to use modelling approaches to explore the likely implications of different scenarios across dimensions of performance, outcomes and costs. Fuller details are in Appendix D.

Process Evaluation

The process evaluation work aims to identify and investigate the contextual factors that impact upon the effectiveness of the eRATs with a particular focus on intervention fidelity and GP engagement. The impact of the eRATs on the patients' experience of their GP consultation and their experiences of subsequent care will also be explored. Fuller details are in Appendix E.

GP Workload

This nested study aims to explore, in terms of consultation time, the impact of GPs using eRATs on GP workload and patient 'flow' through consulting sessions. It will also explore workload in the week following the index consultation in which an eRAT was activated. Fuller details are in Appendix F.

For peer review only - http://bmjopen.bmj.com/site/about/guidelines.xhtml

Patient and Public Involvement and Engagement

Our Patient and Public Involvement and Engagement (PPIE) group, including cancer survivors, have been consulted widely during the development of this study. The PPIE group have reviewed and commented on the protocol and supported the development of all patient-facing materials including information sheets and study lay summaries. One experienced PPIE representative sits on the TMG and another is on the TSC. A total of seven people have joined our PPIE group for this study and will contribute by reviewing study materials and documentation, commenting upon and proof reading reports and contributing to dissemination activities. This group will be supported in their work by the South West Peninsula Applied Research Collaboration (PenARC) PPIE team, for example by attending workshops on critical appraisal skills. All PPIE representatives will be recompensed for their time given to the study.

Ethics and Dissemination

A trial publication policy will be developed which outlines the plan for dissemination and will be in accordance with the International Committee of Medical Journal Editors. The results of the trial will be reported first to study collaborators and to the funder. The main report will be drafted by the TMG and circulated to all collaborators and the TSC for comment.

Access to the final trial datasets will be made publicly available unless contractual agreements between data providers limit such access.

Ethical review

The trial has received favourable Ethical review from London City & East Research Ethics committee, reference number 19/LO/0615, with eight amendments between then and 2022, relating to three main areas: the delays caused by the COVID-19 pandemic, with its recruitment moratorium; an alteration in the mechanism by which the eRATs software were delivered; and the inclusion of a nested study focussing on the impact of eRATs on GP workload. Current protocol version – V 6.0, 8th August, 2022.

Author contributions: WH conceived of the trial. Substantial contributions to the design of the methods and research processes were made by WH, JC, LM, SD, GA, MP, AS, AML, FW, EF, ES, MS, AM and RC. The protocol was written by RC, LM, SD, GA, AS, EF, and MP under the overall editorial control of WH. All authors critically reviewed the protocol and provided approval of the final version.

Funding statement: This research is funded by a philanthropic donation by The Dennis and Mireille Gillings Foundation, Cancer Research UK (C8640/A23385), plus support from Macmillan in provision

For peer review only - http://bmjopen.bmj.com/site/about/guidelines.xhtml

of staff time, and the University of Exeter. The trial is registered with ISRCTN: (trial no: ISRCTN22560297) and on the CRUK trial registry (CRUK database no: 16163).

Acknowledgements: We would like to thank the NIHR Clinical Research Network for their support with recruitment, Macmillan for their contributions to the early eRAT work and ongoing support with practice recruitment and pilot testing. SD's time is partially supported by the National Institute of Health Research Applied Research Collaboration (ARC) South-West Peninsula.

Disclaimer: The views expressed are those of the authors and not necessarily those of the NHS, the NIHR or Department of Health.

Competing interests statement: WH has intellectual property rights to the original RATs, though has never sought to commercialize these. All other authors: no competing interests to declare.

For peer review only

References

1. Richards MA. The size of the prize for earlier diagnosis of cancer in England. Br J Cancer. 2009;101.
2. Richards M, Thorlby R, Fisher R, Turton C. Unfinished business: An assessment of the national approach to improving cancer services in England 1995–2015. London; 2018.
3. NHS England. (2019) The NHS Long Term Plan [Available from: https://www.longtermplan.nhs.uk/.
4. de Koning HJ, van der Aalst CM, de Jong PA, Scholten ET, Nackaerts K, Heuvelmans MA, et al. Reduced Lung-Cancer Mortality with Volume CT Screening in a Randomized Trial. New England Journal of Medicine. 2020;382(6):503-13.
5. Richards M. THE INDEPENDENT REVIEW OF ADULT SCREENING PROGRAMMES. NHS England; 2019. Contract No.: 01089.
6. Landy R, Pesola F, Castanon A, Sasieni P. Impact of cervical screening on cervical cancer mortality: estimation using stage-specific results from a nested case-control study. Br J Cancer. 2016;115(9):1140-6.
7. Abel GA, Mendonca SC, McPhail S, Zhou Y, Elliss-Brookes L, Lyratzopoulos G. Emergency diagnosis of cancer and previous general practice consultations: insights from linked patient survey data. British Journal of General Practice. 2017.
8. Hamilton W, Walter FM, Rubin G, Neal RD. Improving early diagnosis of symptomatic cancer. Nature Reviews Clinical Oncology. 2016;13:740.
9. Hamilton W, Peters TJ, Round A, Sharp D. What are the clinical features of lung cancer before the diagnosis is made? A population based case-control study. Thorax. 2005;60(12):1059-65.
10. NICE. Suspected cancer: recognition and referral [NG12] 2015 [Available from: https://www.nice.org.uk/guidance/ng12.
11. Hamilton W, Green T, Martins T, Elliott K, Rubin G, Macleod U. Evaluation of risk assessment tools for suspected cancer in general practice: a cohort study. British Journal of General Practice. 2013;63(606):e30-e6.
12. Price S, Spencer A, Medina-Lara A, Hamilton W. Availability and use of cancer decision-support tools: a cross-sectional survey of UK primary care. British Journal of General Practice. 2019;69(684):e437.
13. Medina-Lara A, Grigore B, Lewis R, Peters J, Price S, Landa P, et al. Cancer diagnostic tools to aid decision-making in primary care: mixed-methods systematic reviews and cost-effectiveness analysis. Health technology assessment (Winchester, England). 2020;24:66.
14. Rubin G, Walter FM, Emery J, Hamilton W, Hoare Z, Howse J, et al. Electronic clinical decision support tool for assessing stomach symptoms in primary care (ECASS): a feasibility study. BMJ Open. 2021;11(3):e041795.
15. Kostopoulou O, Arora K, Pálfi B. Using cancer risk algorithms to improve risk estimates and referral decisions. Communications Medicine. 2022;2(1):2.
16. Hamilton W. Diagnosing symptomatic cancer in the NHS. BMJ : British Medical Journal. 2015;351:h5311.
17. Møller H, Gildea C, Meechan D, Rubin G, Round T, Vedsted P. Use of the English urgent referral pathway for suspected cancer and mortality in patients with cancer: cohort study. BMJ. 2015;351.
18. Elliss-Brookes L, McPhail S, Ives A, Greenslade M, Shelton J, Hiom S. Routes to diagnosis for cancer - determining the patient journey using multiple routine data sets. Br J Cancer. 2012;107.
19. Hamilton W. The CAPER studies: five case-control studies aimed at identifying and quantifying the risk of cancer in symptomatic primary care patients. Br J Cancer. 2009;101 Suppl 2:S80-6.
20. Hamilton W, Peters TJ, Bankhead C, Sharp D. Risk of ovarian cancer in women with symptoms in primary care: population based case-control study. BMJ. 2009;339:b2998.

For peer review only - http://bmjopen.bmj.com/site/about/guidelines.xhtml

21. Hamilton W, Round A, Sharp D, Peters TJ. Clinical features of colorectal cancer before diagnosis: a population-based case-control study. Br J Cancer. 2005;93(4):399-405.
22. Shephard E, Neal R, Rose P, Walter F, Hamilton WT. Clinical features of kidney cancer in primary care: a case-control study using primary care records. The British journal of general practice : the journal of the Royal College of General Practitioners. 2013;63(609):e250-5.
23. Shephard EA, Stapley S, Neal RD, Rose P, Walter FM, Hamilton WT. Clinical features of bladder cancer in primary care. The British journal of general practice : the journal of the Royal College of General Practitioners. 2012;62(602):e598-604.
24. Stapley S, Peters TJ, Neal RD, Rose PW, Walter FM, Hamilton W. The risk of oesophago-gastric cancer in symptomatic patients in primary care: a large case-control study using electronic records. Br J Cancer. 2013;108(1):25-31.
25. NICE. Suspected cancer: recognition and referral [NG12]2015. Available from: http://www.nice.org.uk/guidance/NG12.
26. Macmillan. (2015) Rapid referral guidelines [available at: https://www.macmillan.org.uk/_images/rapid-referral-toolkit-desktop_tcm9-291864.pdf].
27. Cancer Research UK. (2017) Cancer Stats 2017 [Available from: http://www.cancerresearchuk.org/health-professional/cancer-statistics].
28. Collins GS, Altman DG. Identifying patients with undetected gastro-oesophageal cancer in primary care: External validation of QCancer(R) (Gastro-Oesophageal). European journal of cancer (Oxford, England : 1990). 2013;49(5):1040-8.
29. Hamilton W, Stapley S, Campbell C, Lyratzopoulos G, Rubin G, Neal R. For which cancers might patients benefit most from expedited symptomatic diagnosis? Construction of a ranking order by a modified Delphi technique. BMC Cancer. 2015;15:820.
30. McPhail S, Johnson S, Greenberg D, Peake M, Rous B. Stage at diagnosis and early mortality from cancer in England. Br J Cancer. 2015;112(s1):S108-S15.
31. Shephard EA, Neal RD, Rose PW, Walter FM, Hamilton W. Symptoms of adult chronic and acute leukaemia before diagnosis: large primary care case-control studies using electronic records. British Journal of General Practice. 2016;66(644):e182-e8.
32. UK CR. Statistics by Cancer Type 2022 [Available from: https://www.cancerresearchuk.org/health-professional/cancer-statistics/statistics-by-cancer-type.
33. Barclay ME, Lyratzopoulos G, Greenberg DC, Abel GA. Missing data and chance variation in public reporting of cancer stage at diagnosis: Cross-sectional analysis of population-based data in England. Cancer Epidemiology. 2018;52:28-42.
34. Campbell JL, Fletcher E, Britten N, Green C, Holt TA, Lattimer V, et al. Telephone triage for management of same-day consultation requests in general practice (the ESTEEM trial): a cluster-randomised controlled trial and cost-consequence analysis. The Lancet. 2014;384(9957):1859-68.
35. Downs M, Turner S, Bryans M, Wilcock J, Keady J, Levin E, et al. Effectiveness of educational interventions in improving detection and management of dementia in primary care: cluster randomised controlled study. BMJ. 2006;332(7543):692.
36. Flottorp S, Oxman AD, Håvelsrud K, Treweek S, Herrin J. Cluster randomised controlled trial of tailored interventions to improve the management of urinary tract infections in women and sore throat. Bmj. 2002;325(7360):367.
37. National Disease Registration Service:. Case-mix adjusted percentage of cancers diagnosed at stages 1 and 2 in England, by Clinical Commissioning Group2020 25 Jan 2022. Available from: https://www.gov.uk/government/statistics/case-mix-adjusted-percentage-cancers-diagnosed-at-stages-1-and-2-by-ccg-in-england/national-disease-registration-service-case-mix-adjusted-percentage-of-cancers-diagnosed-at-stages-1-and-2-in-england-by-clinical-commissioning-group.
38. Lyratzopoulos G, Abel GA, Brown CH, Rous BA, Vernon SA, Roland M, et al. Socio-demographic inequalities in stage of cancer diagnosis: evidence from patients with female breast, lung, colon, rectal, prostate, renal, bladder, melanoma, ovarian and endometrial cancer. Annals of Oncology. 2012;24(3):843-50.
39. Bozdogan H. Model selection and Akaike's Information Criterion (AIC): The general theory and its analytical extensions. Psychometrika. 1987;52(3):345-70.

For peer review only - http://bmjopen.bmj.com/site/about/guidelines.xhtml

40. Lyratzopoulos G, Abel GA, Brown CH, Rous BA, Vernon SA, Roland M, et al. Socio-demographic inequalities in stage of cancer diagnosis: evidence from patients with female breast, lung, colon, rectal, prostate, renal, bladder, melanoma, ovarian and endometrial cancer. Ann Oncol. 2013;24(3):843-50.
41. Husereau D, Drummond M, Augustovski F, de Bekker-Grob E, Briggs AH, Carswell C, et al. Consolidated Health Economic Evaluation Reporting Standards 2022 (CHEERS 2022) statement: updated reporting guidance for health economic evaluations. BMJ. 2022;376:e067975.
42. Weinstein MC, O'Brien B, Hornberger J, Jackson J, Johannesson M, McCabe C, et al. Principles of Good Practice for Decision Analytic Modeling in Health-Care Evaluation: Report of the ISPOR Task Force on Good Research Practices—Modeling Studies. Value in Health. 2003;6(1):9-17.

For peer review only

For peer review only - http://bmjopen.bmj.com/site/about/guidelines.xhtml

A pragmatic cluster randomised controlled trial assessing the clinical effectiveness and cost-effectiveness of electronic risk-assessment for cancer for patients in general practice (ERICA): Appendices

For peer review only

Appendix A. A simplified schema illustrating the pathways to a cancer diagnosis in the UK. The size of the arrow reflects the approximate proportion of cancers taking each route. The yellow central arrow represents where eRATs are expected to have an effect.

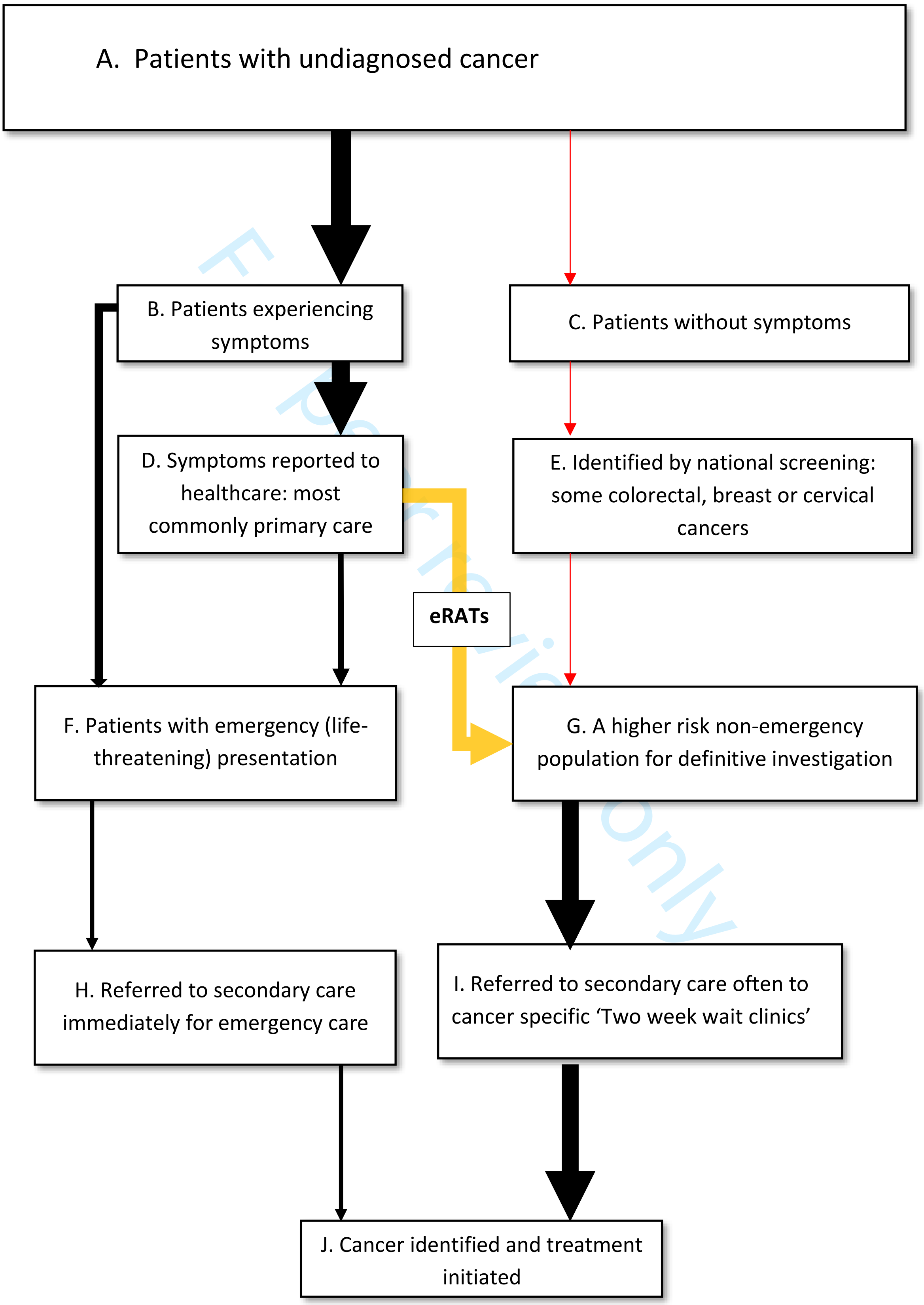

Appendix B. Managing practice splits and mergers in analyses

Although we will exclude practices that report imminent restructuring during recruitment, there may be unforeseen mergers or splits of practices. Where mergers and splits are concerned, this could mean, for example, that some of our practices who were in the control arm may merge with an intervention practice. Similarly, a non-trial practice may become part of a trial practice (intervention or control). Changes in practice size have implications on the denominator – the number of patients that each practice is likely to be contributing to our sample – and is a particular issue for three of our secondary outcome measures based on rates (cancer diagnosis rate, two-week wait referral rate, and adverse event rate). Importantly, however, this issue is not a problem for our primary outcome of staging.

We define a split and mergers as follows: Split – Where a population of patients registered to a single practice with a single practice code become registered with two or more individual practices with different practice codes. The practice codes of the new practices may be new codes (i.e. did not exist prior to the split) or one may inherit the original practice code (although this is not a requirement). The change in registration of patients must occur to a substantial number of patients and not at their request. Merger – Where a population of patients registered to one or more practices with different practice codes become registered at a single individual practice with a single practice code. The practice code of the new practice may be a new code (i.e., did not exist prior to the split) or it may inherit one of the original practice codes. A federation is not a "merger" in these terms.

Excluding practices who restructure during the trial may unnecessarily reduce our power. Therefore, we will try and accommodate changes in status. The Table outlines our approach. The assumption is that the change takes place at time T. Any practice which splits goes from X to Y and Z, and mergers are Z plus Y becoming X. Intervention practices are I, and comparison practices C.

Practice size fluctuations will be monitored in real time. Practice size data are freely and publicly available from NHS Digital and are updated monthly. Each month during the data collection, the trial statistician will download the practice size data and inspect size for all the practices in the trial (the statistician will remain blinded to outcome allocation). If the practice size differs by more than 10% the statistician will alert the trial manager, who will contact the research champion in the relevant practice to explore the reasons for this practice size change. Reasons (e.g., mergers, splits) will be recorded.

For peer review only

Table: managing changes in practice size – mergers and splits

<table>
<tr><th>Split or merger</th><th>X pre change</th><th>Y pre change</th><th>Z pre change</th><th>X post change</th><th>Y post</th><th>Z post</th></tr>
<tr><td rowspan="2">Split</td><td>I</td><td></td><td></td><td></td><td>I</td><td>I</td></tr>
<tr><td>C</td><td></td><td></td><td></td><td>C</td><td>C</td></tr>
<tr><td colspan="7">We will allow the daughter practices to withdraw from the trial if they desire, which would mean we lose Y or Z (or both). If daughter practices decide to withdraw, we will include data up to time T plus 2 months to allow for average diagnostic time to cancer.</td></tr>
<tr><td rowspan="5">Merger</td><td></td><td>I</td><td>I</td><td>I</td><td></td><td></td></tr>
<tr><td></td><td>I</td><td>C</td><td>I</td><td colspan="2" rowspan="2">There is likely to be wash over under these conditions, so the merged practice will be considered as I</td></tr>
<tr><td></td><td>I</td><td>Non-trial</td><td>I</td></tr>
<tr><td></td><td>C</td><td>Non-trial</td><td>C</td><td></td><td></td></tr>
<tr><td></td><td>C</td><td>C</td><td>C</td><td colspan="2"></td></tr>
</table>

We will manage changes in practice size at the data analysis stage of the trial. Where changes in list size of more than 10% within a month are seen, data for that practice will not be included in the analysis of rate outcomes from one month prior to the change. There are two exceptions to this; 1) splits where all the daughter practices remain in the trial and we continue to treat them as a single practice for rate analyses, 2) mergers where merged practices are in the same arm of the trial, and we will analyse them as a single practice from the start for rate analyses.

Appendix C

Health Economics

Intervention costings. The resources used in developing the training materials and videos (preparation and IT support) will be collected from the trial manager; nationally applicable unit costs will be applied. Estimates of the extent to which these videos are watched by practice staff will be based on information available from the website platform hosting the videos. Information on the resources use to install the eRATs onto the EMIS and SystmOne practice IT systems will be estimated in consultation with practice champions. These estimates will additionally aim to estimate: 1) the cost of installation in the trial and 2) the anticipated cost of future installation should eRATs be implemented nationally.

For peer review only

Health related quality of life and resource use. The Health Economics analysis will draw on the estimated number of imaging investigations (colonoscopies, sigmoidoscopies, upper gastro-intestinal endoscopies, chest x-rays, abdominal ultrasounds, and abdominal CT scans) using data from the Diagnostic Imaging Dataset available in the main trial as well as estimates of GP workload from the process evaluation. Practices will be offered remuneration of nearly £200 for the additional work.

To investigate whether the eRATs intervention was associated with a change in health-related quality of life using the EQ5D-5L and to provide more detailed information on primary care services and tests used, we will sample patients in the intervention arm who had a consultation where an eRAT alert occurred, and patients in the control arm who had a consultation where an eRAT alert would have occurred. We will strategically target practices in both trial arms who have either high, medium, or low two-week wait referral rates, matching the minimisation criteria in the main trial. It is anticipated that 15-20 patients per practice over a 2-week period will have a consultation with an eRAT alert. All patients who have an eRAT alert will be invited to complete a baseline questionnaire and a 3 month follow-up Health Economics questionnaire, as will equivalent patients in the control arm. We anticipate that 40% of patients will accept, and of these there will be 20% who do not respond. With a conservative estimate of a cluster size of five patients responding to the questionnaire, plususing an minimum clinically important difference of 0.1 for the EQ5D-5L (2) and a standard deviation of 0.23(3), with an inter-cluster correlation coefficient of 0.03 (4), and an estimated coefficient of variation of cluster size of 0.7, the sample size required to detect a between group difference with 90% power and alpha of 0.05 was 28 clusters (140 participants) per arm. Participants who agree to take part will receive the questionnaire as a hard copy, through the post, or electronically via email, depending on the participant's preference. Nationally applicable unit costs will be used for all community health and social care contacts (5) and secondary care services, tests and investigations will be costed using the National Schedule of Reference Costs 2016-2017. (6)

For peer review only

Decision Analytic Model

The modelling aims to predict the expected impact of a change in stage of diagnosis, and any resulting change in the distribution of cancer stage at diagnosis (intervention vs. control) over time, building on the published literature in this area.(7-10) The decision analytic models will not need to separately model the diagnostic phase, and we will take the trial's primary outcomes, stage at diagnosis (Stage 1-4 separately and not collated into Stage 1-2 and Stage 3-4), to model the longer term effects on survival, QALYs and secondary care costs.

Scenario analysis will be used to examine the impact on the results of multiple parameters changing simultaneously (based on *a priori* judgement about the combination of parameters to include).(11) Probabilistic sensitivity analysis will be used to explore the proportion of results that are considered cost-effective in relation to a given cost-effectiveness threshold and these results will be illustrated graphically using a cost-effectiveness acceptability curve.(12)

The study will follow the CHEERs guidelines for reporting cost-effectiveness studies and models,(13) and will discount both costs and outcomes at 3.5% as recommended by the National Institute of Health and Care Excellence.(14) Sensitivity analyses will examine alternative assumptions about the missing data mechanisms.(15)

Service Evaluation

We will draw upon published systematic reviews of Quality of Life measures, that are based on public preferences and measured in patients (as required by NICE guidelines (16) and that have been used for economic evaluation modelling studies.(17)

Appendix D

Service Delivery Modelling

Background and rationale

Cancer diagnosis has become one of the principal areas of focus and concern for the NHS in England.(18) For some time, NHS performance in both early diagnosis, delays in referral, and associated survival rates has been poor relative to our national aspirations and when compared with other first world countries. This has worsened during the COVID pandemic. In this context, many of the issues of concern are centred on key aspects of service delivery. How the NHS organises its services is often pivotal in determining the cost, feasibility, and effectiveness. For instance, factors such as workforce availability, prioritisation, service location, scale, and resources are fundamental to the performance of the NHS in delivering effective cancer services.

This component of the ERICA programme will investigate the key factors central to the organisation of NHS diagnostic services for cancer referrals. We will use a range of methods, both quantitative and qualitative, to analyse service delivery alternatives. Specifically, we will aim to build an economic model to assess the likely implications of different scenarios. Implementation of the eRAT diagnostic tool at primary care level is likely to impact directly on the follow-on pathway for cancer diagnosis (for example in terms of the volume and case mix of referred patients for diagnosis). Our model will

For peer review only

therefore provide an assessment of the likely effect of this impact in terms of costs and performance, and highlight any changes in organisation that might be implied by the introduction of the eRAT tool.

This research will run in parallel with the substantive work conducted for the controlled trial of eRAT implementation within ERICA. It will also liaise closely with the detailed and standard analysis of cost-effectiveness for disease progression (which is inherently abstracted from the service delivery aspects of care) in order to provide an added dimension to the cost-effectiveness outputs from the ERICA study as a whole.

Objectives

To build and populate a model of the cancer diagnostic pathway for England, in order to provide an assessment of the costs and effectiveness of different scenarios for service delivery. In particular, we will investigate the potential aspects relative to implementation of eRATs based on the study data collected from the ERICA trial. In addition, qualitative research with NHS staff in secondary care will be used to assess key areas central to successful implementation and sustainability.

Methods

A wide range of methods will be essential to fulfil the objectives of the work outlined here. Early work will include a literature search and survey of current systems for diagnostics in cancer. We will therefore conduct a systematic review of the related literature in the field and carry out a survey of current service delivery organisation across a range of settings. This work will aim to identify the key factors bearing on the organisation of services such a regional variation, metropolitan versus rural context, and population case mix differences.

Phase two work will aim to build a model in order to capture the key elements of service delivery for diagnostic services for cancer. This will explore a range of modelling approaches and test which is most suited to specific needs. For example, discrete event simulation, Systems Dynamics, geographic analysis, and Markov modelling will all be tested in terms of their relevance and appropriateness to specific requirements. In this context it is highly likely that different modelling tools will be relevant to the diverse needs of the study, so no single approach will be dominant.

Phase three will focus on the service delivery implications for the introduction of the eRAT diagnostic tool in primary care looking particularly at the potential knock-on effects in other areas of service.

In addition to our modelling work, we will use qualitative methods, such as problem structuring methods, soft systems mapping, to provide an assessment of some key elements of implementation.

For peer review only

Data

A wide range of data will be used to complete this component of the work. We will aim to integrate sources from across routinely collected datasets such as those listed below to construct our models: NHS activity data, Waiting time data, Reference cost data, Diagnostic Imaging Data (DIDs), Hospital Episode Statistics (HES), Workforce reference data, GP and hospital referral data, QOF data, Population data (e.g. ONS). In addition, we will aim to incorporate the primary data derived from the main ERICA study in order to model and assess the pathway impact from the use of eRATs. We will also use the outputs from the standard economic analysis as an input for the cost effectiveness of the service delivery modelling. Output from the qualitative research will also provide important data for informing the outputs of this work, for example in feeding into the recommendations and conclusions of the study.

For peer review only

Appendix E

Process Evaluation

Scope of process evaluation

The process evaluation work aims to identify and investigate the contextual factors that impact upon the effectiveness of the eRATs with a particular focus on intervention fidelity and GP engagement. The impact of the eRATs on the patients' experience of their GP consultation and their experiences of subsequent care will also be explored. It is underpinned by the COM-B framework for understanding behaviour change (19). This framework will outline the interactive nature of how the GP's capability (IT skill for using the eRATs), opportunity (eRAT prompts), and motivation (to do the training and use all the eRAT features) might influence their behaviour – i.e. ongoing use of the eRATs, symptom checker, coding of symptoms and changes to referral letters. We will use a mixed-methods approach to explore how the intervention was delivered (including fidelity and dose - if the eRATs were being used as intended and their degree of use across intervention practices and over time) and GP engagement with and acceptability of using the eRATs (GP's experiences of the eRATs).(20) For delivery, we will be particularly interested in fidelity of function. (21) GPs will be given clear training videos on how to use the eRATs and we will explore the extent to which GPs engaged with training as well as how they subsequently engaged with the software, and the GP's experiences of how it impacted on the GP-patient relationship in order to evaluate how they responded to the intervention.

Methods

Intervention fidelity and GP engagement (intervention arm only): Prior to the start of the intervention GPs require a minimum level of training in how to use the eRATs. Although the software is designed to be intuitive, a clinical system specific walkthrough for the two main functions of the eRATs (prompt and symptom checker) and FAQs will be available via separate videos. The research champion will be

given access to the videos and can disseminate the video content to all GPs in the practice (by showing the videos during a practice meeting, providing a demonstration themselves, or passing on the weblink). Once practices have started the data collection phase, we will invite up to 10 research champions to interview to discuss in depth their experiences of the set-up and training procedures and to explore whether their GPs have the capability, opportunity and motivation to use the eRATs. We will purposively sample research champions based on whether they are from a practice with a high, middle or low two-week wait referral rate, which software system their practice uses, their gender, and their level of experience in practice (10+ years vs. less than 10 years in practice).

Detailed eRAT usage can be captured for all IT systems. Usage will be captured in two ways – i) via a central log and ii) via local 'at practice' reports. For i), usage logs will be routinely and automatically sent from the practice to the Informatica 'digital warehouse' and will contain anonymised, practice-level data for each eRAT including reports of: how many times the prompt was shown, how many times the symptom checker was used, the number of times the symptoms were changed during use of the symptom checker, the length of time the symptom checker was open for, and whether clinical guidance was accessed from the eRAT. These centrally reported logs will be available on a monthly basis throughout the course of the trial and will be securely sent from Informatica to the research team who will add the data to the trial database.

For ii), usage will be examined via reports run locally at each practice. These reports include individual patient level data outlining which eRAT was triggered, the patient's risk score on the eRAT, when the symptom checker was opened and closed, patient's age and sex, and a list of possible eRAT symptoms and whether they were changed. These reports contain depersonalised (pseudo-anonymised) data. As it is possible to potentially identify the patient via the practice ID number we will ask practices to make a copy of the report, add in a new patient study ID variable (e.g., p1, p2, p3, etc) and save it to the practice computer. We will then ask them to send a copy to the trial tram with the original practice ID number removed. They will also send the file with a predetermined practice ID number. These measures should ensure the data is anonymised. The local at practice reports will be securely and electronically transferred to a secure Exeter CTU computer.

Intervention fidelity (Intervention and control). We will ask all research champions in the intervention practice to complete a short questionnaire (online via a secure, University approved provider) detailing their experience of installing software, using the eRATs, and whether alternative risk tools have also been used. We will ask research champions at control practices their experiences of being in the trial and whether they have started using any cancer risk tools. The questionnaires will be

For peer review only

completed at two time points – i) within 12 months of the start of the intervention; ii) at the end of the data collection period.

For identifying GPs to interview, we will use maximum variation purposive sampling (sampling on practice two-week wait referral rate (high vs. medium vs. low); software system used, gender, length of time in practice (10+years vs. < 10 years), and working status (part time vs full time)) and expect to interview up to 18 GPs from intervention practices to ask them about their experience of the eRATs including the training provided, any impacts on the consultation and their clinical decision making, as well as any changes in symptom coding behaviour. We will invite GPs to interview after the intervention has been running for at least 3 months. Written information will be provided about the interview study and written consent will be taken prior to the interview and will be verbally confirmed before the interview commences. Interviews will be audio-recorded and carried out by telephone, face-to-face (only if it is safe to do so), or over the internet (e.g., Zoom or MS Teams) depending on the GP's preference, by members of the research team using a pre-defined topic guide that focuses on their training and capability to use eRATs, their opportunity to use the eRATS over the study period and their motivation to continue using the system. If a face-to-face interview is chosen (and safe to perform), interviews will take place in a private room at the practice. The researcher will comply with the lone worker policy, ensuring that have a 'buddy' within the research team monitoring their activities and whereabouts. The interviews may raise sensitive issues such as workload and GP overburden or burnout: the interview study information sheet will provide appropriate sources for accessing confidential support. GPs will be reminded that they have the right to not answer any question, stop the interview or withdraw from the interview study; if there is insufficient time to fully discuss issues GPs will be offered a follow-up time to complete the interview.

GP coding behaviour: It is possible that the eRATs will impact GP coding behaviour - GP coding behaviour for cancer specific symptoms may increase; this would cause a minor increase in triggering of eRATs. We will explore the impact of eRATs on coding behaviour in the interviews (see above) and, resources permitting, will also examine the impact on coding rates using the following approach. We will purposively sample 12 intervention practices and 12 control practices in the South/South West of England based on two-week wait referral rate (i.e., 4 low, 4 moderate, 4 high referring practices) and which software system is being used. In the first instance we will invite practices who are participating in the nested study to support this work. If insufficient numbers agree, we will approach other practices who are not participating in the nested studies. We will explore the rate of coding of the most frequent symptom for each eRAT cancer in the study that underpins that particular cancer (e.g. cough, abdominal pain, haematuria)(22-25) for a month in the first three months of entry into ERICA, and for the same calendar month a year and two years later (as some symptoms have seasonal

For peer review only

variation). This will be performed retrospectively, by the search code being given to the research champion, who will arrange for the search to be conducted in the practice. The results of the search will be emailed to the research team.

Patient experience of care: We will adopt a phased, targeted recruitment strategy with an aim to purposively sample up to (based on two-week rate referral rate (low vs. medium vs. high); gender, age (40-60 vs. 60+)) 32 patients from the intervention arm. We will approach five practices at a time (and expect to recruit around 20 practices to reach the target number of participants), to ensure that we can interview participants in a timely manner.

The in-practice eRAT reports are the mechanism by which we will be able to identify individuals to invite to participate in the activities associated with the process evaluation. The local (at practice) reporting mechanism will allow the research team to identify individuals for whom the eRATs were used and thus who are potentially eligible to participate in a semi-structured interview. Purposive sampling will take place – practices will hold the master eRAT report containing both the patients practice ID number and the new patient study ID. The research team will let the practice know the patient study IDs for those whom an invitation letter will be sent. Practices will be offered remuneration of nearly £200 for the additional work.

Via the GP practice, the research team will send out a letter and information booklet to the identified patients to invite participation in an interview to discuss their experience of care. We will adopt a longitudinal case study design (26) – patients' care pathways will differ, some will receive referrals into secondary care for investigations and tests, while some will be on a 'watch and wait' plan, revisiting their GP at an agreed interval. Some patients will have tests for cancer and the test will indicate that there is no cancer (false positives) whereas some patients will be diagnosed with cancer. So that we can fully capture all patient groups at different stages of their care, individuals will be invited for repeat interviews at regular intervals (i.e., at least one month apart and no more than 3 interviews within 12 months).

We aim to perform the first interview within one month of the consultation in which an eRAT was triggered. Written information about the interview study will have been provided and written informed consent will be taken prior to all interviews, and will be verbally confirmed before the interview commences. Interviews will be audio-recorded and carried out by members of the research team using pre-defined topic guides. The initial interview will be conducted face-to-face at the participant's home or via video conferencing software such as MS Teams at a time convenient for the participant, with any subsequent interview conducted either face-to-face, over the phone, or via video

For peer review only

conference software, depending upon the participant's preference. We will monitor the progression of the Covid-19 pandemic and fully adhere to government advice around social distancing and travel. We will not put the research team or participants at risk and will primarily conduct interviews online. If it is safe to conduct face-to-face interviews, the researcher will comply with the lone worker policy, ensuring that have a 'buddy' within the research team monitoring their activities, whereabouts and expected completion time. The interviews may raise anxiety or concerns related to uncertainty about diagnosis during the referral and investigation period or the watch and wait period; or psychological distress associated with a cancer diagnosis or a false-positive result. The interview study information sheet will provide appropriate sources for accessing confidential support and patients will be reminded that they have the right to not answer any question, stop the interview or withdraw from the interview study.

Management of adverse consequences

As a result of being referred for tests or investigations there is a risk of an adverse incident. If referral rates do increase as a result of access to eRATs, there is an increased risk of an adverse event (AE) to patients of practices allocated to the intervention. We are not routinely tracking individuals throughout the trial and there is no mechanism for monitoring any AEs as a result of referral. However, psychological distress may be a consequence of referral. Individuals for whom cancer is diagnosed at an early stage may be relieved by the diagnosis and see the psychological distress as justifiable. Individuals for whom a referral does not lead to a diagnosis of cancer (false positives) may have undergone unnecessary psychological distress. Our process evaluation work will help us to understand the extent of this and its potential impact on the individuals' life.

During interviews, patients may report being distressed – either as a result of research activity or as a result of their health, and events in their private lives. Should such a situation arise, the researchers will implement the trial risk protocol and manage the participant in accordance with this policy. Participants will be reminded that they have the right to not answer any question, stop the interview or withdraw from the interview study. Under high-risk situations (e.g. where there is perceived immediate risk to a participant's health), the study team may be required to break confidentiality, to inform appropriate authorities who will need to provide essential care services. We will also signpost participants to sources of support. This information will be outlined in the Participant Information Sheet. Participants will be informed of possible benefits and known risks of participation in the interviews by means of a Patient Information Sheet and through discussion with the research team. Written consent will be obtained immediately prior to the interview study.

For peer review only

There are minimal risks to researchers as most interviews will take place in the GP practices or by telephone/online; however, if a home visit is undertaken to interview patient participants the researcher will follow the lone worker policy: researchers will make sure that their whereabouts, contact telephone number and estimated time of return are known to their colleagues and/or manager. Researchers will also have the opportunity to debrief with a senior colleague on the research team should they need any support after conducting an interview; this debrief may be in person or by telephone.

Analysis

For the quantitative results the individual data sources will be summarised descriptively, including a summary of data completeness. For the qualitative data we will adopt a framework approach (27) which allows the inclusion of key concepts and ideas identified from the literature, alongside themes emerging from the data. The framework approach produces a structured output matrix, with cells of data organised by practice and by code (a descriptive label applied to a section of transcript).

At least two researchers will work on the analysis. Interviews will be audio recorded, transcribed and anonymised. Data familiarisation will be achieved through the listening to and reading of interview recordings and transcripts. Transcripts will be imported into the qualitative data analysis software package NVivo 11 (QSR International) to facilitate data management, sharing and development of a coding framework. A proportion of the interview transcripts will be coded by each researcher. The 'constant comparative method' (28) will be utilised: each incident in the data will be compared with other incidents for similarities and differences and any 'negative cases', where a case does not fit the pattern or cannot be explained by the emerging analysis, will be explored and recorded. Following this initial coding, a PPIE meeting (one for the GP interviews and one for the patient interviews) will be held to discuss the emerging themes from the interviews, and to gain alternative perspectives from the PPIE group on those themes. Following the PPIE meeting, the analytical framework will be developed, incorporating researcher and PPIE perspectives on the results, with a final set of themes and codes being agreed upon.

The analytical framework will be applied to all interview transcripts; one researcher will index all transcripts, with a second researcher indexing a proportion, to check the reliability of the indexing and to ensure that the themes of the framework are being interpreted consistently. Any differences in interpretation will be discussed between the two researchers. Following the indexing process, data will be charted into the structured output matrix, which will summarise the data on each theme from all transcripts. A subsequent meeting of the PPIE group will be held once all of the results from the process evaluation have been gathered to gain a users' perspective of the global findings.

For peer review only

The final step in the process evaluation analysis will be to integrate results from the various mixed method data sources using a triangulation protocol(29) to give a more complete picture once individual data sources have been individually analysed. We plan to create a summary matrix, known as a convergence coding matrix, which summarises the findings from each data source after assessing whether the findings are in agreement, partial agreement or no agreement, or whether the data source is silent for the finding under consideration i.e. when a theme or finding arises from one data set but not another.

Reporting

The process evaluation results will be briefly summarised for inclusion in the main trial report and publication, separate dissemination (reports, presentations and publications) will provide further details of the process evaluation findings.

Appendix F

GP Workload

Background and rationale

GPs manage a high and rising workload of increasingly complex patient care with many competing demands to attend to within ten-minute consultations. (30) This, combined with ongoing recruitment and retention challenges, has contributed to a GP workforce 'crisis'. (31-36) The workload implications for GPs of using electronic tools such as eRATs during consultations is unclear.(37) ERICA provides an opportunity to examine whether the use of eRATs by GPs, and the possible subsequent discussion of cancer risk with patients, may impact consultation length and patient 'flow' through consulting sessions. This nested study aims to explore, in terms of consultation time, the impact of GPs using eRATs on GP workload and patient 'flow' through consulting sessions. It will also explore workload in the week following the index consultation in which an eRAT was activated, when relevant letters may be generated, referrals made, investigations followed through, or clinical discussions engaged in.

Objectives

The specific objectives in respect of consultations and sessions are:

(i) to measure and compare the duration of consultations and sessions in which an eRAT has been activated with consultations where eRATs have not been activated;

For peer review only

(ii) to measure and compare the duration of subsequent consultations in the same session after an eRAT has been activated with consultations in sessions where eRATs have not been activated;

(iii) to explore the frequency of interactions with patients' medical records by a GP in the week following a consultation during which an eRAT was activated.

Methods

An observational quantitative study will be conducted in a sub-sample of ERICA intervention practices to examine the durations of consultations and consulting sessions in which eRATs are activated.

Sample size

The basis for the sub-study sample size calculation is on the number of consultations likely to occur over a two-week period within ERICA practices, in which an eRAT will be 'activated' (i.e. an eRAT prompt is shown and/or clinician uses an eRAT symptom checker). A number of assumptions are of note:

The first assumption is that a half-day GP consulting session, typically lasting four hours and comprised of ten-minute consultations, would be associated with a total of 24 consultations. Second, practices have an average headcount of seven GPs (informed by GP workforce data from NHS Digital). (38) Third, a GP is assumed to work an average of 6.7 half-day consulting sessions per week. (39) An average practice would therefore provide a total of 1,126 GP consultations per week.

Accurate estimations of how often an eRAT will be activated, are not yet established in previous research on usage of cancer decision tools in UK general practice. (40,41) Two clinical members of the research team have estimated that an eRAT may be expected to be activated once per GP, per week. This estimate would suggest that approximately 15% of consulting sessions will involve a consultation where the eRAT tool was activated.

The standard deviation for both the length of a consultation and of a whole consulting session from previous literature was four minutes and 20 minutes respectively. (42-44) Project team discussion concluded that a minimally important difference in time for an individual consultation would be between two and five minutes; for a consulting session this minimally important difference would be approximately 10 minutes.

Statistical power to detect a time difference of between two and five minutes in eRAT consultations versus non-eRAT consultations is also in excess of >80%, even if eRATs are observed to have been activated in just 1:40 consulting (2.5% of sessions), the basis of the most conservative estimate. The

For peer review only

power to detect a difference of 10 minutes in sessions where eRATs have been activated compared with sessions where eRATs have not been activated is >80%, even if eRATs affect only 2.5% of sessions. A two-week observation period would provide sufficient data and power to detect differences in the length of consultations and sessions where an eRAT is activated and those where an eRAT is not activated.

Outcome measures

Primary outcome

The primary outcome is the length of time (in minutes) of consultations. These will be consultations during which an eRAT is activated and also those during which an eRAT is not activated. For the purposes of this sub-study, a consultation is defined as starting when the patient's electronic medical record is opened by a GP, for the purpose of conducting either a face-to-face or telephone/video interaction with the patient, and ending when the record is closed. Home visits will be excluded due to difficulty in accessing accurate time information. Consultations with health professionals who would not make referral decisions (e.g. practice nurses, physiotherapists, pharmacists, healthcare assistants) will also be excluded.

Secondary outcomes

In addition to our primary outcome, we propose to examine the following secondary outcomes:

- The length of time (in minutes) of consulting sessions. For the purposes of this study, a session is defined as a half-day period comprised of individual patients' pre-booked or same-day consultations. The half-day periods are typically 'morning' or 'afternoon', although some practices offer early morning and evening sessions as well. (45)
- The number of instances of opening a patient's electronic medical record in the week following an eRAT being activated.

Practice recruitment

An initial pilot in up to three ERICA intervention practices will be undertaken and plans for data collection methods revisited at that point. Practices will be approached by an invitation email and provided with an information sheet detailing the nature of the study and providing contact details of the researcher. No individual patients will be recruited.

A note on practice recruitment to the nested studies: We expect to recruit up to 91 practices across the nested studies (56 in the health economics nested study, up to 20 in the process evaluation and up to 15 in the sub-study on GP workload) practices. Practices will only be asked to help with one of the health economic nested study, the process evaluation nested study, or the GP workload sub-study.

For peer review only

Data collection

Identifying consultations where an eRAT is activated

The Process Evaluation describes earlier how a local ‘at practice’ report will be run for practices in order to collect patient-level data on eRATs usage. This report will be run for practices recruited to this nested study, covering a two-week period.

Measuring durations of consultations and sessions

The eRATs usage report will provide the start and end time of the tool usage, but not the duration of a consultation. A further search function (developed within SystmOne for this nested study) will provide data on the timings of all consultations occurring between two dates (referred to as the ‘appointments report’). The consultations identified in the eRAT usage report will be cross-referenced with the consultations in the appointments report. A variable will be added to denote which consultations involved an eRAT being activated and which did not.

Measuring workload in the week following an eRAT being activated

The eRATs usage report will identify the relevant patient records for which an audit will be run in SystmOne. The audit will provide data on instances of the records being opened and closed by practice staff during the week following the index activation of an eRAT.

Data analysis

Data will be analysed in Stata. Descriptive statistics summarising participating practices and GPs will be presented. Although practice level data will be presented, it will be anonymised (e.g. practice A, B, etc) to protect the identities of individual practitioners or practices.

The primary analysis of the durations of consultations in which an eRAT is activated, will take the form of a mixed-effects linear regression with random intercepts to account for clustering within GPs and for GPs clustering within practices. This regression will adjust for consulting GP, time of day, day of week, and consultation mode (face-to-face, telephone, video). Residuals will be checked for normality. As duration data are typically not normally distributed, the data will be transformed if needed, using log transformation. Bootstrapping of the data will also be undertaken if needed. Similar mixed-effects linear regression models with random intercepts will also be performed for secondary outcomes; the duration of consulting sessions, and the number of instances of opening a patient’s electronic medical record in the week following an eRAT being activated. For all models where duration is the outcome linear models will be used, but for the count of opening medical records Poisson models will be used.

For peer review only

Governance and ethical considerations

Consent

Individual patient consent is not sought within ERICA for the running of the eRAT usage report. The reports in SystmOne, described for this nested study, will not contain identifiable patient data nor clinically sensitive information and so patient consent for these reports will also not be sought.

Data protection/management and confidentiality

The eRAT usage report and the SystmOne reports will contain pseudo-anonymised data: a patient identifier. However, the reports will contain variables denoting date, time and consulting GP, which will allow cross-referencing, so practices will be asked to delete the patient identifier before sending the report securely and electronically to a secure Exeter CTU computer using a predetermined practice ID number. These measures will ensure the data are anonymised. In the event that the researcher visits the practice to run the SystmOne reports, the files will be anonymised in the same way before the researcher leaves. Practices will keep the original 'master' report files containing the patient's practice computer ID.

Finance

The additional work for the nested study, outside of ERICA costs, is for practices to run the reports in SystmOne and send the report files securely to the researcher. Alternatively, the researcher will visit the practice to run the reports, which may require time of a practice administrator or manager for logging in to the clinical system and orientation. In both scenarios, this time would be covered by nested study research costs at a rate of £50 per hour, and each practice will be offered reimbursement for up to 2 hours. Travel costs for the researcher to visit practices where needed are estimated at £0.45 per mile for a 75 mile round-trip per practice (South West).

For peer review only

References

1. Neal RD, Din NU, Hamilton W, Ukoumunne OC, Carter B, Stapley S, et al. Comparison of cancer diagnostic intervals before and after implementation of NICE guidelines: analysis of data from the UK General Practice Research Database. Br J Cancer. 2014;110(3):584-92.
2. Pickard AS, Neary MP, Cella D. Estimation of minimally important differences in EQ-5D utility and VAS scores in cancer. Health and quality of life outcomes. 2007;5:70-.
3. Kind P, Hardman G, Macran S. UK population norms for EQ-5D. Centre for Health Economics, University of York; 1999.
4. Man M-S, Chaplin K, Mann C, Bower P, Brookes S, Fitzpatrick B, et al. Improving the management of multimorbidity in general practice: protocol of a cluster randomised controlled trial (The 3D Study). BMJ Open. 2016;6(4):e011261.
5. Curtis L. Unit Costs of Health and Social Care 2020 [Available from: https://www.pssru.ac.uk/project-pages/unit-costs/unit-costs-2020/]. 2020.
6. Department of Health and Social Care. NHS reference costs 2019 to 2020 [Available from: https://improvement.nhs.uk/resources/reference-costs/]. 2020.
7. Caro JJ, Briggs AH, Siebert U, Kuntz KM. Modeling good research practices--overview: a report of the ISPOR-SMDM Modeling Good Research Practices Task Force-1. Medical decision making : an international journal of the Society for Medical Decision Making. 2012;32(5):667-77.
8. Siebert U, Alagoz O, Bayoumi AM, Jahn B, Owens DK, Cohen DJ, et al. State-Transition Modeling:A Report of the ISPOR-SMDM Modeling Good Research Practices Task Force–3. Medical Decision Making. 2012;32(5):690-700.
9. Schwander B, Hiligsmann M, Nuijten M, Evers S. Systematic review and overview of health economic evaluation models in obesity prevention and therapy. Expert review of pharmacoeconomics & outcomes research. 2016;16(5):561-70.
10. Wang BC, Furnback W. Modelling the long-term outcomes of bariatric surgery: A review of cost-effectiveness studies. Best practice & research Clinical gastroenterology. 2013;27(6):987-95.
11. Andronis L, Barton P, Bryan S. Sensitivity analysis in economic evaluation: an audit of NICE current practice and a review of its use and value in decision-making. Health Technol Assess. 2009;13(29):iii, ix-xi, 1-61.
12. Fenwick E, O'Brien BJ, Briggs A. Cost-effectiveness acceptability curves--facts, fallacies and frequently asked questions. Health economics. 2004;13(5):405-15.
13. Husereau D, Drummond M, Petrou S, Carswell C, Moher D, Greenberg D, et al. Consolidated Health Economic Evaluation Reporting Standards (CHEERS) statement. Value Health. 2013;16(2):e1-5.
14. NICE. Guide to the Methods of Technology Appraisals, Section 5.6 Discounting [Available from: https://www.nice.org.uk/process/pmg9/chapter/the-reference-case#discounting]. 2013.
15. Faria R, Gomes M, Epstein D, White IR. A guide to handling missing data in cost-effectiveness analysis conducted within randomised controlled trials. PharmacoEconomics. 2014;32(12):1157-70.
16. Brazier J, Longworth L. NICE DSU Technical Support Document 8: An introduction to the measurement and valuation of health for NICE submissions. In: Health, editor. 2011.
17. Tengs TO, Wallace A. One thousand health-related quality-of-life estimates. Medical care. 2000;38(6):583-637.
18. NHS England. (2019) The NHS Long Term Plan [Available from: https://www.longtermplan.nhs.uk/.
19. Michie S, van Stralen MM, West R. The behaviour change wheel: a new method for characterising and designing behaviour change interventions. Implementation science : IS. 2011;6:42-.
20. Grant A, Treweek S, Dreischulte T, Foy R, Guthrie B. Process evaluations for cluster-randomised trials of complex interventions: a proposed framework for design and reporting. Trials. 2013;14:15.
21. Hawe P, Shiell A, Riley T. Complex interventions: how "out of control" can a randomised controlled trial be? BMJ (Clinical research ed). 2004;328(7455):1561-3.
22. Shephard EA, Stapley S, Neal RD, Rose P, Walter FM, Hamilton WT. Clinical features of bladder cancer in primary care. The British journal of general practice : the journal of the Royal College of General Practitioners. 2012;62(602):e598-604.
23. Shephard E, Neal R, Rose P, Walter F, Hamilton WT. Clinical features of kidney cancer in primary care: a case-control study using primary care records. The British journal of general practice : the journal of the Royal College of General Practitioners. 2013;63(609):e250-5.

For peer review only - http://bmjopen.bmj.com/site/about/guidelines.xhtml

24. Hamilton W. The CAPER studies: five case-control studies aimed at identifying and quantifying the risk of cancer in symptomatic primary care patients. Br J Cancer. 2009;101 Suppl 2:S80-6.
25. Stapley S, Peters TJ, Neal RD, Rose PW, Walter FM, Hamilton W. The risk of oesophago-gastric cancer in symptomatic patients in primary care: a large case-control study using electronic records. Br J Cancer. 2013;108(1):25-31.
26. Yin R. Case Study Research: Design and Methods. London: Sage Publications 2003.
27. Ritchie J, Spencer L, O'Connor W. Carrying out Qualitative Analysis. In: Ritchie J, Lewis J, editors. Qualitative Research Practice, A guide for Social Science Students and Researchers London: Sage; 2003.
28. Corbin J, Strauss A. Basics of qualitative research: Techniques and procedures for developing grounded theory. London: Sage publications; 2014.
29. O'Cathain A, Murphy E, Nicholl J. Three techniques for integrating data in mixed methods studies. BMJ. 2010;341:c4587.
30. Hobbs FDR, Bankhead C, Mukhtar T, Stevens S, Perera-Salazar R, Holt T, et al. Clinical workload in UK primary care: a retrospective analysis of 100 million consultations in England, 2007-14. Lancet 2016;387:2323-30. http://dx.doi.org/10.1016/S0140-6736(16)00620-6
31. Baird B, Charles A, Honeyman M, Maguire D, Das P. Understanding pressures in general practice: The Kings Fund; 2016.
32. Campbell J CR, Sansom A. Retaining the experienced GP workforce in direct patient care (ReGROUP): final report. Exeter: University of Exeter Medical School; 2015.
33. Sansom A, Terry R, Fletcher E, Salisbury C, Long L, Richards SH, et al. Why do GPs leave direct patient care and what might help to retain them? A qualitative study of GPs in South West England. BMJ Open 2018;8:e019849. http://dx.doi.org/10.1136/bmjopen-2017-019849
34. Roland M, Everington S. Tackling the crisis in general practice. BMJ 2016;352:i942. http://dx.doi.org/10.1136/bmj.i942
35. Fletcher E, Abel GA, Anderson R, Richards SH, Salisbury C, Dean SG, et al. Quitting patient care and career break intentions among general practitioners in South West England: findings of a census survey of general practitioners. BMJ Open 2017;7:e015853. http://dx.doi.org/10.1136/bmjopen-2017-015853
36. Walker B, Moss, C., Gibson, J., Sutton, M., Spooner, S., Checkland, K. Tenth National GP Worklife Survey. Manchester: Policy Research Unit in Commissioning and the Healthcare System Manchester Centre for Health Economics; 2019.
37. Fletcher E, Burns, A., Wiering, B., Lavi, D., Shephard, E., Hamilton, W., Campbell, J. L., Abel, G. Workload and workflow implications associated with the use of electronic risk assessment tools used by health professionals in general practice: a scoping review. In: University of Exeter Medical School; 2022.
38. NHS Digital. General Practice Workforce 30 September 2019. NHS Digital; 2019. URL: https://digital.nhs.uk/data-and-information/publications/statistical/general-and-personal-medical-services/final-30-september-2019#resources (Accessed 11/02/20).
39. Gibson J, Sutton M, Spooner S, Checkland K. Ninth National GP Worklife Survey 2017. In; 2017.
40. Moffat J, Ironmonger L. Clinical Decision Support Tool for Cancer (CDS) Project evaluation report to the Department of Health; 2014.
41. Robinson R, Poirier V, Watson S. Using Cancer Decision Support Tools to support the early diagnosis of cancer; 2017.
42. Salisbury C, Procter S, Stewart K, Bowen L, Purdy S, Ridd M, et al. The content of general practice consultations: cross-sectional study based on video recordings. British Journal of General Practice 2013;63:e751. http://dx.doi.org/10.3399/bjgp13X674431
43. Elmore N, Burt J, Abel G, Maratos FA, Montague J, Campbell J, et al. Investigating the relationship between consultation length and patient experience: a cross-sectional study in primary care. Br J Gen Pract 2016;66:e896-e903. http://dx.doi.org/10.3399/bjgp16X687733
44. Orton PK, Pereira Gray D. Factors influencing consultation length in general/family practice. Fam Pract 2016;33:529-34. http://dx.doi.org/10.1093/fampra/cmw056
45. NHS. Working life (GP). NHS; 2022. URL: https://www.healthcareers.nhs.uk/explore-roles/doctors/roles-doctors/general-practice-gp/working-life (Accessed 2022).

For peer review only

SPIRIT

STANDARD PROTOCOL ITEMS: RECOMMENDATIONS FOR INTERVENTIONAL TRIALS

SPIRIT 2013 Checklist: Recommended items to address in a clinical trial protocol and related documents*

Section/item	Item No	Description	Addressed on page number
**Administrative information**			
Title	1	Descriptive title identifying the study design, population, interventions, and, if applicable, trial acronym	Title page
Trial registration	2a	Trial identifier and registry name. If not yet registered, name of intended registry	1 & 15
	2b	All items from the World Health Organization Trial Registration Data Set	n/a
Protocol version	3	Date and version identifier	1 & 15
Funding	4	Sources and types of financial, material, and other support	15
Roles and responsibilities	5a	Names, affiliations, and roles of protocol contributors	15
	5b	Name and contact information for the trial sponsor	1
	5c	Role of study sponsor and funders, if any, in study design; collection, management, analysis, and interpretation of data; writing of the report; and the decision to submit the report for publication, including whether they will have ultimate authority over any of these activities	12
	5d	Composition, roles, and responsibilities of the coordinating centre, steering committee, endpoint adjudication committee, data management team, and other individuals or groups overseeing the trial, if applicable (see Item 21a for data monitoring committee)	12-13

For peer review only

Introduction

Background and rationale	6a	Description of research question and justification for undertaking the trial, including summary of relevant studies (published and unpublished) examining benefits and harms for each intervention	2-3
	6b	Explanation for choice of comparators	5-7
Objectives	7	Specific objectives or hypotheses	3-4
Trial design	8	Description of trial design including type of trial (eg, parallel group, crossover, factorial, single group), allocation ratio, and framework (eg, superiority, equivalence, noninferiority, exploratory)	4

Methods: Participants, interventions, and outcomes

Study setting	9	Description of study settings (eg, community clinic, academic hospital) and list of countries where data will be collected. Reference to where list of study sites can be obtained	4
Eligibility criteria	10	Inclusion and exclusion criteria for participants. If applicable, eligibility criteria for study centres and individuals who will perform the interventions (eg, surgeons, psychotherapists)	7
Interventions	11a	Interventions for each group with sufficient detail to allow replication, including how and when they will be administered	4-5
	11b	Criteria for discontinuing or modifying allocated interventions for a given trial participant (eg, drug dose change in response to harms, participant request, or improving/worsening disease)	n/a
	11c	Strategies to improve adherence to intervention protocols, and any procedures for monitoring adherence (eg, drug tablet return, laboratory tests)	14 and Appx D
	11d	Relevant concomitant care and interventions that are permitted or prohibited during the trial	4-7
Outcomes	12	Primary, secondary, and other outcomes, including the specific measurement variable (eg, systolic blood pressure), analysis metric (eg, change from baseline, final value, time to event), method of aggregation (eg, median, proportion), and time point for each outcome. Explanation of the clinical relevance of chosen efficacy and harm outcomes is strongly recommended	8-9
Participant timeline	13	Time schedule of enrolment, interventions (including any run-ins and washouts), assessments, and visits for participants. A schematic diagram is highly recommended (see Figure)	6

For peer review only - http://bmjopen.bmj.com/site/about/guidelines.xhtml

Sample size	14	Estimated number of participants needed to achieve study objectives and how it was determined, including clinical and statistical assumptions supporting any sample size calculations	6-7
Recruitment	15	Strategies for achieving adequate participant enrolment to reach target sample size	7
**Methods: Assignment of interventions (for controlled trials)**			
Allocation:			
Sequence generation	16a	Method of generating the allocation sequence (eg, computer-generated random numbers), and list of any factors for stratification. To reduce predictability of a random sequence, details of any planned restriction (eg, blocking) should be provided in a separate document that is unavailable to those who enrol participants or assign interventions	7
Allocation concealment mechanism	16b	Mechanism of implementing the allocation sequence (eg, central telephone; sequentially numbered, opaque, sealed envelopes), describing any steps to conceal the sequence until interventions are assigned	7
Implementation	16c	Who will generate the allocation sequence, who will enrol participants, and who will assign participants to interventions	7
Blinding (masking)	17a	Who will be blinded after assignment to interventions (eg, trial participants, care providers, outcome assessors, data analysts), and how	7-8
	17b	If blinded, circumstances under which unblinding is permissible, and procedure for revealing a participant's allocated intervention during the trial	n/a
**Methods: Data collection, management, and analysis**			
Data collection methods	18a	Plans for assessment and collection of outcome, baseline, and other trial data, including any related processes to promote data quality (eg, duplicate measurements, training of assessors) and a description of study instruments (eg, questionnaires, laboratory tests) along with their reliability and validity, if known. Reference to where data collection forms can be found, if not in the protocol	9-12
	18b	Plans to promote participant retention and complete follow-up, including list of any outcome data to be collected for participants who discontinue or deviate from intervention protocols	11-12

For peer review only - http://bmjopen.bmj.com/site/about/guidelines.xhtml

Data management	19	Plans for data entry, coding, security, and storage, including any related processes to promote data quality (eg, double data entry; range checks for data values). Reference to where details of data management procedures can be found, if not in the protocol	11-12
Statistical methods	20a	Statistical methods for analysing primary and secondary outcomes. Reference to where other details of the statistical analysis plan can be found, if not in the protocol	9-11
	20b	Methods for any additional analyses (eg, subgroup and adjusted analyses)	11
	20c	Definition of analysis population relating to protocol non-adherence (eg, as randomised analysis), and any statistical methods to handle missing data (eg, multiple imputation)	11
**Methods: Monitoring**			
Data monitoring	21a	Composition of data monitoring committee (DMC); summary of its role and reporting structure; statement of whether it is independent from the sponsor and competing interests; and reference to where further details about its charter can be found, if not in the protocol. Alternatively, an explanation of why a DMC is not needed	12-13
	21b	Description of any interim analyses and stopping guidelines, including who will have access to these interim results and make the final decision to terminate the trial	n/a
Harms	22	Plans for collecting, assessing, reporting, and managing solicited and spontaneously reported adverse events and other unintended effects of trial interventions or trial conduct	9, Appendix D
Auditing	23	Frequency and procedures for auditing trial conduct, if any, and whether the process will be independent from investigators and the sponsor	n/a
**Ethics and dissemination**			
Research ethics approval	24	Plans for seeking research ethics committee/institutional review board (REC/IRB) approval	15
Protocol amendments	25	Plans for communicating important protocol modifications (eg, changes to eligibility criteria, outcomes, analyses) to relevant parties (eg, investigators, REC/IRBs, trial participants, trial registries, journals, regulators)	12-13

For peer review only - http://bmjopen.bmj.com/site/about/guidelines.xhtml

Consent or assent	26a	Who will obtain informed consent or assent from potential trial participants or authorised surrogates, and how (see Item 32)	11 & Appendices B & D (only relevant for nested studies)
	26b	Additional consent provisions for collection and use of participant data and biological specimens in ancillary studies, if applicable	11 & Appendices B & D (only relevant for nested studies)
Confidentiality	27	How personal information about potential and enrolled participants will be collected, shared, and maintained in order to protect confidentiality before, during, and after the trial	11-12
Declaration of interests	28	Financial and other competing interests for principal investigators for the overall trial and each study site	15
Access to data	29	Statement of who will have access to the final trial dataset, and disclosure of contractual agreements that limit such access for investigators	14
Ancillary and post-trial care	30	Provisions, if any, for ancillary and post-trial care, and for compensation to those who suffer harm from trial participation	n/a
Dissemination policy	31a	Plans for investigators and sponsor to communicate trial results to participants, healthcare professionals, the public, and other relevant groups (eg, via publication, reporting in results databases, or other data sharing arrangements), including any publication restrictions	14
	31b	Authorship eligibility guidelines and any intended use of professional writers	14
	31c	Plans, if any, for granting public access to the full protocol, participant-level dataset, and statistical code	14
**Appendices**			
Informed consent materials	32	Model consent form and other related documentation given to participants and authorised surrogates	Not applicable for main trial. Multiple documents for each nested study, available from authors upon request

For peer review only - http://bmjopen.bmj.com/site/about/guidelines.xhtml

Biological specimens	33	Plans for collection, laboratory evaluation, and storage of biological specimens for genetic or molecular analysis in the current trial and for future use in ancillary studies, if applicable	n/a

*It is strongly recommended that this checklist be read in conjunction with the SPIRIT 2013 Explanation & Elaboration for important clarification on the items. Amendments to the protocol should be tracked and dated. The SPIRIT checklist is copyrighted by the SPIRIT Group under the Creative Commons "Attribution-NonCommercial-NoDerivs 3.0 Unported" license.

For peer review only
